# Germline Mutation Landscape and Associated Clinical Characteristics in Chinese Patients With Renal Cell Carcinoma

**DOI:** 10.3389/fonc.2021.737547

**Published:** 2021-12-02

**Authors:** Wen Kong, Tongtong Yang, Xiaodong Wen, Zhongyi Mu, Cheng Zhao, Sujun Han, Jing Tian, Xinhao Zhang, Tao Zhou, Yanrui Zhang, Feng Lou, Shanbo Cao, Huina Wang, Jin Zhang

**Affiliations:** ^1^ Department of Urology, Renji Hospital, Shanghai Jiao Tong University School of Medicine, Shanghai, China; ^2^ Department of Translational Medicine, Acornmed Biotechnology Co., Ltd, Tianjin, China; ^3^ Department of Urology, Shanxi Provincial People’s Hospital, Taiyuan, China; ^4^ Department of Urology, Liaoning Cancer Hospital, Shenyang, China; ^5^ Department of Urology, Xiangya Hospital of Central South University, Changsha, China; ^6^ Department of Urology, National Cancer Center/Cancer Hospital, Chinese Academy of Medical Sciences and Peking Union Medical College, Beijing, China; ^7^ Department of Urology, The 2^nd^ Hospital of Tianjin Medical University, Tianjin, China

**Keywords:** renal cell carcinoma, germline mutations, Chinese population, pathogenic variation, second hit events

## Abstract

**Background:**

Renal cell carcinoma (RCC) is a disease of genomic alterations, of which the complete panorama helps in facilitating molecular-guided therapy. Germline mutation profiles and associated somatic and clinical characteristics remains unexplored in Chinese RCC patients.

**Methods:**

We retrospectively profiled the germline and somatic mutations of 322 unselected RCC patients using a panel consisting of 808 cancer-related genes. We categorized patients into three groups based on germline mutation status and compared the somatic mutation spectrum among different groups.

**Results:**

Approximately one out of ten (9.9%) RCC patients were identified to carry pathogenic/likely pathogenic (P/LP) germline variants (PGVs), of which 3.7% were variants in syndromic RCC-associated genes and 6.2% were other cancer-predisposition genes. The most common PGV was found in *VHL* (2.2%), followed by *FH, TSC2, ATM, BRCA1, NBN, and BLM* (0.6% each). Young patients (≤46 years) were more likely to harbor PGVs. Variants in syndromic RCC-associated genes were predominant identified in young patients, while variants in other cancer-predisposition genes were found in patients >46 years more frequently. Furthermore, 39.3% (11/28) of patients carrying PGVs were detected to have somatic “second hit” events. Germline and somatic sequencing, including microsatellite instability (MSI) status analysis, provided potentially actionable therapeutic targets in 17.1% of patients in the whole cohort.

**Conclusions:**

Our results revealed that approximately 10% of RCC patients carried clinically significant germline mutations. Current guidelines recommendation for genetic testing seemed not sensitive enough to identify patients with hereditary RCC susceptibility. It is rational to promote genetic testing in RCC population.

## Introduction

Renal cell carcinoma (RCC) is one of the most common malignancies worldwide, which is estimated to cause over 13000 and 25600 deaths per year in the United States and China respectively (1, 2). The incidence continues to increase by approximately 2.5% every year with the highest rates in developed countries (3), which is believed to attribute to advances in diagnostic imaging and also prevalence of cancer associated risk factors such as smoking, obesity and hypertension, et al. (4).

Notably, hereditary factors play important roles in the RCC tumorigenesis. Approximately 3-17% of RCC is considered to have hereditary susceptibility (5–9), however the reported germline mutation frequency in RCC patients can be affected by different ethnicities, pathological subtypes, clinical stages of the disease and also selection criteria for study population. A recent study showed that germline mutations in caner-predisposition genes were found in 16.1% of non-Hispanic white patients with advanced kidney cancer (10). In comparison, deleterious germline mutations were detected in 9.5% of Chinese patients with early-onset RCC (11). In a multi-ethnic large cohort study, pathogenic/likely pathogenic (P/LP) variants were found in 17% of 1829 patients and *FH* variants were found to be significantly enriched in patients of African ancestry (9). Therefore, the prevalence of hereditary susceptibility in the Chinese kidney cancer population needs to be carefully clarified in detail. Identification of germline susceptibility is crucial for risk assessment, early detection and treatment decisions for RCC patients and their close relatives. Despite potential benefits of identifying germline mutation carriers in RCC-affected individuals, the candidate population for genetic testing remains unclear.

There has been great progress in hereditary RCC research in the past decades. A number of hereditary RCC syndromes have been identified (12), such as Von Hippel-Lindau (VHL) disease, Birt-Hogg-Dubé (BHD) syndrome, Hereditary Leiomyomatosis and Renal Cell Carcinoma (HLRCC), and Hereditary Papillary RCC (HPRC), which is caused by germline mutations in *VHL, BHD, FH* and *MET*, respectively. Some other rare cancer syndromes caused by germline mutations in *BAP1, SDHA/B/C/D, TSC1/2* and *MITF* etc. also predispose patients to RCC (13, 14). Germline mutations in *TP53*, *BRCA1*, *BRCA2*, and *CHEK2* genes have been reported to associate with certain hereditary cancer syndromes and would probably increase the risk of RCC (10, 15, 16). Advance and availability of next-generation sequencing (NGS) technology is expanding our understanding of hereditary kidney cancer.

Large-scale somatic studies have shed lights on the mechanisms of RCC tumorigenesis (17). *VHL, PBRM1, BAP1* and *SETD2* were identified as most commonly mutated genes in clear cell RCC, whereas *MET, CDKN2A/B, TERT* and *FH* mutations were identified in papillary RCC and other non-clear cell RCC. Clinical and mechanistic research correlated these characteristic mutations with patients’ prognosis and response to therapies (18). Integration of germline and somatic landscape in RCC patients has rarely been studied (10). However, all the information underlines the importance of delineating a comprehensive landscape of molecular architecture for inherited RCC syndromes.

The present study evaluated germline mutations of cancer-associated genes in Chinese RCC patients unselected for inherited syndrome risk factors, including age of onset, presence of multifocal lesions, or family history et al. We used NGS to identify the frequency of pathogenic/likely pathogenic germline variants (PGVs) in cancer-associated genes. Somatic mutation profiles were also investigated and integrative analysis of germline and somatic mutations, and correlations with general clinical characteristics was performed. This study applied targeted multigene panel testing to provide a more extensively inclusive identification of possible hereditary RCC patients than previously reported.

## Methods

### Patients and Samples

We retrospectively analyzed de-identified data from 322 patients with a personal diagnosis of RCC who underwent NGS with a targeted exon capture–based assay of 808 cancer-associated genes (**Supplementary Table S1**) at a commercial diagnostic laboratory (AcornMed Biotechnology, Tianjin, China) from January 1, 2019 to March 31, 2020. Immunohistochemistry (IHC) was performed on FFPE tissue with antibodies against mismatch repair (MMR) proteins MLH1, MSH2, MSH6 and PMS2. Patients were not screened for age of onset, multifocal disease, familial cancer history or personal malignant tumor history before enrollment for this retrospective study. Clinical, demographic and pathologic information were obtained from medical records or questionnaire forms before NGS testing, which included sex, age of cancer diagnosis, smoking history and clinical stage. Written informed consent were obtained from all patients.

### Library Construction and Sequencing

Genomic DNA isolated from blood and tumor using the QIAGEN DNeasy Blood and Tissue Kit (Qiagen, Hilden, Germany) following the standard procedures. The concentration and purity of DNA was quantified in Qubit 2.0 (Life Technologies, Carlsbad, CA, USA) and Agilent 2100 Bioanalyzer Instrument (Agilent Technologies, Santa Clara, CA, USA). Then DNA was sheared with a Covaris M220 instrument using the recommended settings for 200-250 bp fragments. Targeted sequencing of both blood and tumor tissue was performed on the same multigene panel. The target sequences of 808 cancer-associated genes were enriched from genomic DNA using Twist Custom Panels (Twist Bioscience, South San Francisco, CA, USA). The NGS libraries were constructed using the KAPA Hyper Prep Kit (KAPA Biosystems, Boston, MA, USA) following the manufacturer’s protocol. The library preparations were sequenced at AcornMed Biotechnology (Tianjin, China) using an Illumina NovaSeq 6000 platform (Illumina, San Diego, CA, USA), and 150 bp paired-end reads were generated. Mean sequencing coverage was 500× for tumor tissue and 100× for white blood cell. The complete DNA sequence was aligned to human genome hg19 (UCSC) using BWA (Burrows-Wheeler Alignment tool) software (19) After removing potential PCR duplicates by applied Picard (http://Picard.Sourceforge.net), variant calling was performed by the Genome Analysis Toolkit(GATK 3.8-1-0) (20).

### Mutation Analysis and Interpretation

Germline variants in 808 cancer-associated genes included in the panel were further analyzed. Germline single nucleotide variation (SNV) was called using the Haplotype Caller module in GATK. Insertions and deletions (InDels) were detected by the GATK Unified Genotyper. To accurately detect reliable SNVs and InDels, we used rigorous set of strict filtering standard (21): ≥5 reads covering the mutated sites, with at least three reads harboring the mutations; (2) allele frequency falling in 0.3-0.7; (3) frequency <1% in any population database, including ExAC (http://exac.broadinstitute.org), 1000 Genomes (http://www.1000genomes.org) or ESP6500 databases (https://evs.gs.washington.edu/EVS); (4) the mutation frequency <5% in our cohort. For further pathogenicity analysis, we excluded variants without functional relevance, including non-exonic variants (except splice variants) and synonymous variants. The potential P/LP mutations are defined as (21): all truncating mutations and non-frameshift mutations which affect more than three amino acids; (2) missense mutations were predicted deleterious by SIFT, CADD and Polyphen2 software; (3) all variants identified as P/LP mutations in ClinVar or Disease-causing mutation (DM)/Disease-causing mutation (DP)/Disease associated Polymorphism (FP)/functional polymorphism (DFP), Diseaseassociated polymorphism with functional evidence (DFP) in HGMD database. The potential P/LP mutations were validated by first-generation sequencing, and pathogenicity was then manually determined by molecular pathologists according to American College of Medical Genetics (22) standards (22). dbSNP142 (https://www.ncbi.nlm.nih.gov/SNP/) database was used to assess whether the variation has been reported ever before.

Somatic SNVs were called using the MuTect module in GATK with blood samples from the corresponding individuals as control. InDels were detected by the GATK Unified Genotyper. Loss of heterozygosity (LOH) was evaluated using the Fraction and Allele-Specific Copy Number Estimates from Tumor Sequencing (FACETS) algorithm (23). Large copy-number variants (CNV) were detected by GATK (gatk-4.1.0.0) and the cut-off value for copy number loss ratio was 0.9 (20). All somatic variants (including synonymous mutations)were used to analyze the mutation profiles based on signature modules in Maftools (1.9.30) (24). To accurately detect reliable SNVs and InDels, we used rigorous filtering standard (21): >5 reads covering every individual mutated sites; (2) at least 10× coverage for normal samples was required, with at most one read harboring the mutations; (3) the minimum value of the maximum mapping quality score for mutated alleles was set to 20; (4) the mutation allele frequency ≥1% (21); mutations listed in dbSNP 142 were removed unless they were documented by the Catalog of Somatic Mutations in Cancer (COSMIC). As somatic second-hit events, we considered truncating variants, missense variants predicted as damaing by SIFT & PolyPhen, as well as LOH and large-scale homozygous deletions.

Mutational signature analysis was identified using nonnegative matrix factorization (NMF) from the 96 subtypes of threebase context of mutations following the authors’ guidelines (25). We then compared these signatures against the known SBS signatures from the Catalog of Somatic Mutations in Cancer [COSMIC v3.1, release v91 (June 2020)]. We further applied cosine similarity to identify the best matches within signatures (values> 0.7).

### Data Analysis

The demographic and clinical characteristics were presented using descriptive statistics. To investigate the association of PGVs with clinical factors, we used the chi-squared test or Fisher’s exact test for categorical variables, such as age, sex, smoking status, and clinical stage. All the p-values presented are for a two-tailed test, and *p <*0.05 represented statistical significance. Statistical analysis was performed using SPSS software version 22.0 (SPSS, Inc., Chicago, IL, USA) and R version 3.3.3.

## Results

### Patient Characteristics

**Figure 1:**
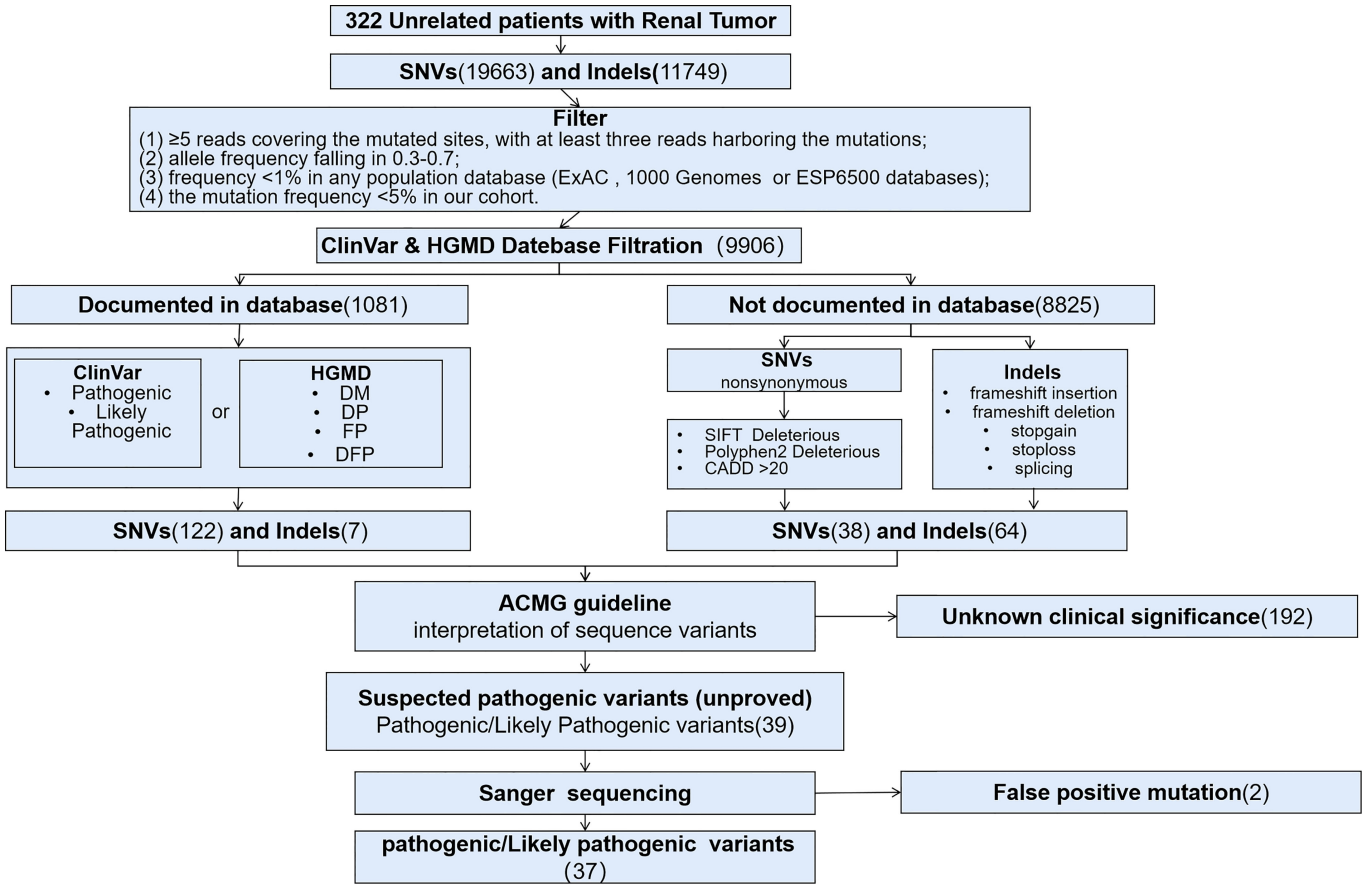
Flow chart of the study design and the filtration of pathogenic variants and variants of unknown clinical significance. DM, Disease-causing mutation; DP, Disease associated Polymorphism; FP, functional polymorphism, DFP, Diseaseassociated polymorphism with functional evidence.

Three hundred and twenty-two patients diagnosed as RCC were included for this retrospective study between January 1, 2019 and March 31, 2020, and all patients were Han Chinese. Targeted sequencing based on Acornmed 808 panel™ were performed in all the qualified tumor tissues and blood samples. DNA extracted from blood leukocytes were analyzed for germline variations. A total of 274 RCC patients with available tumor tissues underwent somatic mutation detection. The majority of included patients were men (217/322, 67.4%). The median age was 56 years (range: 16 to 87 years), and 71 patients (22.0%) were under the age of 46. A total of 128 patients (38.6%) had smoking history. For the clinical stage, the available information revealed that 35.4% of the patients were diagnosed as localized tumors (AJCC Stage I or II), while 37.6% were in advanced disease (AJCC Stage III or IV). The clinical characteristics were described in **Table 1** in detail. Besides, a flowchart was plotted to present the study design and analysis process (**Figure 1**).

**Table 1:** Patient demographic and clinical characteristics.

Characteristic	Cases (n=322)
Sex, No. (%)	
Male	217 (67.4%)
Female	105 (32.6%)
Age of presentation, N (%)	
Median (range), yrs	56 (16-87)
≤46 yrs, No. (%)	71 (22.0%)
>46 yrs, No. (%)	251 (78.0%)
PGVs carrier, No. (%)	32 (9.9%)
Smoking status, No. (%)	129 (40.1%)
Clinical stages, No. (%)	
I	33 (10.2%)
II	74 (23.0%)
III	72 (22.4%)
IV	61 (18.9%)
Unknown	82 (25.5%)

### Prevalence and Spectrum of Pathogenic and Likely Pathogenic Germline Variants

**Figure 2:**
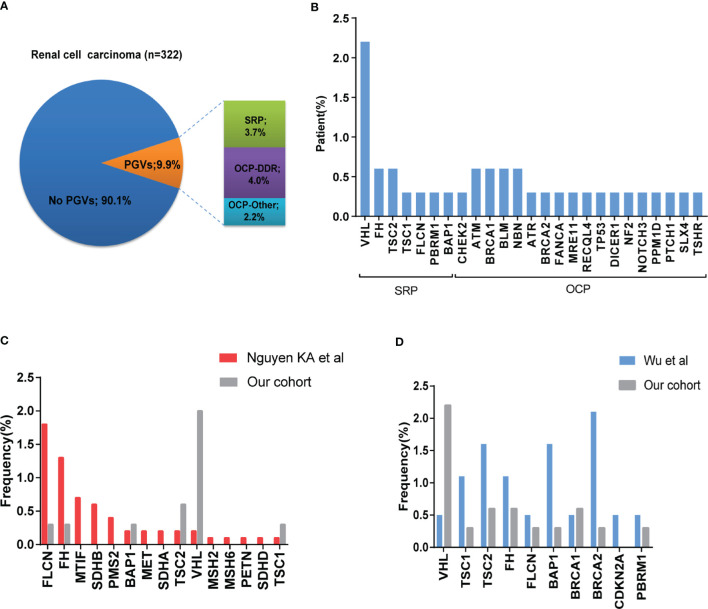
Frequency and spectrum of pathogenic germline mutations in patients with RCC. **(A)** Frequency of germline variants in patients with RCC. Frequency of pathogenic/likely pathogenic (P/LP) germline variants (PGVs) identified in 322 patients with RCC. Pie chart shows that 9.9% (32/322) of patients were identified to have PGVs with 3.7% (n =13) having PGVs in syndromic RCC-associated genes, and 6.3% (n = 19) carrying PGVs in other cancer-associated genes. **(B)** Frequency by gene of PGVs detected in this study. **(C, D)** Comparison of frequency of germline pathogenic/likely pathogenic mutation in our study with that from Nguyen cohort and Wu cohort.

Among 322 patients, 32 (9.9%) were identified to carry 37 PGVs in 25 different cancer-associated genes (**Figure 2A**). Five patients (1.6%) were found to carry more than one PGV in each individual. All PGVs were manually re-checked according to ACMG guideline to ensure fidelity (**Supplementary Table S2**). Most PGVs were frameshift (13/37, 35.1%) and nonsense mutations (13/37, 35.1%), followed by missense (8/37, 21.6%) and splicing mutations (3/37, 8.1%) respectively. Twenty (54.1%) PGVs have not been reported in public database (dpSNP142 and ClinVar), which were considered as novel mutations.

Twelve patients (3.7%) carried germline mutations in syndromic RCC-associated genes (*VHL* (7, 2.2%), *FH* (2, 0.6%), *TSC2* (2, 0.6%), *BAP1* (1, 0.3%), *FLCN* (1, 0.3%), *TSC1* (1, 0.3%)). Twenty patients (6.2%) carried germline mutations in other cancer-predisposition genes which are not clearly associated with RCC. Among these, most (4.0%) were DNA damage repair (DDR)-related genes, including *ATR* (1, 0.3%), *ATM* (2, 0.6%), *BLM* (2, 0.6%), *BRCA1* (2, 0.6%), *BRCA2* (1, 0.3%), *CHEK2* (1, 0.3%), *NBN* (2, 0.6%), *FANCA* (1, 0.3%), *RECQL4* (1, 0.3%), *TP53* (1, 0.3%) and *SLX4* (1, 0.3%), and the remaining 2.2% were non-DDR-related genes including *DICER1* (1, 0.3%), *MRE11* (1, 0.3%), *NF2* (1, 0.3%), *NOTCH3* (1, 0.3%), *PPM1D* (1, 0.3%), *PTCH1* (1, 0.3%), and *TSHR* (1, 0.3%) (**Figure 2B**). One patient carried two different germline variants within *TSC2* gene. One patient carried *BLM* and *MRE11* germline variants, and three patients with *VHL* germline variant were identified to carry *PBRM1*, *BAP1* or *TP53* germline variants at the same time respectively.

To investigate possible difference of PGVs landscape between Chinese and Caucasian population, we compared the frequency of germline variants between previously reported cohorts and ours, mainly focused on genes shared between different panels (26). *VHL* mutation was more common in our study, while *FLCN* and *FH* gene mutations were more frequently detected in Nguyen’s cohort. *PBRM1* gene variant was only detected in our study, while *MITF*, *MET*, *SDHA*, *SDHB*, *SDHD* and MMR genes variants were reported in Nguyen’s cohort (**Figure 2C**). We also compared our results with the previous report from a Chinese early-onset RCC cohort (11). Of note, all cases in their study were younger than 46 years old, while only 22.0% (71/322) were under 46 in our cohort. The most frequently detected germline variants were located in *FH*, *BAP1*, *TSC1* and *TSC2* in their study, while *VHL* was more frequent in ours. Incidences of *FLCN* and *PBRM1* germline mutations were similar between these two cohorts (**Figure 2D**).

### Correlation Analysis Regarding to Clinical Characteristics

Three hundred and twenty-two patients were stratified into three groups based on the germline variant status: Syndromic RCC-associated gene PGVs (SRP) group, Other Cancer-associated gene PGVs (OCP) group and Non-Cancer-associated gene PGVs (NCP) group. Comparison of clinical variables was performed among the above groups (**Supplementary Table S3**). No significant differences about sex, smoking status and RCC clinical stage distribution were discovered among three groups. Notably, the incidence of PGVs was significantly higher in patients younger than 46 years old (16.9% vs. 8.0%, *p* = 0.041) (**Supplementary Figure 1B**). Within patients carrying PGVs, the landscape of germline gene variants was different between patients over 46 years and those younger than 46 years old (**Supplementary Figure 1B**). The proportion of patients ≤46 years was significantly higher in SRP group, in contrast to OCP or NCP (58.3% vs. 25.0% vs. 20.3%, *p*=0.012) (**Supplementary Table S2**). Although the ratio of patients less than 46 years was numerically different between the OCP and the NCP, it was no statistical difference.

### Somatic Variation Analysis

Fresh or FFPE tumor tissue were available in 274 cases, among which there were 28 PGVs carriers. Targeted NGS with 808 genes was performed with tumor DNA to investigate somatic molecular profiles (**Supplementary Table S5**). The top 20 frequently mutated genes in this cohort are listed in **Supplementary Figure 1A**. *VHL* mutation was the most prominent one (51%), followed by *PBRM1* (24%), *SETD2* (14%), *GNAQ* (12%), *BAP1* (11%), *TP53* (9%), *INPPL1* (8%), *ARID1A* (6%), *KMT2D* (6%) and *KMT2B* (6%). Interestingly, somatic mutation frequencies were different in SRP, OCP and NCP groups. Frequency of *VHL*, *SETD2*, *GNAQ* and *INPPL1* mutations were comparable among three group, while *PBRM1*, *ARID1A* and *KMT2C* mutations were only detected in OCP and NCP groups, and somatic *BAP1* mutation was only detected in NCP group. The SRP group had the lowest frequency of typical somatic driver mutations, e.g., *VHL* (8.3% vs. 30.0% vs. 45.5%), *PBRM1* (0.0% vs. 20.0% vs.22.8%), *SETD2* (10% vs. 16.7% vs. 14.6%) (**Supplementary Table S4**). To explore the etiology of RCC tumorigenesis in three groups, we adopted NMF algorithm to identify single base substitution (SBS) signatures. A total four mutational signature with high cosine similarity (>0.7) were identified among three groups. The mutational signatures were SBS 5, SBS 6, and SBS (1, 26) for SPC, OPC and NCP, respectively (**Supplementary Figure 1C**). SBS 1 and 5 are associated with age, and SBS 6 and SBS 26 are signatures of MMR deficiency.

**Figure 3:**
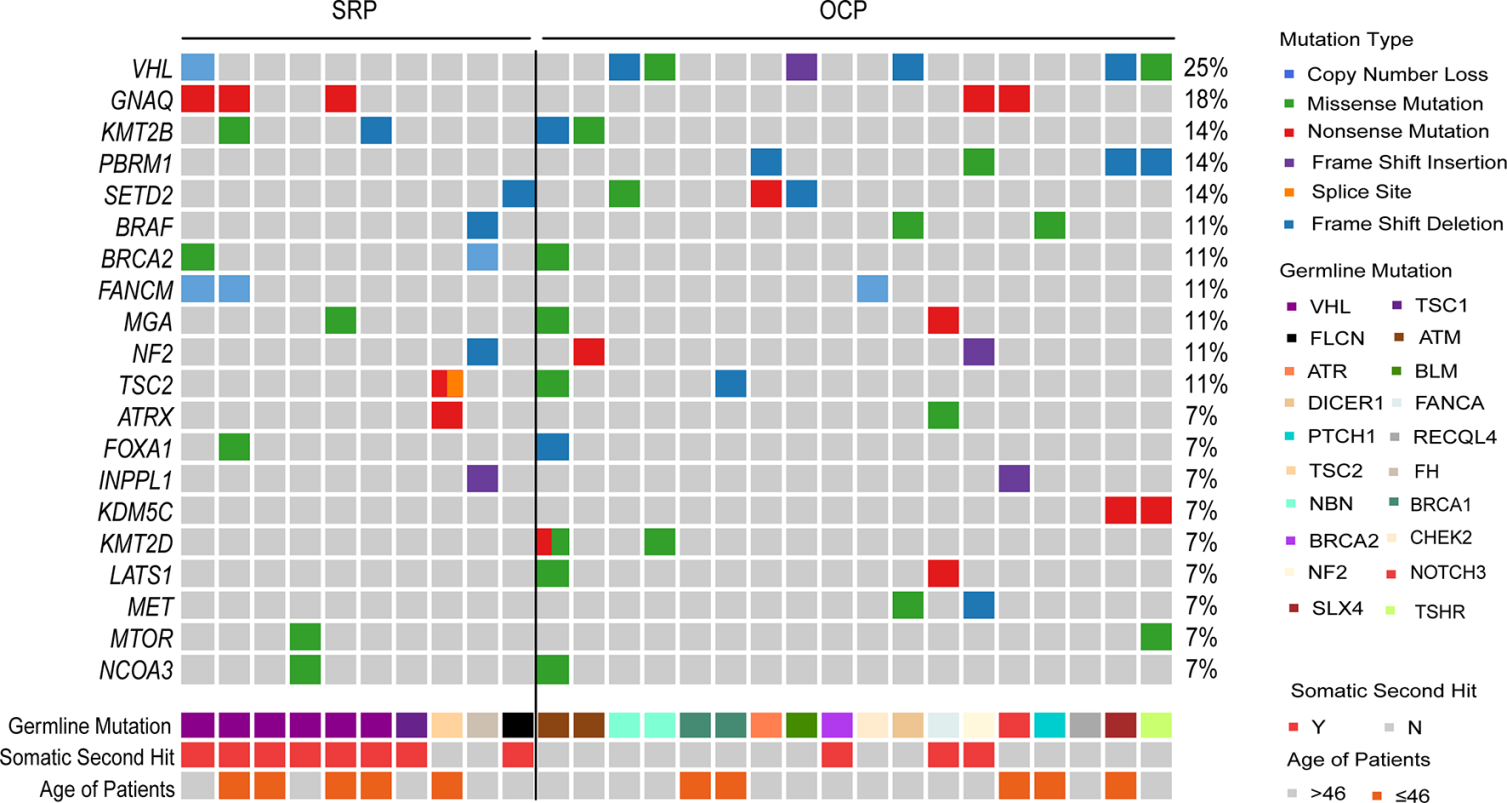
Somatic mutation spectrum of RCC patients with PGVs. Somatic alteration Landscape of 28 RCC patients with PGVs. Top heatmap, distribution of RCC-associated cancer genes and top 20 genes across samples, with genes ranked by mutation frequency. Middle heatmap shows germline mutation genes with somatic hit events presented in the next line. Bottom heatmap shows patient age.

Next, we analyzed the somatic “second hit” events in patients with PGVs, including somatic variants, LOH and large CNV in the interested genes and loci (**Figure 3** and **Supplementary Table S2**). Among 10 patients in SRP group, eight (80%) were identified with somatic “second hit” events. Specifically, six patients with *VHL* PGVs harbored 3p loss, one patient with *FLCN* PGVs had 17p loss, one patient with *TSC1* germline mutation carried *TSC1* somatic mutation. Among 18 patients in OCP group, 17% (3/18) were detected to have somatic “second hit”. One patient with *BRCA2* PGVs demonstrated loss of 13q, one patient with *FANCA* germline mutation was identified to have a LOH, and the third one with *NF2* germline mutation was proved to have 22q loss.

### Clinical Actionable Variation

**Figure 4:**
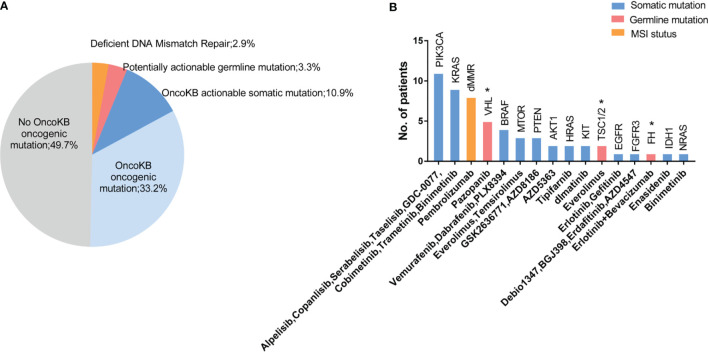
Actionable alterations identified in tumors and germline sequencing. **(A)** 17.1% (47/274) of patients had a potentially actionable genomic alteration, including 10.9% (30/274) somatic alteration, 3.3% (9/274) germline, and 2.9% (8/274) with dMMR signature. 33.2% of patients had OncoKB identified oncogenic mutations without known potentially actionable alterations, and 49.7% of patients were absent with known driver mutations. **(B)** Number of patients with mutations in putative actionable target genes based on OncoKB databases and NCCN guidelines with candidate drugs listed below. *VHL*, *TSC* and *FH* were approvel/indicated for RCC base in NCCN guideline, which were markerd with an “*”.

To determine the potential clinical value of the detected variants, we applied the OncoKB precision oncology knowledge database and National Comprehensive Cancer Network (NCCN) clinical practice guidelines. We analyzed clinically actionable variants in 274 patients with both germline, somatic variation and tumor MSI status to further identify potentially targetable pathways. Overall, 17.1% (47/274) of patients had a potentially actionable genomic alteration, including 10.9% (30/274) somatic alteration, 3.3% (9/274) germline, and 2.9% (8/274) MMR deficiency (dMMR) that could guide standard or investigational therapy selection (**Figure 4A**). Approximately 46.7% (128/274) of patients harbored at least one oncogenic variant, which were recorded or predicted as oncogenic factors according to OncoKB database (**Figure 4A**). The top 5 biomarkers were *PIK3CA*, dMMR, *KRAS*, *VHL* and *BRAF* (**Figure 4B**). Most of these biomarkers were used in other cancers but not routinely applied in RCC (level 3B and 4), and tumor mutation burden was not included in this study.

## Discussion

This retrospective study indicates that approximately every one out of ten RCC patients carries P/LP germline mutations in cancer susceptibility genes. While the prevalence of RCC with hereditary susceptibility (including well-known syndromic RCC and RCC with germline mutation in other cancer-associated genes) reported herein (9.9%) is consistent with previous publications (11), this is the first study, to our knowledge, to determine the prevalence and spectrum of germline mutations in an unselected series of Chinese RCC patients. Among 32 patients with PGVs, 12 (3.7%) had mutations in syndromic RCC-associated genes and 20 (6.2%) carried mutations in other cancer-associated genes. Young patients were more likely to harbor PGVs, both in syndromic RCC-associated genes and other cancer-associated genes. Identification of germline/somatic mutations and tumor MMR status provided information for actionable therapeutic targets in 17.1% of enrolled cases.

The overall prevalence of PGVs was 9.9% in our study. A wide range of PGV prevalence has been reported in literatures, ranging from 3% to 17%, but most of them were limited to patients with selected risk factors, including age of onset, familial tumor history or disease stage et al. (9–11, 27). Although our results are consistent with Wu’s study, their study was restricted to patients with early onset disease (11). Another study from America reported a much lower PGV prevalence of 6.1% (71/1235), and the detailed spectrum of mutations differed from ours (26). It demonstrated that the rate of PGVs in cancer susceptibility genes were obviously influenced by study design and patient enrollment. However, all the results implied a higher frequency of PGVs than ever predicted among patients with unselected patients with RCC.

Considering the incomplete history, indiscernible manifestations, low penetrance, lack of awareness and other difficult circumstances in clinical practice, the value of genetic testing for screening of hereditary tumor syndromes, including hereditary RCC syndromes, becomes recognized in the recent years. The newly updated NCCN guidelines strongly recommended genetic screening for patients predisposed to hereditary RCC, including diagnosed at age ≤46 years old, bilateral/multifocal tumors, familial cancer history or specific histologic characteristics (28, 29). However, issues such as who, when and how to perform testing remain uncertain. At present, histological and clinical features play an important role in the evaluation of genetic tests. A large prospective study applied to the Canadian Risk Criteria for Hereditary Renal Cell Carcinoma showed that 35% of patients met at least one genetic testing criterion, with non-clear cell histology criteria accounting for the largest proportion of patients, followed by age ≤45 years, first- or second-degree relative with any renal tumour and bilaterality or multifocality (30). However, previous studies demonstrated low referral of genetic testing in patients who met above-mentioned criteria. Pace A et al. stated that the age of onset (≤46 years) alone could not be an indication for testing (31). It is worth noting that there were still 41.7% of SRP and 75.0% of OCP patients older than 46 years in our study, which means a significant portion of patients predisposed to hereditary RCC would probably be missed if only the age is taken into consideration. Further, previous studies pointed out the average onset age of some syndromic RCCs is over 46, such as BHD syndrome (about 51 years) (32, 33). Hence, it is rational to reset the recommended age and expand the testing population to find out more RCC patients with hereditary susceptibility.

In the past decade, it was realized that phenotype-specific single gene testing might lead to misinterpretation due to insufficient detection of target genes. Multigene panel testing conferred the possibility to parallelly detect more than one PGV in one procedure with relatively low cost. In this study, we observed 1.6% (5/322) of the tested population had more than one deleterious mutation in one individual. A larger study based on 2984 patients with solid tumor demonstrated that 13.3% had germline mutations and 0.9% (27/2984) carried more than one pathogenic germline variant. These results remind us not to ignore the possibility that one patient has multiple germline mutations, although it is relatively rare (34). Additionally, we identified about 6% patients had PGVs in other cancer-associated genes, which is also observed in other studies (10, 16). This finding highlights the applicability of multigene panel testing which facilitated efficient identification of more potentially pathogenic germline alterations for RCC patients.

To analyze clinical characteristics of RCC patients based on germline variant status, the entire cohort was stratified into SRP, OCP and NCP groups. Male-to-female ratio in NCP group was close to 2:1, while it was approximately 1:1 in the other two groups. Nearly half of the NCP patients had smoking history, however approximately one quarter of SRP and OCP patients smoked cigarettes. More patients with advanced-disease were observed in the OCP group. Mutational signatures based on he specific changes of individual nucleotides and their combinations on the genome reveal the diversity and complexity of somatic mutational processes underlying oncogenesis. Next, four mutational signatures (cosine similarity>0.7) were identified among three groups. SBS 5 was identified in SRP, SBS 6 was observed in OCP, SBS1 and SBS 26 were only observed in NCP. Those mutational signatures are associated with different aetiology. For example, SBS 6 and SBS 26 are associated with dMMR and MSI, whereas SBS 1 is probably due to the endogenous mutational process and correlated with age (35). These findings indicate the different consequences of multiple mutational processes in each group which still require further investigation.

The Knudsen’s “two-hit” hypothesis proposes that most tumor suppressor genes require mutation or epigenetic silencing to inactivate both alleles to cause phenotypic presentation (36). Carlo’s study detected somatic variants and LOH in patients with germline variants in different tumor suppressor genes, such as *FH*, *BAP1* and *VHL* (10). In our study, 39.3% (11/28) patients with PGVs were detected to have somatic “second hits” like LOH, large CNV loss and somatic mutations. Although 3p loss was identified in all six patients with *VHL* germline mutation, it seems that the overall LOH frequency was lower than that from Carlo’s report. To be clear, genomic DNA microarray serves as the gold standard method to detect LOH, but algorithms based on SNP backbones to detect LOH may attenuate detection power, due to limitations in the genomic covering by targeted sequencing (37). Promoter hypermethylation was not investigated in our study, which might provide further evidence for “second hits”.

Germline mutation information is instructive and meaningful in the clinical decision-making process. For VHL and BHD syndromes, kidney tumors are usually managed conservatively with active surveillance strategy as primary choice, until the maximal diameter reaches 3 cm, and enucleation or ablation is recommended when surgery is needed (29). However, the 3-cm rule does not apply to aggressive subtypes such as HLRCC (38), and wide-margin partial nephrectomy or radical nephrectomy is preferred for HLRCC patients, even in the early-stage. Obviously, timely identification of the hereditary RCC syndromes through germline testing would be valuable for guiding systemic and surgical treatments. Furthermore, a study conducted in 100 cases in Italy revealed that renal tumor biopsy helps to establish pre-treatment diagnosis, reduces overtreatment, has a low risk of complications and high diagnostic rate (39). Moreover, screening could be initiated at a very early stage even before clinical penetrance if necessary, to benefit other family members. Our results showed that potentially actionable gene variants could be detected in 17.2% of RCC patients based on NCCN guidelines and OncoKB database, which is consistent with results in kidney cancer from a pan-cancer study (40). One example is that NCCN guideline recommends everolimus as a systemic therapy for patient with tuberous sclerosis syndrome, which is often characterized with *TSC1/2* germline mutations. Under the circumstances of refractory disease or progressed on guideline recommended therapies, information of actionable variants uncovered by sequencing might give clues about novel therapies which would possibly benefit selected patients. This kind of “novel therapy” is, in most cases, routinely recommended in other cancer types. For example, prostate or ovarian cancer patients with germline mutations in *BRCA1/2*, which implies DDR deficiency, response well to PARP inhibitors (41). In this study, and also some other reports (10, 16), we also found DDR gene mutations in a portion of RCC patients, which implies a potential role of DDR pathways-targeting therapy in RCC patients, especially after failure of multiple lines of standard treatments. Consequently, germline and somatic mutation screening would be a rational recommendation not only to identify individuals (and their families) with hereditary susceptibility but also to explore potential therapies for patients with advanced disease.

There are some limitations in this study. First, treatment and follow-up information were lacking, and sample size was also limited. Secondly, only SNVs and InDels were interpreted for germline variation analysis following ACMG guidelines, while other variation types were not covered in this study. Finally, only P/LP carriers, rather than VUS (Variant with unknown significance) carriers, were considered when we evaluated the frequency of germline variations, which means the mutation prevalence might be underestimated.

In conclusion, our results from an unselected and unbiased cohort underlined the significance of genetic testing in an expanded RCC population, which is not simply based on identifiable clinical characteristics, age of onset and family history. In addition, germline and somatic sequencing results would provide valuable information for genetic consulting, risk assessment and clinical care decision-making, not only for early-stage, but also for advanced conditions.

## Data Availability Statement

The original contributions presented in the study are publicly available. This data can be found here https://bigd.big.ac.cn/bioproject/browse/PRJCA007218. The datasets presented in this study can be found in online repositories. The names of the repository/repositories and accession number(s) can be found in the article/**Supplementary Material**.

## Ethics Statement

Ethical review and approval was not required for the study on human participants in accordance with the local legislation and institutional requirements. Written informed consent to participate in this study was provided by the participants’ legal guardian/next of kin.

## Author Contributions

WK: data curation, formal analysis, visualization and writing–original draft. TY: formal analysis, visualization and writing–original draft. XW: formal analysis and investigation. ZM: formal analysis and investigation. CZ: formal analysis and investigation. SH: formal analysis and investigation. JT: formal analysis and investigation. XZ: methodology and validation. TZ: methodology and validation. YZ: visualization and formal analysis. FL: supervision and writing–review. SC: supervision and writing–review. HW: Conceptualization, funding acquisition, supervision, resources and writing–review. JZ: Conceptualization, funding acquisition, supervision, resources and writing–review. All authors contributed to the article and approved the submitted version.

## Funding

This study was supported by the Incubating Program for Clinical Research and Innovation of Renji Hospital (PYIII20-10).

## Conflict of Interest

Author TY, XZ, TZ, YZ, FL, SC, and HW are employed by Acornmed Biotechnology Co., Ltd.

The remaining authors declare that the research was conducted in the absence of any commercial or financial relationships that could be construed as a potential conflict of interest

## Publisher’s Note

All claims expressed in this article are solely those of the authors and do not necessarily represent those of their affiliated organizations, or those of the publisher, the editors and the reviewers. Any product that may be evaluated in this article, or claim that may be made by its manufacturer, is not guaranteed or endorsed by the publisher.
