# Supplementary material for: Germline Mutation Landscape and Associated Clinical Characteristics in Chinese Patients With Renal Cell Carcinoma
Source: Front Oncol. 2021 Dec 2;11:737547. doi: 10.3389/fonc.2021.737547 (PMC8675086; doi:10.3389/fonc.2021.737547)
Supplement: Supplementary file 1 [file DataSheet_1.docx]

Supplementary Tables S 1: Genes included in the somatic and germline 808 genes

| **Genes** | **Syndromes associated with cancer** | **Chort** | **DNA damage repair genes** |  | **Genes** | **Syndromes associated with cancer** | **Chort** | **DNA damage repair genes** |
| --- | --- | --- | --- | --- | --- | --- | --- | --- |
| *ABL1* |  |  |  |  | *PRKCI* |  |  |  |
| *ABL2* |  |  |  |  | *PRKD1* |  |  |  |
| *ACVR1* |  |  |  |  | *PRKDC* |  |  | DDR |
| *ACVR1B* |  |  |  |  | *PRSS1* |  |  |  |
| *AGO2* |  |  |  |  | *PRSS8* |  |  |  |
| *AKT1* |  |  |  |  | *PTCH1* | Nevoid basal cell carcinoma syndrome (NBCCS) | OCP |  |
| *AKT2* |  |  |  |  | ***PTEN*** | *PTEN* hamartoma tumor syndrome | OCP | DDR |
| *AKT3* |  |  |  |  | *PTP4A1* |  |  |  |
| *ALK* | Familial neuroblastoma | OCP |  |  | *PTPN11* |  |  |  |
| *ALOX12B* |  |  |  |  | *PTPRD* |  |  |  |
| *AMER1* |  |  |  |  | *PTPRS* |  |  |  |
| *ANKRD11* |  |  |  |  | *PTPRT* |  |  |  |
| *APC* | Familial adenomatous polyposis | OCP |  |  | *QKI* |  |  |  |
| *AR* |  |  |  |  | *RAB35* |  |  |  |
| *ARAF* |  |  |  |  | *RAC1* |  |  |  |
| *ARFRP1* |  |  |  |  | *RAC2* |  |  |  |
| *ARID1A* |  |  |  |  | *RAD21* |  |  |  |
| *ARID1B* |  |  |  |  | *RAD50* | Nijmegen breakage syndrome_x005f like disorder | OCP | DDR |
| *ARID2* |  |  |  |  | *RAD51* | Hereditary breast cancer | OCP | DDR |
| *ARID5B* |  |  |  |  | *RAD51B* | Hereditary breast cancer | OCP | DDR |
| *ASXL1* |  |  |  |  | *RAD51C* | *RAD51C*-related cancer; Fanconi anemia | OCP | DDR |
| *ASXL2* |  |  |  |  | *RAD51D* | Hereditary ovarian cancer | OCP | DDR |
| *ATM* | Ataxia-telangiectasia; ATM-related cancer risk | OCP | DDR |  | *RAD52* |  |  | DDR |
| *ATR* | Cutaneous telangiectasia and cancer syndrome, familial | OCP | DDR |  | *RAD54L* |  |  | DDR |
| *ATRX* |  |  | DDR |  | *RAF1* |  |  |  |
| *AURKA* |  |  |  |  | *RANBP2* |  |  |  |
| *AURKB* |  |  |  |  | *RARA* |  |  |  |
| *AXIN1* |  |  |  |  | *RASA1* |  |  |  |
| *AXIN2* |  |  |  |  | *RB1* | Retinoblastoma | OCP |  |
| *AXL* |  |  |  |  | *RBM10* |  |  |  |
| *B2M* |  |  |  |  | *RECQL* |  |  | DDR |
| *BABAM1* |  |  | DDR |  | *RECQL3* |  |  |  |
| ***BAP1*** | Mesothelioma, uveal melanoma,RCC | SRP |  |  | *RECQL4* | Rothmund-Thomson syndrome(RTS) | OCP | DDR |
| *BARD1* | Hereditary breast and ovarian cancer syndrome | OCP | DDR |  | *REL* |  |  |  |
| *BBC3* |  |  |  |  | *REQ4* |  |  |  |
| *BCL10* |  |  |  |  | *RET* | Multiple endocrine neoplasia, type 2 | OCP |  |
| *BCL2* |  |  |  |  | *RFWD2* |  |  |  |
| *BCL2L1* |  |  |  |  | *RHBDF2* |  |  |  |
| *BCL2L11* |  |  |  |  | *RHEB* |  |  |  |
| *BCL2L2* |  |  |  |  | *RHOA* |  |  |  |
| *BCL6* |  |  |  |  | *RICTOR* |  |  |  |
| *BCOR* |  |  |  |  | *RINT1* |  |  |  |
| *BCORL1* |  |  |  |  | *RIT1* |  |  |  |
| *BCR* |  |  |  |  | *RNF43* |  |  |  |
| *BIRC3* |  |  |  |  | *ROS1* |  |  |  |
| *BLM* | Bloom syndrome | OCP | DDR |  | *RPL11* |  |  |  |
| *BMPR1A* | Juvenile polyposis syndrome | OCP |  |  | *RPL35A* |  |  |  |
| *BRAF* |  |  |  |  | *RPL5* |  |  |  |
| ***BRCA1*** | Hereditary breast and ovarian cancer syndrome | OCP | DDR |  | *RPS10* |  |  |  |
| ***BRCA2*** | Hereditary breast and ovarian cancer syndrome, Fanconi anemia | OCP | DDR |  | *RPS17* |  |  |  |
| *BRD4* |  |  |  |  | *RPS19* |  |  |  |
| *BRIP1* | BRIP1-related cancer; Fanconi anemia | OCP | DDR |  | *RPS24* |  |  |  |
| *BTG1* |  |  |  |  | *RPS26* |  |  |  |
| *BTK* |  |  |  |  | *RPS6KA4* |  |  |  |
| *C11orf30* |  |  |  |  | *RPS6KB2* |  |  |  |
| *C17orf39* |  |  |  |  | *RPS7* |  |  |  |
| *CALR* |  |  |  |  | *RPTOR* |  |  |  |
| *CARD11* |  |  |  |  | *RRAGC* |  |  |  |
| *CARM1* |  |  |  |  | *RRAS* |  |  |  |
| *CASP8* |  |  |  |  | *RRAS2* |  |  |  |
| *CBFB* |  |  |  |  | *RTEL1* |  |  | DDR |
| *CBL* |  |  |  |  | *RUNX1* | Familial platelet disorder with predisposition to acute myelogenous leukaemia | OCP |  |
| *CCND1* |  |  |  |  | *RUNX1T1* |  |  |  |
| *CCND2* |  |  |  |  | *RXRA* |  |  |  |
| *CCND3* |  |  |  |  | *RYBP* |  |  |  |
| *CCNE1* |  |  |  |  | *SDHA* | Hereditary paraganglioma-pheochromocytoma (PGL/PCC)  syndromes | SRP |  |
| *CD274* |  |  |  |  | *SDHAF2* | Hereditary paraganglioma-pheochromocytoma (PGL/PCC)  syndromes | OCP |  |
| *CD276* |  |  |  |  | ***SDHB*** | Hereditary paraganglioma-pheochromocytoma (PGL/PCC)  syndromes | SRP |  |
| *CD79A* |  |  |  |  | ***SDHC*** | Hereditary paraganglioma-pheochromocytoma (PGL/PCC)  syndromes | SRP |  |
| *CD79B* |  |  |  |  | ***SDHD*** | Hereditary paraganglioma-pheochromocytoma (PGL/PCC)  syndromes | SRP |  |
| *CDC42* |  |  |  |  | *SESN1* |  |  |  |
| *CDC73* |  |  |  |  | *SESN2* |  |  |  |
| *CDH1* | Hereditary diffuse gastric cancer | OCP |  |  | *SESN3* |  |  |  |
| *CDK12* |  |  |  |  | ***SETD2*** |  |  |  |
| *CDK4* | Familial cutaneous melanoma | OCP |  |  | *SF3B1* |  |  |  |
| *CDK6* |  |  |  |  | *SH2B3* |  |  |  |
| *CDK8* |  |  |  |  | *SH2D1A* |  |  |  |
| *CDKN1A* |  |  |  |  | *SHOC2* |  |  |  |
| *CDKN1B* |  |  |  |  | *SHQ1* |  |  |  |
| ***CDKN2A*** |  |  |  |  | *SLIT2* |  |  |  |
| *CDKN2B* |  |  |  |  | *SLX4* | Fanconi anemia, complementation group P | OCP | DDR |
| *CDKN2C* |  |  |  |  | *SMAD2* |  |  |  |
| *CEBPA* |  |  |  |  | *SMAD3* | Thoracic aortic aneurysms and aortic dissections (TAAD) | OCP |  |
| *CENPA* |  |  |  |  | *SMAD4* | Juvenile polyposis syndrome | OCP |  |
| *CFTR* |  |  |  |  | *SMARCA4* | Rhabdoid tumor predisposition syndrome type 2 | OCP | DDR |
| *CHD2* |  |  |  |  | *SMARCB1* | Rhabdoid tumor predisposition syndrome type 1 | OCP |  |
| *CHD4* |  |  |  |  | *SMARCD1* |  |  |  |
| *CHEK1* |  |  | DDR |  | *SMO* |  |  |  |
| *CHEK2* | *CHEK2*-related cancer | OCP | DDR |  | *SMYD3* |  |  |  |
| *CIC* |  |  |  |  | *SNCAIP* |  |  |  |
| *CREBBP* |  |  |  |  | *SOCS1* |  |  |  |
| *CRKL* |  |  |  |  | *SOS1* |  |  |  |
| *CRLF2* |  |  |  |  | *SOX10* |  |  |  |
| *CSDE1* |  |  |  |  | *SOX17* |  |  |  |
| *CSF1R* |  |  |  |  | *SOX2* |  |  |  |
| *CSF3R* |  |  |  |  | *SOX9* |  |  |  |
| *CTCF* |  |  |  |  | *SPEN* |  |  |  |
| *CTLA4* |  |  |  |  | *SPINK1* |  |  |  |
| *CTNNA1* |  |  |  |  | *SPOP* |  |  |  |
| *CTNNB1* |  |  |  |  | *SPRED1* |  |  |  |
| *CUL3* |  |  | DDR |  | *SPTA1* |  |  |  |
| *CXCR4* |  |  |  |  | *SRC* |  |  |  |
| *CYLD* |  |  |  |  | *SRSF2* |  |  |  |
| *CYP2D6* |  |  |  |  | *STAG2* |  |  |  |
| *CYSLTR2* |  |  |  |  | *STAT3* |  |  |  |
| *DAXX* |  |  |  |  | *STAT4* |  |  |  |
| *DCUN1D1* |  |  |  |  | *STAT5A* |  |  |  |
| *DDR2* |  |  |  |  | *STAT5B* |  |  |  |
| *DICER1* | *DICER1*-related disorders | OCP |  |  | *STK11* | Peutz-Jeghers syndrome | OCP |  |
| *DIS3* |  |  |  |  | *STK19* |  |  |  |
| *DNAJB1* |  |  |  |  | *STK40* |  |  |  |
| *DNMT1* |  |  |  |  | *SUFU* | Medulloblastoma | OCP |  |
| *DNMT3A* |  |  |  |  | *SUZ12* |  |  |  |
| *DNMT3B* |  |  |  |  | *SYK* |  |  |  |
| *DOT1L* |  |  |  |  | *TAF1* |  |  |  |
| *DROSHA* |  |  |  |  | *TAP1* |  |  |  |
| *DUSP4* |  |  |  |  | *TAP2* |  |  |  |
| *E2F3* |  |  |  |  | *TBX3* |  |  |  |
| *EED* |  |  |  |  | ***TCEB1*** |  |  | DDR |
| *EGFL7* |  |  |  |  | *TCF3* |  |  |  |
| *EGFR* | Familial lung cancer | OCP |  |  | *TCF7L2* |  |  |  |
| *EIF1AX* |  |  |  |  | *TEK* |  |  |  |
| *EIF4A2* |  |  |  |  | *TERC* |  |  |  |
| *EIF4E* |  |  |  |  | *TERT* | Familial pulmonary fibrosis (FPF); Dyskeratosis congenita(DC) | OCP |  |
| *ELF3* |  |  |  |  | *TET1* |  |  |  |
| *EMSY* |  |  |  |  | *TET2* |  |  |  |
| *ENG* |  |  |  |  | *TGFBR1* | Thoracic aortic aneurysms and aortic dissections (TAAD) | OCP |  |
| *EP300* |  |  |  |  | *TGFBR2* | Thoracic aortic aneurysms and aortic dissections (TAAD) | OCP |  |
| *EPAS1* |  |  |  |  | *TMEM127* | Familial pheochromocytoma syndrome | OCP |  |
| *EPCAM* | Lynch syndrome | OCP |  |  | *TMPRSS2* |  |  |  |
| *EPHA3* |  |  |  |  | *TNFAIP3* |  |  |  |
| *EPHA5* |  |  |  |  | *TNFRSF14* |  |  |  |
| *EPHA7* |  |  |  |  | *TOP1* |  |  |  |
| *EPHB1* |  |  |  |  | *TOP2A* |  |  |  |
| *ERBB2* |  |  |  |  | ***TP53*** | Li-Fraumeni syndrome | OCP | DDR |
| *ERBB3* |  |  |  |  | *TP53BP1* |  |  | DDR |
| *ERBB4* |  |  |  |  | *TP63* |  |  |  |
| *ERCC2* |  |  | DDR |  | *TRAF2* |  |  |  |
| *ERCC3* |  |  | DDR |  | *TRAF7* |  |  |  |
| *ERCC4* |  |  | DDR |  | ***TSC1*** | Tuberous sclerosis complex(TSC) | SRP |  |
| *ERCC5* |  |  | DDR |  | ***TSC2*** | Tuberous sclerosis complex(TSC) | SRP |  |
| *ERF* |  |  |  |  | *TSHR* | Thyroid carcinoma with thyrotoxicosis | OCP |  |
| *ERG* |  |  |  |  | *U2AF1* |  |  |  |
| *ERRFI1* |  |  |  |  | *UPF1* |  |  |  |
| *ESR1* |  |  |  |  | *VEGFA* |  |  |  |
| *ETV1* |  |  |  |  | ***VHL*** | Von Hippel-Lindau syndrome;Familial erythrocytosis, type 2 | SRP |  |
| *ETV4* |  |  |  |  | *VTCN1* |  |  |  |
| *ETV5* |  |  |  |  | *WHSC1* |  |  |  |
| *ETV6* |  |  |  |  | *WHSC1L1* |  |  |  |
| *EWSR1* |  |  |  |  | *WISP3* |  |  |  |
| *EZH1* |  |  |  |  | *WRN* |  |  | DDR |
| *EZH2* |  |  |  |  | *WT1* | WAGR (Wilms tumor-aniridia_x005f genital anomalies-retardation)syndrome, Denys-Drash syndrome (DDS), Frasier syndrome, and isolated Wilms tumor | OCP |  |
| *FAM123B* |  |  |  |  | *WWTR1* |  |  |  |
| *FAM175A* | Hereditary breast cancer syndrome | OCP | DDR |  | *XIAP* |  |  |  |
| *FAM46C* |  |  |  |  | *XPO1* |  |  |  |
| *FAM58A* |  |  |  |  | *XRCC2* |  |  | DDR |
| *FANCA* |  |  | DDR |  | *YAP1* |  |  |  |
| *FANCB* |  |  | DDR |  | *YES1* |  |  |  |
| *FANCC* |  |  | DDR |  | *ZBTB2* |  |  |  |
| *FANCD1* |  |  |  |  | *ZFHX3* |  |  |  |
| *FANCD2* |  |  | DDR |  | *ZNF217* |  |  |  |
| *FANCE* |  |  | DDR |  | *ZNF703* |  |  |  |
| *FANCF* |  |  | DDR |  | *ZRSR2* |  |  |  |
| *FANCG* |  |  | DDR |  | *APCDD1* |  |  |  |
| *FANCL* |  |  | DDR |  | *BACH1* |  |  |  |
| *FAS* |  |  |  |  | *CHUK* |  |  |  |
| *FAT1* |  |  |  |  | *CRBN* |  |  |  |
| *FBXW7* |  |  |  |  | *CUL4A* |  |  | DDR |
| *FGF10* |  |  |  |  | *CUL4B* |  |  |  |
| *FGF14* |  |  |  |  | *CYP17A1* |  |  |  |
| *FGF19* |  |  |  |  | *FANCI* |  |  | DDR |
| *FGF23* |  |  |  |  | *FANCM* |  |  | DDR |
| *FGF3* |  |  |  |  | *FAT3* |  |  |  |
| *FGF4* |  |  |  |  | *FGF12* |  |  |  |
| *FGF6* |  |  |  |  | *FGF7* |  |  |  |
| *FGFR1* |  |  |  |  | *PAK7* |  |  |  |
| *FGFR2* |  |  |  |  | *PARP2* |  |  | DDR |
| *FGFR3* |  |  |  |  | *PARP3* |  |  | DDR |
| *FGFR4* |  |  |  |  | *PARP4* |  |  | DDR |
| ***FH*** | Hereditary Leiomyomatosis and Renal Cell Cancer | SRP |  |  | *RPA1* |  |  | DDR |
| ***FLCN*** | *Birt-Hogg-Dubé* syndrome | SRP |  |  | *TIPARP* |  |  |  |
| *FLT1* |  |  |  |  | *TRRAP* |  |  |  |
| *FLT3* |  |  |  |  | *XRCC3* |  |  | DDR |
| *FLT4* |  |  |  |  | *GRIK3* |  |  |  |
| *FOXA1* |  |  |  |  | *LRRIQ3* |  |  |  |
| *FOXL2* |  |  |  |  | *ERICH3* |  |  |  |
| *FOXO1* |  |  |  |  | *TGFBR3* |  |  |  |
| *FOXP1* |  |  |  |  | *LPPR4* |  |  |  |
| *FRS2* |  |  |  |  | *DUSP27* |  |  |  |
| *FUBP1* |  |  |  |  | *TNN* |  |  |  |
| *FYN* |  |  |  |  | *TNR* |  |  |  |
| *GABRA6* |  |  |  |  | *PAPPA2* |  |  |  |
| *GALNT12* |  |  |  |  | *ASTN1* |  |  |  |
| *GATA1* |  |  |  |  | *BRINP3* |  |  |  |
| *GATA2* | Familial MDS-AML | OCP |  |  | *KCNT2* |  |  |  |
| *GATA3* |  |  |  |  | *RYR2* |  |  |  |
| *GATA4* |  |  |  |  | *NLRP3* |  |  |  |
| *GATA6* |  |  |  |  | *TRIM58* |  |  |  |
| *GEN1* |  |  | DDR |  | *GPR158* |  |  |  |
| *GID4* |  |  |  |  | *GRID1* |  |  |  |
| *GLI1* |  |  |  |  | *KCNA4* |  |  |  |
| *GNA11* |  |  |  |  | *LRRC4C* |  |  |  |
| *GNA13* |  |  |  |  | *MS4A3* |  |  |  |
| *GNAQ* |  |  |  |  | *NCAM1* |  |  |  |
| *GNAS* |  |  |  |  | *NTM* |  |  |  |
| *GPR124* |  |  |  |  | *ABCC9* |  |  |  |
| *GPS2* |  |  |  |  | *NAV3* |  |  |  |
| *GREM1* | Hereditary mixed polyposis syndrome (HMPS) | OCP |  |  | *PCDH17* |  |  |  |
| *GRIN2A* |  |  |  |  | *KLHL1* |  |  |  |
| *GRM3* |  |  |  |  | *SLITRK1* |  |  |  |
| *GSK3B* |  |  |  |  | *LRFN5* |  |  |  |
| *H3F3A* |  |  |  |  | *C14orf177* |  |  |  |
| *H3F3AP4* |  |  |  |  | *MKRN3* |  |  |  |
| *H3F3B* |  |  |  |  | *ZNF423* |  |  |  |
| *H3F3C* |  |  |  |  | *SALL1* |  |  |  |
| *HFE* |  |  |  |  | *POLDIP2* |  |  |  |
| *HGF* |  |  |  |  | *MIR4728* |  |  |  |
| *HIF2A* |  |  |  |  | *CA10* |  |  |  |
| *HIST1H1C* |  |  |  |  | *KIF2B* |  |  |  |
| *HIST1H2BD* |  |  |  |  | *ZNF521* |  |  |  |
| *HIST1H3A* |  |  |  |  | *ASXL3* |  |  |  |
| *HIST1H3B* |  |  |  |  | *SETBP1* |  |  |  |
| *HIST1H3C* |  |  |  |  | *SLC14A2* |  |  |  |
| *HIST1H3D* |  |  |  |  | *ZNF536* |  |  |  |
| *HIST1H3E* |  |  |  |  | *TSHZ3* |  |  |  |
| *HIST1H3F* |  |  |  |  | *IGFL3* |  |  |  |
| *HIST1H3G* |  |  |  |  | *NLRP5* |  |  |  |
| *HIST1H3H* |  |  |  |  | *ZIM2* |  |  |  |
| *HIST1H3I* |  |  |  |  | *APOB* |  |  |  |
| *HIST1H3J* |  |  |  |  | *SLC8A1* |  |  |  |
| *HIST2H3A* |  |  |  |  | *LRRTM4* |  |  |  |
| *HIST2H3C* |  |  |  |  | *REG3A* |  |  |  |
| *HIST2H3D* |  |  |  |  | *CTNNA2* |  |  |  |
| *HIST3H3* |  |  |  |  | *ST6GAL2* |  |  |  |
| *HLA-A* |  |  |  |  | *CNTNAP5* |  |  |  |
| *HLA-B* |  |  |  |  | *KCNJ3* |  |  |  |
| *HNF1A* |  |  |  |  | *XIRP2* |  |  |  |
| *HOXB13* |  |  |  |  | *ZNF804A* |  |  |  |
| *HRAS* | Costello syndrome | OCP |  |  | *SPHKAP* |  |  |  |
| *HSD3B1* |  |  |  |  | *ASB18* |  |  |  |
| *HSP90AA1* |  |  |  |  | *PDYN* |  |  |  |
| *ICOSLG* |  |  |  |  | *ZNF831* |  |  |  |
| *ID3* |  |  |  |  | *PDZRN3* |  |  |  |
| *IDH1* |  |  | DDR |  | *ZIC4* |  |  |  |
| *IDH2* |  |  |  |  | *ZIC1* |  |  |  |
| *IFNGR1* |  |  |  |  | *IQCJ* |  |  |  |
| *IGF1* |  |  |  |  | *SLITRK3* |  |  |  |
| *IGF1R* |  |  |  |  | *GABRA2* |  |  |  |
| *IGF2* |  |  |  |  | *FRYL* |  |  |  |
| *IKBKE* |  |  |  |  | *PDHA2* |  |  |  |
| *IKZF1* |  |  |  |  | *ANK2* |  |  |  |
| *IL10* |  |  |  |  | *PCDH10* |  |  |  |
| *IL7R* |  |  |  |  | *FBXL7* |  |  |  |
| *INHA* |  |  |  |  | *CDH18* |  |  |  |
| *INHBA* |  |  |  |  | *CDH12* |  |  |  |
| *INPP4A* |  |  |  |  | *CDH10* |  |  |  |
| *INPP4B* |  |  |  |  | *CDH9* |  |  |  |
| *INPPL1* |  |  |  |  | *ADAMTS12* |  |  |  |
| *INSR* |  |  |  |  | *SLC45A2* |  |  |  |
| *IRF2* |  |  |  |  | *HCN1* |  |  |  |
| *IRF4* |  |  |  |  | *HTR1A* |  |  |  |
| *IRS1* |  |  |  |  | *HAPLN1* |  |  |  |
| *IRS2* |  |  |  |  | *FBN2* |  |  |  |
| *JAK1* |  |  |  |  | *SLIT3* |  |  |  |
| *JAK2* | Familial thrombocytosis | OCP |  |  | *PRIM2* |  |  |  |
| *JAK3* |  |  |  |  | *C6orf118* |  |  |  |
| *JUN* |  |  |  |  | *HDAC9* |  |  |  |
| *KAT6A* |  |  |  |  | *POM121L12* |  |  |  |
| *KDM5A* |  |  |  |  | *ZNF804B* |  |  |  |
| ***KDM5C*** |  |  |  |  | *ZAN* |  |  |  |
| ***KDM6A*** |  |  |  |  | *KCND2* |  |  |  |
| *KDR* |  |  |  |  | *GRM8* |  |  |  |
| *KEAP1* |  |  |  |  | *LOC349160* |  |  |  |
| *KEL* |  |  |  |  | *CNTNAP2* |  |  |  |
| *KIT* | Hereditary Gastrointestinal stromal tumors (GISTs) | OCP |  |  | *RP1L1* |  |  |  |
| *KLF4* |  |  |  |  | *LPL* |  |  |  |
| *KLHL6* |  |  |  |  | *PXDNL* |  |  |  |
| *KMT2A* |  |  |  |  | *ZFHX4* |  |  |  |
| *KMT2B* |  |  |  |  | *DCAF4L2* |  |  |  |
| *KMT2C* |  |  |  |  | *CSMD3* |  |  |  |
| *KMT2D* |  |  |  |  | *TG* |  |  |  |
| *KMT5A* |  |  |  |  | *FAM135B* |  |  |  |
| *KNSTRN* |  |  |  |  | *COL22A1* |  |  |  |
| *KRAS* | Noonan Syndrome | OCP |  |  | *ASTN2* |  |  |  |
| *LATS1* |  |  |  |  | *TLR4* |  |  |  |
| *LATS2* |  |  |  |  | *DMD* |  |  |  |
| *LMO1* |  |  |  |  | *FGD1* |  |  |  |
| *LRP1B* |  |  |  |  | *TRPC5* |  |  |  |
| *LYN* |  |  |  |  | *AMOT* |  |  |  |
| *LZTR1* |  |  |  |  | *DCAF12L2* |  |  |  |
| *MAGI2* |  |  |  |  | *DCAF12L1* |  |  |  |
| *MALT1* |  |  |  |  | *ADGRG4* |  |  |  |
| *MAP2K1* |  |  |  |  | *SLITRK2* |  |  |  |
| *MAP2K2* |  |  |  |  | *MARCH1* |  |  |  |
| *MAP2K4* |  |  |  |  | *ABCB1* |  |  |  |
| *MAP3K1* |  |  |  |  | *ATIC* |  |  |  |
| *MAP3K13* |  |  |  |  | *C2orf44* |  |  |  |
| *MAP3K14* |  |  |  |  | *C8orf34* |  |  |  |
| *MAPK1* |  |  |  |  | *CARS* |  |  |  |
| *MAPK3* |  |  |  |  | *CASP7* |  |  |  |
| *MAPKAP1* |  |  |  |  | *CCDC6* |  |  |  |
| *MAX* | Hereditary paraganglioma_pheochromocytoma (PGL/PCC) syndromes | OCP |  |  | *CD74* |  |  |  |
| *MCL1* |  |  |  |  | *CDA* |  |  |  |
| *MDC1* |  |  | DDR |  | *CDK5RAP2* |  |  |  |
| *MDH2* |  |  |  |  | *CHST3* |  |  |  |
| *MDM2* |  |  |  |  | *CLIP1* |  |  |  |
| *MDM4* |  |  |  |  | *CLTC* |  |  |  |
| *MED12* |  |  |  |  | *CYP19A1* |  |  |  |
| *MEF2B* |  |  |  |  | *CYP1A1* |  |  |  |
| *MEF2BNB-MEF2B* |  |  |  |  | *CYP1B1* |  |  |  |
| *MEN1* | Multiple endocrine neoplasia, type 1 | OCP |  |  | *CYP2C8* |  |  |  |
| ***MET*** | Hereditary papillary renal carcinoma | SRP |  |  | *CYP2E1* |  |  |  |
| *MGA* |  |  |  |  | *CYP3A4* |  |  |  |
| ***MITF*** | Familial melanoma and renal cell carcinoma | OCP |  |  | *CYP3A5* |  |  |  |
| *MLH1* | Lynch syndrome | OCP | DDR |  | *CYP4B1* |  |  |  |
| *MPL* |  |  |  |  | *DCK* |  |  |  |
| *MRE11A* | Ataxia-telangiectasia-like disorder (recessive); breast cancer | OCP | DDR |  | *DCTN1* |  |  |  |
| *MSH2* | Lynch syndrome | OCP | DDR |  | *DPYD* |  |  |  |
| *MSH3* |  |  | DDR |  | *DYNC2H1* |  |  |  |
| *MSH6* | Lynch syndrome | OCP | DDR |  | *EGF* |  |  |  |
| *MSI1* |  |  |  |  | *EML4* |  |  |  |
| *MSI2* |  |  |  |  | *EPHA2* |  |  |  |
| *MST1* |  |  |  |  | *ERC1* |  |  |  |
| *MST1R* |  |  |  |  | *ERCC1* |  |  | DDR |
| *MTOR* |  |  |  |  | *EZR* |  |  |  |
| *MUTYH* | *MUTYH*-associated polyposis (MAP) | OCP | DDR |  | *F3* |  |  |  |
| *MYB* |  |  |  |  | *FDPS* |  |  |  |
| *MYC* |  |  |  |  | *FES* |  |  |  |
| *MYCL* |  |  |  |  | *FGFR1OP* |  |  |  |
| *MYCL1* |  |  |  |  | *FIP1L1* |  |  |  |
| *MYCN* |  |  |  |  | *FN1* |  |  |  |
| *MYD88* |  |  |  |  | *FRK* |  |  |  |
| *MYOD1* |  |  |  |  | *GALNT14* |  |  |  |
| *MYST3* |  |  |  |  | *GOLGA5* |  |  |  |
| *NBN* | Nijmegen breakage syndrome; *NBN*-related cancer risk | OCP | DDR |  | *GOPC* |  |  |  |
| *NCOA3* |  |  |  |  | *GSTA1* |  |  |  |
| *NCOR1* |  |  |  |  | *GSTP1* |  |  |  |
| *NEGR1* |  |  |  |  | *HIP1* |  |  |  |
| *NF1* | Neurofibromatosis, type 1 | OCP |  |  | *HOOK3* |  |  |  |
| ***NF2*** | Neurofibromatosis, type 2 | OCP |  |  | *IFNL3* |  |  |  |
| *NFE2L2* |  |  |  |  | *IFNL4* |  |  |  |
| *NFKBIA* |  |  |  |  | *IMPDH2* |  |  |  |
| *NKX2-1* |  |  |  |  | *KIAA1598* |  |  |  |
| *NKX3-1* |  |  |  |  | *KIF5B* |  |  |  |
| *NOTCH1* |  |  |  |  | *KLC1* |  |  |  |
| *NOTCH2* |  |  |  |  | *KTN1* |  |  |  |
| *NOTCH3* | Myofibromatosis | OCP |  |  | *LRIG3* |  |  |  |
| *NOTCH4* |  |  |  |  | *LRRK2* |  |  |  |
| *NPM1* |  |  |  |  | *LTK* |  |  |  |
| *NRAS* | Autoimmune lymphoproliferative syndrome (ALPS) | OCP |  |  | *MAP3K5* |  |  |  |
| *NSD1* |  |  |  |  | *MLL3* |  |  |  |
| *NTHL1* |  |  | DDR |  | *MSN* |  |  |  |
| *NTRK1* |  |  |  |  | *MTHFR* |  |  |  |
| *NTRK2* |  |  |  |  | *MTR* |  |  |  |
| *NTRK3* |  |  |  |  | *MTRR* |  |  |  |
| *NUF2* |  |  |  |  | *MYO5A* |  |  |  |
| *NUP93* |  |  |  |  | *NAT2* |  |  |  |
| *NUTM1* |  |  |  |  | *NCOA4* |  |  |  |
| *PAK1* |  |  |  |  | *NOS3* |  |  |  |
| *PAK3* |  |  |  |  | *NQO1* |  |  |  |
| *PAK6* |  |  |  |  | *NUDT15* |  |  | DDR |
| *PALB2* | *PALB2*-related cancer; Fanconi anemia | OCP | DDR |  | *PAK5* |  |  |  |
| *PALLD* |  |  |  |  | *PCM1* |  |  |  |
| *PARK2* |  |  |  |  | *PML* |  |  |  |
| *PARP1* |  |  | DDR |  | *PPARD* |  |  |  |
| *PAX5* | B cell precursor acute lymphoblastic leukemia (B-ALL) | OCP |  |  | *PPFIBP1* |  |  |  |
| *PAX8* |  |  |  |  | *PRKACA* |  |  |  |
| ***PBRM1*** |  |  |  |  | *PWWP2A* |  |  |  |
| *PDCD1* |  |  |  |  | *ROCK1* |  |  |  |
| *PDCD1LG2* |  |  |  |  | *RPL13* |  |  |  |
| *PDGFRA* | Hereditary Gastrointestinal stromal tumors (GISTs) | OCP |  |  | *RRM1* |  |  | DDR |
| *PDGFRB* |  |  |  |  | *RRM2* |  |  | DDR |
| *PDK1* |  |  |  |  | *RRM2B* |  |  | DDR |
| *PDPK1* |  |  |  |  | *SDC4* |  |  |  |
| *PGR* |  |  |  |  | *SEC31A* |  |  |  |
| *PHOX2B* | Familial neuroblastoma; Congenital central hypoventilation syndrome (CCHS) | OCP |  |  | *SEMA3C* |  |  |  |
| *PIK3C2B* |  |  |  |  | *SLC19A1* |  |  |  |
| *PIK3C2G* |  |  |  |  | *SLC22A12* |  |  |  |
| *PIK3C3* |  |  |  |  | *SLC34A2* |  |  |  |
| *PIK3CA* |  |  |  |  | *SLCO1B1* |  |  |  |
| *PIK3CB* |  |  |  |  | *SLCO1B3* |  |  |  |
| *PIK3CD* |  |  |  |  | *SOD2* |  |  |  |
| *PIK3CG* |  |  |  |  | *SPG7* |  |  |  |
| *PIK3R1* |  |  |  |  | *SQSTM1* |  |  |  |
| *PIK3R2* |  |  |  |  | *STRN* |  |  |  |
| *PIK3R3* |  |  |  |  | *SULT2B1* |  |  |  |
| *PIM1* |  |  |  |  | *TEKT4* |  |  |  |
| *PLCG2* |  |  |  |  | *TFG* |  |  |  |
| *PLK2* |  |  |  |  | *TPM3* |  |  |  |
| *PMAIP1* |  |  |  |  | *TPM4* |  |  |  |
| *PMS1* |  |  | DDR |  | *TPMT* |  |  |  |
| *PMS2* |  |  | DDR |  | *TRIM24* |  |  |  |
| *PNRC1* |  |  |  |  | *TRIM27* |  |  |  |
| *POLD1* |  |  | DDR |  | *TRIM33* |  |  |  |
| *POLE* |  |  | DDR |  | *TUBB1* |  |  |  |
| *PPARG* |  |  |  |  | *TYK2* |  |  |  |
| *PPM1D* | Breast cancer | OCP |  |  | *TYMS* |  |  | DDR |
| *PPP2R1A* |  |  |  |  | *UGT1A1* |  |  |  |
| *PPP4R2* |  |  | DDR |  | *UGT1A8* |  |  |  |
| *PPP6C* |  |  |  |  | *UMPS* |  |  |  |
| *PRDM1* |  |  |  |  | *VCL* |  |  |  |
| *PRDM14* |  |  |  |  | *XPC* |  |  | DDR |
| *PREX2* |  |  |  |  | *XRCC1* |  |  | DDR |
| *PRKAR1A* |  |  |  |  | *ZCCHC8* |  |  |  |

Bold genes were represented shared with the Nguyen's Study, underlined genes were represented shared with the Wu's Study.

Supplementary Tables S2. Details of pathogenic/likely pathogenic germline variants detected via next-generation sequencing

| Sample | Gene | Transcript identifier (RefSeq) | Variant | Amino acid change | Variant Consequence | rsID | gnomAD EAS/ExAC EAS allele frequency | SIFT/PolyPhen/CADD | ACMG | Second hit event | DDR | Age | Cohort |
| --- | --- | --- | --- | --- | --- | --- | --- | --- | --- | --- | --- | --- | --- |
| P1 | *VHL* | NM_000551.3 | c.256C>T | p.P86S | missense variant | rs398123481 | 0;0 | T;D;31 | Likely Pathogenic | 3p loss | No | 32 | SRP |
| P2 | *VHL* | NM_000551.3 | c.499C>T | p.R126W | missense variant | rs5030820 | 0;0 | D;D;19.54 | Pathogenic | 3p loss | No | 33 | SRP |
| P3 | *VHL* | NM_000551.3 | c.509T>G | p.V170G | missense variant | Novel | 0;0 | D;P;18.49 | Likely Pathogenic | 3p loss | No | 37 | SRP |
| P4 | *VHL* | NM_000551.3 | c.525C>G | p.Y175* | stop gain | rs5030835 | 0;0 | T;.;20.4 | Pathogenic | undetermined | No | 37 | SRP |
| P5 | *VHL* | NM_000551.3 | c.210_232del | p.P71Sfs*53 | frameshift variant | Novel | 0;0 | -;-;0 | Likely Pathogenic | 3p loss | No | 38 | SRP |
|  | *TP53* | NM_001126113.2 | c.517G>A | p.V173M | missense variant | rs876660754 | 0;0 | D;D;23.4 | Pathogenic | WT | No |  |  |
| P6 | *VHL* | NM_000551.3 | c.333C>G | p.S111R | missense variant | rs765978945 | 0;0 | D;D;28.4 | Likely Pathogenic | 3p loss and Somatic mutation | No | 54 | SRP |
|  | *PBRM1* | NM_018313.4 | c.789del | p.N263Kfs*20 | frameshift variant | Novel | 0;0 | -;-;0 | Likely Pathogenic | 3p loss | No |  |  |
| P7 | *VHL* | NM_000551.3 | c.464-1G>T | p.X133_splice | splice acceptor variant | rs5030817 | 0;0 | .;.;12.39 | Likely Pathogenic | 3p loss | No | 53 | SRP |
|  | *BAP1* | NM_004656.2 | c.382G>T | p.G128* | stop gain | Novel | 0;0 | D;.;40 | Likely Pathogenic | WT | No |  |  |
| P8 | *FH* | NM_000143.3 | c.442C>T | p.Q148* | stop gain | rs1558400571 | 0;0 | D;.;36 | Likely Pathogenic | undetermined | No | 43 | SRP |
| P9 | *FH* | NM_000143.3 | c.220A>T | p.R74* | stop gain | Novel | 0;0 | T;.;34 | Likely Pathogenic | WT | No | 48 | SRP |
| P10 | *TSC2* | NM_000548.3 | c.1288A>T | p.R430* | stop gain | Novel | 0;0 | T;.;32 | Likely Pathogenic | WT | No | 38 | SRP |
|  | *TSC2* | NM_000548.3 | c.1947-2A>T | p.X649_splice | splice acceptor variant | rs5030817 | 0;0 | .;.;10.54 | Likely Pathogenic | WT | No |  |  |
| P11 | *TSC1* | NM_000368.4 | c.988_989del | p.L330Efs*10 | frameshift variant | rs118203477 | 0;0 | -;-;0 | Likely Pathogenic | Somatic mutation | No | 52 | SRP |
| P12 | *FLCN* | NM_144997.5 | c.1579_1580insA | p.R527Qfs*75 | frameshift variant | rs753009073 | 0.0001;0.0001 | -;-;0 | Likely Pathogenic | 17p loss | No | 58 | SRP |
| P13 | *CHEK2* | NM_007194.3 | c.920del | p.G307Efs*13 | frameshift variant | rs786203053 | 0;0 | -;-;0 | Likely Pathogenic | WT | DDR | 70 | OCP |
| P14 | *ATM* | NM_000051.3 | c.7660del | p.H2554Ifs*10 | frameshift variant | Novel | 0;0 | -;-;0 | Likely Pathogenic | WT | DDR | 82 | OCP |
| P15 | *ATM* | NM_000051.3 | c.8494C>T | p.R2832C | missense variant | rs587779872 | 0;0 | D;D;23.2 | Likely Pathogenic | WT | DDR | 72 | OCP |
| P16 | *BLM* | NM_000057.2 | c.3678C>A | p.C1226* | stop gain | Novel | 0;0 | T;.;43 | Likely Pathogenic | WT | DDR | 73 | OCP |
| P17 | *BLM* | NM_000057.2 | c.793G>T | p.E265* | stop gain | rs751718191 | 0;0 | T;.;35 | Likely Pathogenic | undetermined | DDR | 66 | OCP |
|  | *MRE11* | NM_005591.3 | c.1090C>T | p.R364* | stop gain | rs371077728 | 0;0 | T;.;40 | Likely Pathogenic | undetermined | DDR |  |  |
| P18 | *BRCA1* | NM_007294.3 | c.5467+2T>C | p.X1822_splice | splice donor variant | rs5030817 | 0;0 | .;.;16.59 | Likely Pathogenic | WT | DDR | 36 | OCP |
| P19 | *BRCA1* | NM_007294.3 | c.76A>G | p.I26V | missense variant | Novel | 0;0 | T;D;14.5 | Likely Pathogenic | WT | DDR | 41 | OCP |
| P20 | *NBN* | NM_002485.4 | c.877del | p.I293* | frameshift variant | Novel | 0;0 | -;-;0 | Likely Pathogenic | WT | DDR | 57 | OCP |
| P21 | *NBN* | NM_002485.4 | c.591C>G | p.Y197* | stop gain | Novel | 0;0 | T;.;28.7 | Likely Pathogenic | WT | DDR | 59 | OCP |
| P22 | *ATR* | NM_001184.3 | c.4234C>T | p.R1412* | stop gain | rs769216993 | 0;0 | T;.;44 | Likely Pathogenic | WT | DDR | 64 | OCP |
| P23 | *BRCA2* | NM_000059.3 | c.2259del | p.Q754Nfs*18 | frameshift variant | rs397507621 | 0;0 | -;-;0 | Likely Pathogenic | 13 q loss | DDR | 68 | OCP |
| P24 | *FANCA* | NM_000135.2 | c.2299C>T | p.Q767* | stop gain | Novel | 0;0 | D;.;20.4 | Likely Pathogenic | *FANCA* LOH | DDR | 62 | OCP |
| P25 | *RECQL4* | NM_004260.3 | c.3189_3217del | p.A1063Pfs*22 | frameshift variant | Novel | 0;0 | -;-;0 | Likely Pathogenic | WT | DDR | 65 | OCP |
| P26 | *DICER1* | NM_001195573.1 | c.5483G>A | p.W1828* | stop gain | rs755081246 | 0;0 | .;.;45 | Likely Pathogenic | WT | No | 53 | OCP |
| P27 | *NF2* | NM_000268.3 | c.348_349insA | p.L117Ifs*13 | frameshift variant | Novel | 0;0 | -;-;0 | Likely Pathogenic | 22 q loss | No | 71 | OCP |
| P28 | *NOTCH3* | NM_000435.2 | c.545G>C | p.R182P | missense variant | Novel | 0;0 | D;D;16.55 | Likely Pathogenic | WT | No | 22 | OCP |
| P29 | *PPM1D* | NM_003620.3 | c.1426G>T | p.E476* | stop gain | Novel | 0;0 | T;.;39 | Likely Pathogenic | undetermined | No | 50 | OCP |
| P30 | *PTCH1* | NM_000264.3 | c.4187dup | p.L1397Tfs*7 | frameshift variant | Novel | 0;0 | .;.;45 | Likely Pathogenic | WT | No | 32 | OCP |
| P31 | *SLX4* | NM_032444.2 | c.1047_1048insG | p.T350Dfs*30 | frameshift variant | Novel | 0;0 | -;-;0 | Likely Pathogenic | WT | No | 33 | OCP |
| P32 | *TSHR* | NM_000369.2 | c.2284del | p.T762Rfs*13 | frameshift variant | Novel | 0;0 | -;-;0 | Likely Pathogenic | WT | No | 37 | OCP |

Supplementary Table S3. Clinical characteristics of patients with RCC

|  | Characteristic | SRP | OCP | NCP | p |
| --- | --- | --- | --- | --- | --- |
| Gender | Male | 6 | 12 | 199 | 0.29 |
|  | Female | 6 | 8 | 91 |  |
| Age | ≤46 | 7 | 5 | 59 | 0.012 |
|  | >46 | 5 | 15 | 231 |  |
| Smoking status | Yes | 3 | 5 | 121 | 0.13 |
|  | No | 9 | 14 | 145 |  |
|  | Unknown | 0 | 1 | 24 |  |
| Clinical stage | I-II | 5 | 5 | 97 | 0.53 |
|  | III-IV | 3 | 8 | 122 |  |
|  | Unknown | 4 | 7 | 71 |  |

Supplementary Table S4. Somatic variation of top 10 genes in different groups

| Gene | Mutation status | SRP | OCP | NCP | p |
| --- | --- | --- | --- | --- | --- |
| *VHL* | + | 1 | 6 | 140 | 0.002 |
|  | - | 9 | 12 | 106 |  |
| *PBRM1* | + | 0 | 4 | 66 | 0.16 |
|  | - | 10 | 14 | 180 |  |
| *SETD2* | + | 1 | 3 | 36 | 0.91 |
|  | - | 9 | 15 | 210 |  |
| *GNAQ* | + | 3 | 2 | 29 | 0.23 |
|  | - | 7 | 16 | 217 |  |
| *BAP1* | + | 0 | 0 | 30 | 0.22 |
|  | - | 10 | 18 | 216 |  |
| *INPPL1* | + | 1 | 1 | 20 | 0.85 |
|  | - | 9 | 17 | 226 |  |
| *ARID1A* | + | 0 | 1 | 18 | 1.00 |
|  | - | 10 | 17 | 228 |  |
| *KMT2D* | + | 0 | 4 | 16 | 0.07 |
|  | - | 10 | 14 | 230 |  |
| *KMT2C* | + | 0 | 1 | 14 | 1.00 |
|  | - | 10 | 17 | 232 |  |
| *ZFHX3* | + | 1 | 0 | 15 | 0.48 |
|  | - | 9 | 18 | 231 |  |

Supplementary Tables S 5: Details of somatic variants detected via next-generation sequencing

| SampleID | Gene | Transcript | Variant | Amino acid change | Consequence | MutFreq/Copy number ratio |
| --- | --- | --- | --- | --- | --- | --- |
| P69 | ABL1 | NM_005157 | c.278A>G | p.Y93C | nonsynonymous SNV | 0.01036 |
| P70 | ABL2 | NM_001168236 | c.646C>T | p.R216C | nonsynonymous SNV | 0.01306 |
| P5 | ACVR1B | NM_004302 | c.465_466insTT | p.V155fs | frameshift insertion | 0.4436 |
| P222 | ACVR2A | NM_001278580 | c.995_996del | p.V332fs | frameshift deletion | 0.08425 |
| P71 | ADGRA2 | NM_032777 | c.1175G>A | p.G392E | nonsynonymous SNV | 0.01049 |
| P61 | ADGRG4 | NM_153834 | c.4565C>T | p.A1522V | nonsynonymous SNV | 0.04436 |
| P72 | AGO2 | NM_001164623 | c.980G>T | p.C327F | nonsynonymous SNV | 0.49415 |
| P49 | AGO2 | NM_001164623 | c.2158A>T | p.K720X | stopgain | 0.24886 |
| P49 | AKT1 | NM_005163 | c.49G>A | p.E17K | nonsynonymous SNV | 0.3824 |
| P61 | AKT1 | NM_005163 | c.155T>G | p.L52R | nonsynonymous SNV | 0.27473 |
| P30 | AKT1 | NM_005163 | c.49G>A | p.E17K | nonsynonymous SNV | 0.45681 |
| P73 | AKT2 | NM_001626 | c.910G>A | p.G304R | nonsynonymous SNV | 0.01376 |
| P69 | AKT2 | NM_001626 | c.484A>G | p.T162A | nonsynonymous SNV | 0.09581 |
| P74 | AKT3 | NM_005465 | c.497G>A | p.R166Q | nonsynonymous SNV | 0.01499 |
| P75 | ALK | NM_004304 | c.2729T>C | p.M910T | nonsynonymous SNV | 0.48526 |
| P76 | ALK | NM_004304 | c.191T>A | p.F64Y | nonsynonymous SNV | 0.18499 |
| P77 | ALK | NM_004304 | c.599G>A | p.R200K | nonsynonymous SNV | 0.37037 |
| P78 | ALK | NM_004304 | c.3170T>C | p.I1057T | nonsynonymous SNV | 0.02384 |
| P78 | ALK | NM_004304 | c.386G>T | p.G129V | nonsynonymous SNV | 0.02396 |
| P79 | ALK | NM_004304 | c.1042G>A | p.A348T | nonsynonymous SNV | 0.49735 |
| P80 | ALK | NM_004304 | c.3595A>C | p.M1199L | nonsynonymous SNV | 0.0227 |
| P225 | ALK | NM_004304 | c.4573_4575del | p.1525_1525del | nonframeshift deletion | 0.12258 |
| P39 | ALK | - | - | - | gain | 4.66 |
| P63 | ALK | - | - | - | gain | 4.654 |
| P81 | ALOX12B | NM_001139 | c.1565C>T | p.P522L | nonsynonymous SNV | 0.02807 |
| P82 | AMER1 | NM_152424 | c.2733G>T | p.L911F | nonsynonymous SNV | 0.01092 |
| P83 | AMFR | NM_001144 | c.1275T>A | p.D425E | nonsynonymous SNV | 0.02149 |
| P19 | AMFR | NM_001144 | c.237dupA | p.D80fs | frameshift insertion | 0.02773 |
| P40 | ANK2 | NM_001148 | c.6338A>G | p.E2113G | nonsynonymous SNV | 0.01819 |
| P84 | ANK2 | NM_001148 | c.8831C>G | p.T2944S | nonsynonymous SNV | 0.0973 |
| P183 | ANK2 | NM_001148 | c.10361dupG | p.R3454fs | frameshift insertion | 0.01006 |
| P38 | ANKRD11 | NM_001256182 | c.3902G>A | p.S1301N | nonsynonymous SNV | 0.01818 |
| P81 | ANKRD11 | NM_001256182 | c.5569C>T | p.L1857F | nonsynonymous SNV | 0.03547 |
| P61 | ANKRD11 | NM_001256182 | c.5665A>G | p.K1889E | nonsynonymous SNV | 0.02375 |
| P24 | ANKRD11 | NM_001256182 | c.6421C>T | p.L2141F | nonsynonymous SNV | 0.10828 |
| P85 | ANKRD11 | NM_001256182 | c.3164G>A | p.C1055Y | nonsynonymous SNV | 0.19606 |
| P226 | ANO3 | NM_001313726 | c.1872delT | p.T624fs | frameshift deletion | 0.37681 |
| P86 | APC | NM_000038 | c.5001T>G | p.N1667K | nonsynonymous SNV | 0.03692 |
| P87 | APC | NM_000038 | c.4340A>C | p.Q1447P | nonsynonymous SNV | 0.02617 |
| P88 | APC | NM_000038 | c.5001T>G | p.N1667K | nonsynonymous SNV | 0.05513 |
| P89 | APC | NM_000038 | c.1739A>T | p.K580I | nonsynonymous SNV | 0.03357 |
| P90 | APC | NM_000038 | c.6785G>A | p.S2262N | nonsynonymous SNV | 0.15476 |
| P91 | APC | NM_000038 | c.5001T>G | p.N1667K | nonsynonymous SNV | 0.03741 |
| P92 | APC | NM_000038 | c.5001T>G | p.N1667K | nonsynonymous SNV | 0.04491 |
| P64 | APC | - | - | - | loss | 0.275 |
| P93 | APOB | NM_000384 | c.5209A>G | p.K1737E | nonsynonymous SNV | 0.05815 |
| P81 | APOB | NM_000384 | c.6551A>G | p.Y2184C | nonsynonymous SNV | 0.0143 |
| P27 | APOB | NM_000384 | c.4163G>A | p.R1388H | nonsynonymous SNV | 0.18327 |
| P64 | AR | NM_000044 | c.179A>T | p.Q60L | nonsynonymous SNV | 0.02799 |
| P94 | AR | NM_000044 | c.1348T>G | p.C450G | nonsynonymous SNV | 0.0241 |
| P55 | AR | NM_000044 | c.173A>T | p.Q58L | nonsynonymous SNV | 0.01689 |
| P95 | AR | NM_000044 | c.194A>T | p.Q65L | nonsynonymous SNV | 0.01091 |
| P46 | AR | NM_000044 | c.182A>T | p.Q61L | nonsynonymous SNV | 0.02332 |
| P96 | ARAF | NM_001654 | c.651C>A | p.N217K | nonsynonymous SNV | 0.01773 |
| P78 | ARAF | NM_001654 | c.1734G>C | p.E578D | nonsynonymous SNV | 0.023 |
| P97 | ARAF | NM_001654 | c.1285G>T | p.D429Y | nonsynonymous SNV | 0.03525 |
| P98 | ARAF | NM_001654 | c.1734G>C | p.E578D | nonsynonymous SNV | 0.55838 |
| P75 | ARID1A | NM_006015 | c.1496C>T | p.S499L | nonsynonymous SNV | 0.4883 |
| P76 | ARID1A | NM_006015 | c.3841T>A | p.Y1281N | nonsynonymous SNV | 0.01682 |
| P99 | ARID1A | NM_006015 | c.2806A>G | p.S936G | nonsynonymous SNV | 0.04385 |
| P100 | ARID1A | NM_006015 | c.3227T>G | p.L1076R | nonsynonymous SNV | 0.18303 |
| P101 | ARID1A | NM_006015 | c.1975C>T | p.P659S | nonsynonymous SNV | 0.02016 |
| P102 | ARID1A | NM_006015 | c.3698A>G | p.Y1233C | nonsynonymous SNV | 0.01393 |
| P19 | ARID1A | NM_006015 | c.6182T>C | p.L2061S | nonsynonymous SNV | 0.04543 |
| P54 | ARID1A | NM_006015 | c.1106_1107del | p.G369fs | frameshift deletion | 0.26739 |
| P203 | ARID1A | NM_006015 | c.4583delG | p.R1528fs | frameshift deletion | 0.20238 |
| P166 | ARID1A | NM_006015 | c.3598C>T | p.Q1200X | stopgain | 0.06017 |
| P139 | ARID1A | NM_006015 | c.1578delA | p.P526fs | frameshift deletion | 0.06903 |
| P199 | ARID1A | NM_006015 | c.4024C>T | p.Q1342X | stopgain | 0.65432 |
| P58 | ARID1A | NM_006015 | c.1444C>T | p.Q482X | stopgain | 0.61098 |
| P189 | ARID1A | NM_006015 | c.666dupC | p.Y222fs | frameshift insertion | 0.01054 |
| P91 | ARID1A | NM_006015 | c.5194A>T | p.K1732X | stopgain | 0.01462 |
| P213 | ARID1A | NM_006015 | c.5689delC | p.P1897fs | frameshift deletion | 0.05936 |
| P213 | ARID1A | NM_006015 | c.5882delG | p.S1961fs | frameshift deletion | 0.25553 |
| P213 | ARID1A | NM_006015 | c.6528dupG | p.Q2176fs | frameshift insertion | 0.27138 |
| P44 | ARID1A | NM_006015 | c.6751delT | p.F2251fs | frameshift deletion | 0.01497 |
| P161 | ARID1B | NM_020732 | c.4571C>A | p.S1524X | stopgain | 0.21512 |
| P139 | ARID1B | NM_020732 | c.5953delC | p.H1985fs | frameshift deletion | 0.07241 |
| P59 | ARID1B | NM_020732 | c.4552C>T | p.Q1518X | stopgain | 0.1448 |
| P138 | ARID1B | NM_020732 | c.2318C>A | p.S773X | stopgain | 0.01903 |
| P103 | ARID2 | NM_152641 | c.1885G>T | p.D629Y | nonsynonymous SNV | 0.01873 |
| P58 | ARID2 | NM_152641 | c.1616delC | p.S539fs | frameshift deletion | 0.42129 |
| P218 | ARID2 | NM_152641 | c.3753delG | p.K1251fs | frameshift deletion | 0.17548 |
| P188 | ARID2 | NM_152641 | c.1941delA | p.G647fs | frameshift deletion | 0.10659 |
| P198 | ARID2 | NM_152641 | c.93-1G>A | - | - | 0.12642 |
| P104 | ARID5B | NM_032199 | c.508G>A | p.D170N | nonsynonymous SNV | 0.52684 |
| P214 | ARID5B | NM_032199 | c.3076delA | p.K1026fs | frameshift deletion | 0.02227 |
| P148 | ARID5B | NM_032199 | c.3076delA | p.K1026fs | frameshift deletion | 0.01179 |
| P184 | ARID5B | NM_032199 | c.3076delA | p.K1026fs | frameshift deletion | 0.01436 |
| P105 | ASXL1 | NM_015338 | c.1544T>G | p.V515G | nonsynonymous SNV | 0.16359 |
| P23 | ASXL1 | NM_015338 | c.3956C>A | p.P1319H | nonsynonymous SNV | 0.01531 |
| P191 | ASXL1 | NM_015338 | c.773_777del | p.I258fs | frameshift deletion | 0.26482 |
| P40 | ASXL2 | NM_018263 | c.3353C>A | p.P1118H | nonsynonymous SNV | 0.25673 |
| P89 | ASXL2 | NM_018263 | c.258T>A | p.D86E | nonsynonymous SNV | 0.02128 |
| P79 | ASXL2 | NM_018263 | c.3908A>C | p.H1303P | nonsynonymous SNV | 0.01253 |
| P27 | ASXL2 | NM_018263 | c.258T>A | p.D86E | nonsynonymous SNV | 0.82836 |
| P106 | ASXL2 | NM_018263 | c.1969C>T | p.L657F | nonsynonymous SNV | 0.19546 |
| P44 | ASXL2 | NM_018263 | c.53A>T | p.K18M | nonsynonymous SNV | 0.36415 |
| P38 | ASXL3 | NM_030632 | c.3984C>A | p.S1328R | nonsynonymous SNV | 0.02637 |
| P38 | ASXL3 | NM_030632 | c.6137C>A | p.P2046H | nonsynonymous SNV | 0.02621 |
| P107 | ASXL3 | NM_030632 | c.6166C>A | p.Q2056K | nonsynonymous SNV | 0.01164 |
| P48 | ATM | NM_000051 | c.8509G>T | p.E2837X | stopgain | 0.2091 |
| P50 | ATM | NM_000051 | c.7436dupA | p.E2479fs | frameshift insertion | 0.11723 |
| P145 | ATM | NM_000051 | c.918delA | p.T306fs | frameshift deletion | 0.0123 |
| P157 | ATM | NM_000051 | c.7456C>T | p.R2486X | stopgain | 0.02041 |
| P83 | ATM | NM_000051 | c.6976-2A>G | - | - | 0.22754 |
| P108 | ATR | NM_001184 | c.2584A>T | p.N862Y | nonsynonymous SNV | 0.24026 |
| P18 | ATR | NM_001184 | c.2938A>T | p.N980Y | nonsynonymous SNV | 0.0839 |
| P213 | ATR | NM_001184 | c.3402delT | p.F1134fs | frameshift deletion | 0.01178 |
| P103 | ATR | NM_001184 | c.712G>T | p.E238X | stopgain | 0.01716 |
| P109 | ATRX | NM_000489 | c.6216A>T | p.K2072N | nonsynonymous SNV | 0.08772 |
| P110 | ATRX | NM_000489 | c.1851T>A | p.S617R | nonsynonymous SNV | 0.10286 |
| P24 | ATRX | NM_000489 | c.586A>T | p.I196F | nonsynonymous SNV | 0.2347 |
| P10 | ATRX | NM_000489 | c.6773T>A | p.L2258X | stopgain | 0.3327 |
| P78 | AURKA | NM_003600 | c.70C>T | p.R24C | nonsynonymous SNV | 0.01692 |
| P15 | AURKB | NM_001256834 | c.203T>C | p.F68S | nonsynonymous SNV | 0.01636 |
| P235 | AXIN1 | NM_003502 | c.1523delG | p.G508fs | frameshift deletion | 0.01071 |
| P111 | AXIN2 | NM_004655 | c.475G>T | p.D159Y | nonsynonymous SNV | 0.09615 |
| P69 | AXIN2 | NM_004655 | c.188G>T | p.G63V | nonsynonymous SNV | 0.12654 |
| P83 | AXIN2 | NM_004655 | c.1964A>T | p.E655V | nonsynonymous SNV | 0.01107 |
| P38 | AXIN2 | NM_004655 | c.169C>T | p.R57W | nonsynonymous SNV | 0.01832 |
| P112 | AXIN2 | NM_004655 | c.287G>A | p.R96Q | nonsynonymous SNV | 0.13699 |
| P167 | AXIN2 | NM_004655 | c.1994dupG | p.G665fs | frameshift insertion | 0.02044 |
| P113 | AXL | NM_001699 | c.890G>T | p.G297V | nonsynonymous SNV | 0.01821 |
| P114 | BACH1 | NM_001186 | c.1580C>T | p.P527L | nonsynonymous SNV | 0.12051 |
| P115 | BAP1 | NM_004656 | c.32A>G | p.D11G | nonsynonymous SNV | 0.125 |
| P38 | BAP1 | NM_004656 | c.1690C>G | p.L564V | nonsynonymous SNV | 0.02519 |
| P116 | BAP1 | NM_004656 | c.47C>T | p.T16I | nonsynonymous SNV | 0.33145 |
| P117 | BAP1 | NM_004656 | c.97T>G | p.Y33D | nonsynonymous SNV | 0.10408 |
| P100 | BAP1 | NM_004656 | c.515G>T | p.S172I | nonsynonymous SNV | 0.21258 |
| P118 | BAP1 | NM_004656 | c.674A>T | p.D225V | nonsynonymous SNV | 0.18652 |
| P119 | BAP1 | NM_004656 | c.517T>G | p.Y173D | nonsynonymous SNV | 0.13281 |
| P120 | BAP1 | NM_004656 | c.518A>G | p.Y173C | nonsynonymous SNV | 0.46176 |
| P121 | BAP1 | NM_004656 | c.538C>T | p.L180F | nonsynonymous SNV | 0.2293 |
| P243 | BAP1 | NM_004656 | c.325G>T | p.G109X | stopgain | 0.32886 |
| P142 | BAP1 | NM_004656 | c.2050C>T | p.Q684X | stopgain | 0.15625 |
| P205 | BAP1 | NM_004656 | c.1764_1771del | p.P588fs | frameshift deletion | 0.41611 |
| P158 | BAP1 | NM_004656 | c.1063C>T | p.Q355X | stopgain | 0.25663 |
| P234 | BAP1 | NM_004656 | c.1437delC | p.P479fs | frameshift deletion | 0.24359 |
| P231 | BAP1 | NM_004656 | c.2101_2107del | p.R701fs | frameshift deletion | 0.03629 |
| P193 | BAP1 | NM_004656 | c.799_800del | p.Q267fs | frameshift deletion | 0.06803 |
| P161 | BAP1 | NM_004656 | c.219_223del | p.D73fs | frameshift deletion | 0.30806 |
| P48 | BAP1 | NM_004656 | c.772delG | p.A258fs | frameshift deletion | 0.22628 |
| P81 | BAP1 | NM_004656 | c.973_979del | p.S325fs | frameshift deletion | 0.4051 |
| P71 | BAP1 | NM_004656 | c.829C>T | p.Q277X | stopgain | 0.09836 |
| P129 | BAP1 | NM_004656 | c.79delG | p.V27fs | frameshift deletion | 0.57631 |
| P122 | BAP1 | NM_004656 | c.2104C>T | p.Q702X | stopgain | 0.10957 |
| P127 | BAP1 | NM_004656 | c.1073delC | p.P358fs | frameshift deletion | 0.20497 |
| P214 | BAP1 | NM_004656 | c.2098_2110del | p.R700fs | frameshift deletion | 0.18182 |
| P218 | BAP1 | NM_004656 | c.233delA | p.N78fs | frameshift deletion | 0.29602 |
| P162 | BAP1 | NM_004656 | c.324_364del | p.L108fs | frameshift deletion | 0.11502 |
| P171 | BAP1 | NM_004656 | c.1651delC | p.R551fs | frameshift deletion | 0.35696 |
| P201 | BAP1 | NM_004656 | c.2056+1G>C | - | - | 0.05442 |
| P131 | BAP1 | NM_004656 | c.68-1G>C | - | - | 0.10901 |
| P219 | BAP1 | NM_004656 | c.67+1G>C | - | - | 0.02719 |
| P61 | BARD1 | NM_000465 | c.615_616del | p.K205fs | frameshift deletion | 0.05617 |
| P72 | BARD1 | NM_000465 | c.615_616del | p.K205fs | frameshift deletion | 0.01461 |
| P122 | BAX | NM_004324 | c.29G>A | p.G10D | nonsynonymous SNV | 0.0807 |
| P123 | BBC3 | NM_001127240 | c.290A>C | p.H97P | nonsynonymous SNV | 0.01401 |
| P124 | BBC3 | NM_001127240 | c.298A>C | p.T100P | nonsynonymous SNV | 0.01685 |
| P75 | BBC3 | NM_001127240 | c.290A>C | p.H97P | nonsynonymous SNV | 0.02568 |
| P87 | BBC3 | NM_001127240 | c.290A>C | p.H97P | nonsynonymous SNV | 0.01192 |
| P125 | BBC3 | NM_001127240 | c.290A>C | p.H97P | nonsynonymous SNV | 0.01213 |
| P14 | BBC3 | NM_001127240 | c.290A>C | p.H97P | nonsynonymous SNV | 0.02817 |
| P126 | BBC3 | NM_001127240 | c.290A>C | p.H97P | nonsynonymous SNV | 0.01391 |
| P79 | BBC3 | NM_001127240 | c.290A>C | p.H97P | nonsynonymous SNV | 0.0134 |
| P127 | BBC3 | NM_001127240 | c.290A>C | p.H97P | nonsynonymous SNV | 0.01176 |
| P128 | BBC3 | NM_001127240 | c.290A>C | p.H97P | nonsynonymous SNV | 0.02672 |
| P46 | BCL2L1 | NM_001317921 | c.25G>A | p.V9M | nonsynonymous SNV | 0.0796 |
| P129 | BCOR | NM_001123383 | c.2429G>C | p.R810P | nonsynonymous SNV | 0.01431 |
| P130 | BCOR | NM_001123383 | c.2365C>G | p.P789A | nonsynonymous SNV | 0.25532 |
| P118 | BCOR | NM_001123383 | c.3482A>G | p.K1161R | nonsynonymous SNV | 0.0185 |
| P93 | BCORL1 | NM_021946 | c.2615T>G | p.V872G | nonsynonymous SNV | 0.01096 |
| P131 | BCORL1 | NM_021946 | c.3247C>T | p.R1083W | nonsynonymous SNV | 0.01126 |
| P90 | BCORL1 | NM_021946 | c.2057C>A | p.P686H | nonsynonymous SNV | 0.22502 |
| P244 | BCORL1 | NM_021946 | c.3750delC | p.F1250fs | frameshift deletion | 0.06076 |
| P218 | BCORL1 | NM_001184772 | c.4324C>T | p.Q1442X | stopgain | 0.1668 |
| P162 | BCORL1 | NM_021946 | c.3888G>A | p.W1296X | stopgain | 0.03043 |
| P132 | BCR | NM_004327 | c.3611C>G | p.A1204G | nonsynonymous SNV | 0.01923 |
| P133 | BCR | NM_004327 | c.497G>T | p.G166V | nonsynonymous SNV | 0.44659 |
| P167 | BCR | NM_004327 | c.1147dupC | p.S382fs | frameshift insertion | 0.01931 |
| P61 | BLM | NM_000057 | c.2371C>T | p.R791C | nonsynonymous SNV | 0.01613 |
| P24 | BLM | NM_000057 | c.3062A>G | p.N1021S | nonsynonymous SNV | 0.01327 |
| P63 | BLM | - | - | - | loss | 0.553 |
| P71 | BMPR1A | NM_004329 | c.64C>T | p.Q22X | stopgain | 0.03559 |
| P124 | BORCS8-MEF2B;MEF2B | NM_005919 | c.54+1G>A | - | - | 0.50871 |
| P61 | BRAF | NM_004333 | c.1781A>G | p.D594G | nonsynonymous SNV | 0.21741 |
| P58 | BRAF | NM_004333 | c.1742A>C | p.N581T | nonsynonymous SNV | 0.01022 |
| P30 | BRAF | NM_004333 | c.1799T>A | p.V600E | nonsynonymous SNV | 0.45077 |
| P26 | BRAF | NM_004333 | c.245A>C | p.Y82S | nonsynonymous SNV | 0.02178 |
| P117 | BRAF | NM_004333 | c.1208delC | p.P403fs | frameshift deletion | 0.01357 |
| P167 | BRAF | NM_004333 | c.1023_1024insTTCCA | p.I342fs | frameshift insertion | 0.04075 |
| P9 | BRAF | NM_004333 | c.1208delC | p.P403fs | frameshift deletion | 0.01146 |
| P134 | BRCA1 | NM_007294 | c.811G>A | p.V271M | nonsynonymous SNV | 0.18825 |
| P88 | BRCA1 | NM_007294 | c.3681A>T | p.Q1227H | nonsynonymous SNV | 0.22924 |
| P135 | BRCA1 | NM_007294 | c.637A>T | p.R213W | nonsynonymous SNV | 0.28767 |
| P163 | BRCA1 | NM_007294 | c.3756_3759del | p.L1252fs | frameshift deletion | 0.25797 |
| P151 | BRCA1 | NM_007294 | c.4096+2T>C | - | - | 0.1116 |
| P6 | BRCA2 | NM_000059 | c.1744A>C | p.T582P | nonsynonymous SNV | 0.41083 |
| P108 | BRCA2 | NM_000059 | c.1092T>A | p.D364E | nonsynonymous SNV | 0.22782 |
| P81 | BRCA2 | NM_000059 | c.1973C>T | p.S658F | nonsynonymous SNV | 0.01193 |
| P89 | BRCA2 | NM_000059 | c.7117A>T | p.S2373C | nonsynonymous SNV | 0.02323 |
| P106 | BRCA2 | NM_000059 | c.5018C>A | p.T1673K | nonsynonymous SNV | 0.01782 |
| P91 | BRCA2 | NM_000059 | c.4995A>T | p.E1665D | nonsynonymous SNV | 0.01818 |
| P103 | BRCA2 | NM_000059 | c.3583C>A | p.L1195M | nonsynonymous SNV | 0.01355 |
| P15 | BRCA2 | NM_000059 | c.7559G>A | p.R2520Q | nonsynonymous SNV | 0.02304 |
| P9 | BRCA2 | - | - | - | loss | 0.74 |
| P63 | BRCA2 | - | - | - | loss | 0.316 |
| P38 | BRD4 | NM_058243 | c.2498C>A | p.P833H | nonsynonymous SNV | 0.01317 |
| P136 | BRD4 | NM_058243 | c.80C>A | p.S27Y | nonsynonymous SNV | 0.10526 |
| P5 | BRD4 | NM_058243 | c.2873T>C | p.L958P | nonsynonymous SNV | 0.03175 |
| P72 | BRD4 | NM_058243 | c.1645G>A | p.E549K | nonsynonymous SNV | 0.01554 |
| P38 | BRD4 | NM_058243 | c.2728delC | p.Q910fs | frameshift deletion | 0.01332 |
| P167 | BRD4 | NM_058243 | c.2872delC | p.L958fs | frameshift deletion | 0.03784 |
| P60 | BRD4 | NM_058243 | c.4079delA | p.N1360fs | frameshift deletion | 0.32864 |
| P90 | BRD4 | NM_058243 | c.2728delC | p.Q910fs | frameshift deletion | 0.01133 |
| P37 | BRD4 | - | - | - | gain | 4.36 |
| P38 | BRD4 | - | - | - | gain | 4.2 |
| P67 | BRD4 | - | - | - | gain | 9.459 |
| P96 | BRINP3 | NM_199051 | c.2009G>T | p.G670V | nonsynonymous SNV | 0.01741 |
| P137 | BRINP3 | NM_199051 | c.1268C>T | p.T423M | nonsynonymous SNV | 0.40526 |
| P93 | BRIP1 | NM_032043 | c.430G>A | p.A144T | nonsynonymous SNV | 0.09016 |
| P138 | BRIP1 | NM_032043 | c.3433G>A | p.E1145K | nonsynonymous SNV | 0.03063 |
| P27 | BRIP1 | NM_032043 | c.2440C>T | p.R814C | nonsynonymous SNV | 0.31379 |
| P139 | BTG1 | NM_001731 | c.168G>C | p.W56C | nonsynonymous SNV | 0.03497 |
| P140 | BTK | NM_000061 | c.556A>G | p.K186E | nonsynonymous SNV | 0.01175 |
| P72 | BTK | NM_000061 | c.1764G>A | p.W588X | stopgain | 0.17179 |
| P141 | C6orf118 | NM_144980 | c.629C>T | p.T210I | nonsynonymous SNV | 0.03341 |
| P120 | CARD11 | NM_032415 | c.1232A>T | p.E411V | nonsynonymous SNV | 0.30778 |
| P13 | CARD11 | NM_032415 | c.1663delC | p.R555fs | frameshift deletion | 0.01054 |
| P230 | CARD11 | NM_032415 | c.2846dupA | p.D949fs | frameshift insertion | 0.2483 |
| P236 | CARD11 | NM_032415 | c.839delA | p.N280fs | frameshift deletion | 0.01083 |
| P122 | CARD11 | NM_032415 | c.286G>T | p.E96X | stopgain | 0.1311 |
| P102 | CARD11 | NM_032415 | c.1663delC | p.R555fs | frameshift deletion | 0.01174 |
| P49 | CARM1 | NM_199141 | c.313A>T | p.N105Y | nonsynonymous SNV | 0.22063 |
| P106 | CARM1 | NM_199141 | c.20C>T | p.A7V | nonsynonymous SNV | 0.01013 |
| P87 | CBL | NM_005188 | c.2491A>T | p.I831F | nonsynonymous SNV | 0.0213 |
| P77 | CBL | NM_005188 | c.749C>T | p.P250L | nonsynonymous SNV | 0.22511 |
| P118 | CBL | NM_005188 | c.1211G>A | p.C404Y | nonsynonymous SNV | 0.01507 |
| P170 | CBL | NM_005188 | c.1104T>G | p.Y368X | stopgain | 0.12833 |
| P5 | CBL | NM_005188 | c.2322T>G | p.Y774X | stopgain | 0.044 |
| P36 | CBL | NM_005188 | c.1561A>T | p.K521X | stopgain | 0.22836 |
| P142 | CCDC6 | NM_005436 | c.13G>A | p.A5T | nonsynonymous SNV | 0.11907 |
| P61 | CCNE1 | NM_001322262 | c.758C>T | p.P253L | nonsynonymous SNV | 0.03598 |
| P143 | CD22 | NM_001185101 | c.443C>A | p.P148H | nonsynonymous SNV | 0.50561 |
| P109 | CD22 | NM_001278417 | c.421A>T | p.N141Y | nonsynonymous SNV | 0.14317 |
| P144 | CD22 | NM_001185101 | c.275T>A | p.V92E | nonsynonymous SNV | 0.43025 |
| P10 | CD274 | NM_001267706 | c.83T>C | p.L28S | nonsynonymous SNV | 0.50034 |
| P145 | CD274 | NM_001267706 | c.67A>G | p.I23V | nonsynonymous SNV | 0.02808 |
| P245 | CD3EAP | NM_012099 | c.1510delC | p.Q504fs | frameshift deletion | 0.01832 |
| P224 | CD74 | NM_001025159 | c.878delC | p.P293fs | frameshift deletion | 0.01662 |
| P146 | CD79A | NM_021601 | c.209T>A | p.L70H | nonsynonymous SNV | 0.30416 |
| P219 | CDC42 | NM_001039802 | c.293delT | p.V98fs | frameshift deletion | 0.16149 |
| P219 | CDC42 | NM_001039802 | c.296_297del | p.P99fs | frameshift deletion | 0.15751 |
| P20 | CDH1 | NM_004360 | c.1852G>A | p.D618N | nonsynonymous SNV | 0.19742 |
| P138 | CDH1 | NM_004360 | c.74C>T | p.P25L | nonsynonymous SNV | 0.02346 |
| P93 | CDK12 | NM_016507 | c.4402T>C | p.Y1468H | nonsynonymous SNV | 0.10549 |
| P27 | CDK12 | NM_016507 | c.2960C>T | p.S987F | nonsynonymous SNV | 0.39236 |
| P44 | CDK12 | NM_016507 | c.2427T>G | p.F809L | nonsynonymous SNV | 0.31732 |
| P175 | CDK12 | NM_016507 | c.2965delA | p.I989fs | frameshift deletion | 0.22422 |
| P38 | CDK4 | - | - | - | gain | 4.52 |
| P44 | CDK4 | - | - | - | gain | 5.61 |
| P45 | CDK4 | - | - | - | gain | 5.62 |
| P115 | CDK8 | NM_001260 | c.188C>T | p.A63V | nonsynonymous SNV | 0.05568 |
| P144 | CDK8 | NM_001346501 | c.400A>G | p.K134E | nonsynonymous SNV | 0.36382 |
| P147 | CDKN1A | NM_078467 | c.113T>A | p.M38K | nonsynonymous SNV | 0.03255 |
| P117 | CDKN1B | NM_004064 | c.299A>T | p.K100M | nonsynonymous SNV | 0.07938 |
| P213 | CDKN1B | NM_004064 | c.94_95del | p.L32fs | frameshift deletion | 0.23319 |
| P219 | CDKN1C | NM_001122630 | c.68dupT | p.F23fs | frameshift insertion | 0.21039 |
| P106 | CDKN2A | NM_000077 | c.211A>T | p.N71Y | nonsynonymous SNV | 0.21623 |
| P148 | CDKN2A | NM_058195 | c.146T>C | p.L49P | nonsynonymous SNV | 0.01098 |
| P65 | CDKN2A | - | - | - | loss | 0.89 |
| P65 | CDKN2B | - | - | - | loss | 0.77 |
| P253 | CDKN2C | NM_078626 | c.431delC | p.A144fs | frameshift deletion | 0.02895 |
| P254 | CDKN2C | NM_078626 | c.348delC | p.L116fs | frameshift deletion | 0.25538 |
| P144 | CEBPA | NM_004364 | c.818A>T | p.K273M | nonsynonymous SNV | 0.42063 |
| P50 | CEBPA | NM_004364 | c.936_937insCAG | p.K313delinsQK | nonframeshift insertion | 0.05005 |
| P69 | CFTR | NM_000492 | c.3010G>T | p.A1004S | nonsynonymous SNV | 0.0135 |
| P93 | CFTR | NM_000492 | c.374T>C | p.I125T | nonsynonymous SNV | 0.10126 |
| P38 | CFTR | NM_000492 | c.4195C>T | p.L1399F | nonsynonymous SNV | 0.04377 |
| P126 | CHD1 | NM_001270 | c.3779A>T | p.N1260I | nonsynonymous SNV | 0.18708 |
| P57 | CHD1 | NM_001270 | c.3506G>A | p.G1169E | nonsynonymous SNV | 0.30718 |
| P64 | CHD1 | - | - | - | loss | 0.091 |
| P91 | CHD2 | NM_001271 | c.2749A>T | p.T917S | nonsynonymous SNV | 0.07096 |
| P137 | CHD2 | NM_001271 | c.224C>T | p.S75F | nonsynonymous SNV | 0.44976 |
| P81 | CHD4 | NM_001273 | c.77A>G | p.N26S | nonsynonymous SNV | 0.30994 |
| P91 | CHD4 | NM_001273 | c.656A>T | p.K219I | nonsynonymous SNV | 0.2116 |
| P44 | CHD4 | NM_001273 | c.5389G>C | p.E1797Q | nonsynonymous SNV | 0.05005 |
| P149 | CHD4 | NM_001273 | c.212A>C | p.Q71P | nonsynonymous SNV | 0.20958 |
| P206 | CHD4 | NM_001273 | c.218dupA | p.K73fs | frameshift insertion | 0.02907 |
| P150 | CHEK2 | NM_007194 | c.1449C>G | p.H483Q | nonsynonymous SNV | 0.02914 |
| P39 | CHEK2 | NM_007194 | c.314A>G | p.N105S | nonsynonymous SNV | 0.06078 |
| P3 | CHEK2 | NM_007194 | c.538C>T | p.R180C | nonsynonymous SNV | 0.0431 |
| P133 | CHEK2 | NM_007194 | c.1118A>G | p.K373R | nonsynonymous SNV | 0.45874 |
| P141 | CHEK2 | NM_007194 | c.334A>T | p.N112Y | nonsynonymous SNV | 0.19262 |
| P133 | CHEK2 | NM_007194 | c.1116_1117TG | - | nonframeshift substitution | 0.01697 |
| P63 | CHEK2 | - | - | - | loss | 0.248 |
| P5 | CHEK2 | - | - | - | loss | 0.005 |
| P152 | CHUK | NM_001278 | c.1936delA | p.R646fs | frameshift deletion | 0.0134 |
| P106 | CIC | NM_001304815 | c.3061C>T | p.H1021Y | nonsynonymous SNV | 0.02506 |
| P151 | CIC | NM_001304815 | c.5751G>C | p.Q1917H | nonsynonymous SNV | 0.05301 |
| P152 | CIC | NM_001304815 | c.1244C>T | p.P415L | nonsynonymous SNV | 0.10169 |
| P255 | CIC | NM_001304815 | c.59delC | p.S20fs | frameshift deletion | 0.01256 |
| P48 | CIC | NM_001304815 | c.7511delC | p.S2504fs | frameshift deletion | 0.01195 |
| P244 | CIC | NM_001304815 | c.59delC | p.S20fs | frameshift deletion | 0.01032 |
| P71 | CIC | NM_001304815 | c.7511delC | p.S2504fs | frameshift deletion | 0.01016 |
| P88 | CIC | NM_001304815 | c.59delC | p.S20fs | frameshift deletion | 0.01198 |
| P250 | CIC | NM_001304815 | c.59delC | p.S20fs | frameshift deletion | 0.0109 |
| P128 | CIC | NM_001304815 | c.59delC | p.S20fs | frameshift deletion | 0.0119 |
| P40 | CIC | - | - | - | loss | 0.95 |
| P76 | CLSPN | NM_001330490 | c.2132G>T | p.G711V | nonsynonymous SNV | 0.05923 |
| P81 | COL22A1 | NM_152888 | c.4537C>T | p.R1513W | nonsynonymous SNV | 0.01911 |
| P144 | CREBBP | NM_004380 | c.4764G>C | p.K1588N | nonsynonymous SNV | 0.40791 |
| P82 | CREBBP | NM_004380 | c.2394_2397del | p.Q798fs | frameshift deletion | 0.01022 |
| P58 | CREBBP | NM_004380 | c.2908_2909del | p.N970fs | frameshift deletion | 0.30049 |
| P57 | CRKL | - | - | - | gain | 6.58 |
| P89 | CSF1R | NM_005211 | c.835G>A | p.V279M | nonsynonymous SNV | 0.05233 |
| P153 | CSF1R | NM_005211 | c.608C>A | p.P203Q | nonsynonymous SNV | 0.24611 |
| P120 | CSF3R | NM_000760 | c.2329T>A | p.L777M | nonsynonymous SNV | 0.31852 |
| P109 | CSMD3 | NM_198123 | c.2182C>A | p.P728T | nonsynonymous SNV | 0.03991 |
| P154 | CSMD3 | NM_198123 | c.8706T>G | p.N2902K | nonsynonymous SNV | 0.01045 |
| P44 | CSMD3 | NM_198123 | c.9004C>T | p.P3002S | nonsynonymous SNV | 0.05509 |
| P155 | CSMD3 | NM_198123 | c.4111C>A | p.H1371N | nonsynonymous SNV | 0.20498 |
| P15 | CSMD3 | NM_198123 | c.2518C>T | p.P840S | nonsynonymous SNV | 0.3122 |
| P111 | CSMD3 | NM_198123 | c.10223-2A>T | - | - | 0.23459 |
| P156 | CTCF | NM_001191022 | c.94A>G | p.S32G | nonsynonymous SNV | 0.05853 |
| P134 | CTNNA1 | NM_001290310 | c.1285G>A | p.V429I | nonsynonymous SNV | 0.0197 |
| P157 | CTNNA2 | NM_001164883 | c.431C>T | p.A144V | nonsynonymous SNV | 0.286 |
| P99 | CUL3 | NM_003590 | c.49C>T | p.R17W | nonsynonymous SNV | 0.08167 |
| P93 | CUL3 | NM_003590 | c.419_441del | p.Y140fs | frameshift deletion | 0.02746 |
| P158 | CXCR4 | NM_001348059 | c.577G>A | p.V193I | nonsynonymous SNV | 0.01391 |
| P72 | CXCR4 | NM_001348059 | c.1006dupG | p.A336fs | frameshift insertion | 0.02 |
| P63 | CYLD | - | - | - | loss | 0.366 |
| P5 | CYLD | - | - | - | loss | 0.023 |
| P159 | DDR1 | NM_001297652 | c.535C>T | p.R179W | nonsynonymous SNV | 0.3589 |
| P48 | DDR1 | NM_001297652 | c.2020C>T | p.Q674X | stopgain | 0.01018 |
| P160 | DDR2 | NM_006182 | c.1958T>A | p.I653N | nonsynonymous SNV | 0.02188 |
| P40 | DICER1 | NM_030621 | c.1611T>A | p.F537L | nonsynonymous SNV | 0.25898 |
| P161 | DICER1 | NM_030621 | c.3715A>C | p.S1239R | nonsynonymous SNV | 0.06903 |
| P151 | DICER1 | NM_030621 | c.5501A>T | p.Y1834F | nonsynonymous SNV | 0.09721 |
| P162 | DICER1 | NM_030621 | c.3703T>C | p.C1235R | nonsynonymous SNV | 0.15769 |
| P103 | DICER1 | NM_030621 | c.1780G>T | p.V594F | nonsynonymous SNV | 0.02525 |
| P156 | DIS3 | NM_001128226 | c.263C>T | p.S88F | nonsynonymous SNV | 0.11602 |
| P163 | DIS3 | NM_014953 | c.2611G>T | p.G871W | nonsynonymous SNV | 0.25056 |
| P256 | DIS3 | NM_014953 | c.491delC | p.S164X | stopgain | 0.08696 |
| P143 | DIS3 | NM_014953 | c.2401_2405del | p.Y801fs | frameshift deletion | 0.05852 |
| P43 | DMD | NM_000109 | c.4150C>A | p.Q1384K | nonsynonymous SNV | 0.03823 |
| P32 | DNMT1 | NM_001379 | c.4557G>C | p.W1519C | nonsynonymous SNV | 0.25549 |
| P135 | DNMT1 | NM_001379 | c.2852A>T | p.K951M | nonsynonymous SNV | 0.28629 |
| P68 | DNMT3A | NM_022552 | c.2228C>T | p.P743L | nonsynonymous SNV | 0.01187 |
| P164 | DNMT3A | NM_022552 | c.1097G>C | p.R366P | nonsynonymous SNV | 0.01059 |
| P55 | DNMT3A | NM_022552 | c.946delA | p.T316fs | frameshift deletion | 0.01179 |
| P194 | DNMT3A | NM_022552 | c.2375_2376del | p.R792fs | frameshift deletion | 0.31503 |
| P89 | DNMT3B | NM_001207055 | c.466G>T | p.G156W | nonsynonymous SNV | 0.03936 |
| P165 | DNMT3B | NM_001207055 | c.2046G>C | p.R682S | nonsynonymous SNV | 0.29443 |
| P123 | DOT1L | NM_032482 | c.362A>G | p.D121G | nonsynonymous SNV | 0.01331 |
| P110 | DOT1L | NM_032482 | c.4171G>A | p.E1391K | nonsynonymous SNV | 0.059 |
| P166 | DOT1L | NM_032482 | c.3905G>A | p.G1302E | nonsynonymous SNV | 0.05691 |
| P167 | DOT1L | NM_032482 | c.3508C>T | p.R1170W | nonsynonymous SNV | 0.02989 |
| P99 | DOT1L | NM_032482 | c.1871A>T | p.Q624L | nonsynonymous SNV | 0.15809 |
| P47 | DOT1L | NM_032482 | c.22A>G | p.R8G | nonsynonymous SNV | 0.01283 |
| P168 | DPYD | NM_000110 | c.1715T>A | p.L572H | nonsynonymous SNV | 0.23994 |
| P35 | DROSHA | NM_013235 | c.1528C>A | p.R510S | nonsynonymous SNV | 0.01059 |
| P201 | DROSHA | NM_013235 | c.2584_2593del | p.M862fs | frameshift deletion | 0.13117 |
| P223 | DUSP27 | NM_001080426 | c.3126delC | p.F1042fs | frameshift deletion | 0.03829 |
| P169 | EED | NM_001308007 | c.1179T>A | p.F393L | nonsynonymous SNV | 0.1079 |
| P85 | EED | NM_001308007 | c.696_705del | p.V232fs | frameshift deletion | 0.10962 |
| P5 | EGFL7 | NM_201446 | c.418G>A | p.E140K | nonsynonymous SNV | 0.17405 |
| P133 | EGFL7 | NM_201446 | c.330delA | p.P110fs | frameshift deletion | 0.0101 |
| P39 | EGFL7 | NM_016215 | c.636+1G>A | - | - | 0.02162 |
| P151 | EGFL7 | NM_016215 | c.636+1G>A | - | - | 0.02806 |
| P170 | EGFR | NM_005228 | c.1015G>A | p.G339R | nonsynonymous SNV | 0.14286 |
| P59 | EGFR | NM_005228 | c.2318A>G | p.H773R | nonsynonymous SNV | 0.83532 |
| P171 | EGFR | NM_005228 | c.2885G>A | p.R962H | nonsynonymous SNV | 0.03275 |
| P35 | EGFR | - | - | - | gain | 5 |
| P59 | EGFR | - | - | - | gain | 12.82 |
| P63 | EGFR | - | - | - | gain | 4.64 |
| P232 | EIF1AX | NM_001412 | c.430-2A>T | - | - | 0.01178 |
| P203 | ELF3 | NM_004433 | c.653delA | p.D218fs | frameshift deletion | 0.1723 |
| P61 | ELF3 | NM_004433 | c.536_585del | p.Y179fs | frameshift deletion | 0.19122 |
| P44 | ELF3 | NM_004433 | c.796dupA | p.K265fs | frameshift insertion | 0.16408 |
| P110 | ELOC | NM_001204864 | c.188A>G | p.Y63C | nonsynonymous SNV | 0.05639 |
| P46 | ELOC | NM_001204864 | c.188A>G | p.Y63C | nonsynonymous SNV | 0.08451 |
| P147 | ELOC | NM_001204864 | c.26A>T | p.D9V | nonsynonymous SNV | 0.36128 |
| P92 | EML4 | NM_019063 | c.1649A>C | p.D550A | nonsynonymous SNV | 0.28856 |
| P69 | EMSY | NM_001300943 | c.922G>A | p.V308I | nonsynonymous SNV | 0.01171 |
| P61 | ENG | NM_000118 | c.235G>A | p.E79K | nonsynonymous SNV | 0.32342 |
| P24 | ENG | NM_000118 | c.683C>T | p.S228L | nonsynonymous SNV | 0.16236 |
| P172 | ENG | NM_000118 | c.1190A>T | p.E397V | nonsynonymous SNV | 0.03696 |
| P173 | EP300 | NM_001429 | c.6259C>G | p.Q2087E | nonsynonymous SNV | 0.02044 |
| P95 | EP300 | NM_001429 | c.2301G>T | p.Q767H | nonsynonymous SNV | 0.01028 |
| P123 | EP300 | NM_001429 | c.5480delT | p.M1827fs | frameshift deletion | 0.09299 |
| P48 | EP300 | NM_001429 | c.1706_1707insACAGTGGCAAC | p.K569fs | frameshift insertion | 0.19591 |
| P40 | EP300 | - | - | - | loss | 0.97 |
| P49 | EP300 | - | - | - | loss | 1 |
| P18 | EPAS1 | NM_001430 | c.745A>T | p.S249C | nonsynonymous SNV | 0.07586 |
| P19 | EPCAM | NM_002354 | c.727G>C | p.D243H | nonsynonymous SNV | 0.02491 |
| P53 | EPCAM | - | - | - | loss | 0.59 |
| P57 | EPHA3 | NM_005233 | c.413A>G | p.H138R | nonsynonymous SNV | 0.18338 |
| P174 | EPHA3 | NM_005233 | c.2755T>A | p.W919R | nonsynonymous SNV | 0.01055 |
| P24 | EPHA3 | NM_005233 | c.1120G>A | p.E374K | nonsynonymous SNV | 0.1115 |
| P243 | EPHA3 | NM_005233 | c.426delG | p.K142fs | frameshift deletion | 0.09371 |
| P54 | EPHA5 | NM_004439 | c.2726A>T | p.Q909L | nonsynonymous SNV | 0.25164 |
| P93 | EPHA5 | NM_004439 | c.2014G>A | p.A672T | nonsynonymous SNV | 0.08048 |
| P96 | EPHA5 | NM_004439 | c.1895A>T | p.E632V | nonsynonymous SNV | 0.04146 |
| P61 | EPHA5 | NM_004439 | c.1288G>A | p.D430N | nonsynonymous SNV | 0.04727 |
| P175 | EPHA5 | NM_004439 | c.2351T>A | p.I784N | nonsynonymous SNV | 0.01437 |
| P176 | EPHA7 | NM_001288629 | c.2615G>A | p.R872H | nonsynonymous SNV | 0.18311 |
| P128 | EPHA7 | NM_001288629 | c.2809G>C | p.D937H | nonsynonymous SNV | 0.09871 |
| P100 | EPHA7 | NM_001288629 | c.1784-2A>G | - | - | 0.01228 |
| P191 | EPHB4 | NM_004444 | c.274_275insGCGCT | p.F92fs | frameshift insertion | 0.02052 |
| P177 | ERBB2 | NM_004448 | c.2078A>C | p.E693A | nonsynonymous SNV | 0.14922 |
| P178 | ERBB2 | NM_004448 | c.2683C>T | p.L895F | nonsynonymous SNV | 0.03692 |
| P58 | ERBB2 | - | - | - | gain | 4.09 |
| P140 | ERBB3 | NM_001982 | c.2110T>A | p.F704I | nonsynonymous SNV | 0.21835 |
| P44 | ERBB3 | - | - | - | gain | 5.06 |
| P179 | ERBB4 | NM_005235 | c.259G>A | p.V87M | nonsynonymous SNV | 0.37588 |
| P167 | ERBB4 | NM_005235 | c.2071G>A | p.E691K | nonsynonymous SNV | 0.04721 |
| P167 | ERBB4 | NM_005235 | c.1369A>T | p.N457Y | nonsynonymous SNV | 0.1129 |
| P61 | ERBB4 | NM_005235 | c.925C>T | p.P309S | nonsynonymous SNV | 0.21228 |
| P180 | ERBB4 | NM_005235 | c.3791C>A | p.T1264K | nonsynonymous SNV | 0.18902 |
| P88 | ERBB4 | NM_005235 | c.2152C>A | p.L718M | nonsynonymous SNV | 0.2426 |
| P135 | ERBB4 | NM_005235 | c.2258T>C | p.L753P | nonsynonymous SNV | 0.29126 |
| P194 | ERBB4 | NM_005235 | c.80C>A | p.S27X | stopgain | 0.01078 |
| P166 | ERCC2 | NM_000400 | c.898G>A | p.E300K | nonsynonymous SNV | 0.04493 |
| P62 | ERCC2 | - | - | - | gain | 4.822 |
| P86 | ERCC3 | NM_000122 | c.698C>A | p.S233Y | nonsynonymous SNV | 0.15449 |
| P93 | ERCC3 | NM_000122 | c.1156G>A | p.D386N | nonsynonymous SNV | 0.09768 |
| P143 | ERCC4 | NM_005236 | c.962G>C | p.G321A | nonsynonymous SNV | 0.45587 |
| P61 | ERCC4 | NM_005236 | c.1001C>T | p.S334L | nonsynonymous SNV | 0.04785 |
| P27 | ERCC4 | NM_005236 | c.2219G>A | p.R740H | nonsynonymous SNV | 0.57692 |
| P110 | ERF | NM_006494 | c.70T>A | p.S24T | nonsynonymous SNV | 0.05983 |
| P24 | ERF | NM_006494 | c.1125C>G | p.F375L | nonsynonymous SNV | 0.1358 |
| P166 | ERICH3 | NM_001002912 | c.3913delG | p.E1305fs | frameshift deletion | 0.05855 |
| P113 | ESR1 | NM_001122740 | c.1359G>T | p.L453F | nonsynonymous SNV | 0.07566 |
| P6 | ETV1 | NM_004956 | c.587C>G | p.S196C | nonsynonymous SNV | 0.49189 |
| P111 | ETV1 | NM_004956 | c.1285A>T | p.N429Y | nonsynonymous SNV | 0.21028 |
| P181 | ETV1 | NM_004956 | c.414dupC | p.T139fs | frameshift insertion | 0.01305 |
| P76 | ETV4 | NM_001079675 | c.399delC | p.P133fs | frameshift deletion | 0.01082 |
| P15 | ETV4 | NM_001079675 | c.88G>T | p.E30X | stopgain | 0.01553 |
| P96 | ETV5 | NM_004454 | c.625G>A | p.E209K | nonsynonymous SNV | 0.0475 |
| P167 | ETV6 | NM_001987 | c.218C>T | p.A73V | nonsynonymous SNV | 0.04975 |
| P155 | ETV6 | NM_001987 | c.35A>T | p.Q12L | nonsynonymous SNV | 0.23521 |
| P144 | ETV6 | NM_001987 | c.862A>T | p.K288X | stopgain | 0.40865 |
| P226 | EYA4 | NM_172103 | c.302-2A>T | - | - | 0.38571 |
| P154 | EZH1 | NM_001321079 | c.2057G>A | p.R686Q | nonsynonymous SNV | 0.01562 |
| P181 | EZH1 | NM_001321079 | c.1028G>T | p.C343F | nonsynonymous SNV | 0.34666 |
| P130 | EZH1 | NM_001321079 | c.2036_2036del | p.D679fs | frameshift deletion | 0.02746 |
| P144 | EZH2 | NM_001203247 | c.168A>T | p.L56F | nonsynonymous SNV | 0.28341 |
| P63 | FAM175A | - | - | - | loss | 0.395 |
| P134 | FANCA | NM_000135 | c.4156C>G | p.L1386V | nonsynonymous SNV | 0.13003 |
| P117 | FANCA | NM_000135 | c.241_242del | p.D81fs | frameshift deletion | 0.15294 |
| P171 | FANCA | NM_000135 | c.1162A>T | p.R388X | stopgain | 0.28143 |
| P89 | FANCC | NM_000136 | c.1000C>T | p.R334W | nonsynonymous SNV | 0.04708 |
| P189 | FANCC | NM_000136 | c.275G>A | p.W92X | stopgain | 0.01184 |
| P161 | FANCC | NM_000136 | c.686+1G>T | - | - | 0.10877 |
| P182 | FANCD2 | NM_033084 | c.2365A>T | p.T789S | nonsynonymous SNV | 0.02512 |
| P167 | FANCD2 | NM_033084 | c.1654C>T | p.Q552X | stopgain | 0.03134 |
| P57 | FANCF | NM_022725 | c.934C>T | p.Q312X | stopgain | 0.09081 |
| P166 | FANCI | NM_001113378 | c.3595dupA | p.V1198fs | frameshift insertion | 0.05362 |
| P63 | FANCM | - | - | - | loss | 0.17 |
| P6 | FANCM | - | - | - | loss | 0.429 |
| P13 | FANCM | - | - | - | loss | 0.436 |
| P5 | FANCM | - | - | - | loss | 0.095 |
| P68 | FANCM | - | - | - | loss | 0.065 |
| P91 | FAS | NM_001320619 | c.298A>T | p.K100X | stopgain | 0.06533 |
| P75 | FAT1 | NM_005245 | c.3770G>A | p.R1257Q | nonsynonymous SNV | 0.47235 |
| P42 | FAT1 | NM_005245 | c.13652C>G | p.A4551G | nonsynonymous SNV | 0.01106 |
| P180 | FAT1 | NM_005245 | c.13424G>A | p.G4475D | nonsynonymous SNV | 0.17981 |
| P78 | FAT1 | NM_005245 | c.7957G>A | p.G2653S | nonsynonymous SNV | 0.02378 |
| P78 | FAT1 | NM_005245 | c.755A>C | p.E252A | nonsynonymous SNV | 0.02182 |
| P97 | FAT1 | NM_005245 | c.11029G>A | p.D3677N | nonsynonymous SNV | 0.49009 |
| P5 | FAT1 | NM_005245 | c.6695A>C | p.N2232T | nonsynonymous SNV | 0.56052 |
| P102 | FAT1 | NM_005245 | c.3337G>A | p.D1113N | nonsynonymous SNV | 0.01147 |
| P130 | FAT1 | NM_005245 | c.10156G>T | p.D3386Y | nonsynonymous SNV | 0.12216 |
| P183 | FAT1 | NM_005245 | c.10625C>T | p.P3542L | nonsynonymous SNV | 0.08548 |
| P142 | FAT1 | NM_005245 | c.3492delT | p.F1164fs | frameshift deletion | 0.10664 |
| P205 | FAT1 | NM_005245 | c.6378delC | p.H2126fs | frameshift deletion | 0.01053 |
| P144 | FAT1 | NM_005245 | c.7139_7143del | p.V2380fs | frameshift deletion | 0.39315 |
| P38 | FAT3 | NM_001008781 | c.8201C>T | p.P2734L | nonsynonymous SNV | 0.02262 |
| P151 | FAT3 | NM_001008781 | c.7432A>T | p.S2478C | nonsynonymous SNV | 0.10063 |
| P184 | FAT3 | NM_001008781 | c.7723C>A | p.L2575I | nonsynonymous SNV | 0.21915 |
| P185 | FBXW7 | NM_033632 | c.1138G>C | p.D380H | nonsynonymous SNV | 0.22485 |
| P81 | FGF10 | NM_004465 | c.472G>A | p.E158K | nonsynonymous SNV | 0.30852 |
| P19 | FGF14 | NM_001321946 | c.424A>T | p.S142C | nonsynonymous SNV | 0.0355 |
| P36 | FGF19 | NM_005117 | c.307A>G | p.M103V | nonsynonymous SNV | 0.22901 |
| P52 | FGF19 | - | - | - | gain | 4 |
| P24 | FGF23 | NM_020638 | c.17T>A | p.L6H | nonsynonymous SNV | 0.17273 |
| P181 | FGF4 | NM_002007 | c.473A>C | p.K158T | nonsynonymous SNV | 0.16839 |
| P153 | FGFR1 | NM_023110 | c.1396C>T | p.P466S | nonsynonymous SNV | 0.11683 |
| P41 | FGFR1 | - | - | - | gain | 4.97 |
| P48 | FGFR1 | - | - | - | gain | 4.08 |
| P130 | FGFR2 | NM_000141 | c.1292C>T | p.S431L | nonsynonymous SNV | 0.06818 |
| P81 | FGFR3 | NM_000142 | c.1445G>A | p.C482Y | nonsynonymous SNV | 0.05435 |
| P139 | FGFR3 | NM_000142 | c.1729T>C | p.Y577H | nonsynonymous SNV | 0.09024 |
| P92 | FGFR3 | NM_000142 | c.2186A>T | p.E729V | nonsynonymous SNV | 0.25197 |
| P44 | FGFR3 | NM_000142 | c.746C>G | p.S249C | nonsynonymous SNV | 0.0148 |
| P44 | FGFR3 | NM_000142 | c.1948A>G | p.K650E | nonsynonymous SNV | 0.02292 |
| P232 | FGFR3 | NM_000142 | c.1719dupG | p.P573fs | frameshift insertion | 0.01022 |
| P44 | FGFR3 | - | - | - | gain | 4.9 |
| P62 | FGFR3 | - | - | - | gain | 5.109 |
| P143 | FGFR4 | NM_213647 | c.668C>T | p.T223I | nonsynonymous SNV | 0.53388 |
| P109 | FGFR4 | NM_213647 | c.44C>T | p.P15L | nonsynonymous SNV | 0.16874 |
| P186 | FGFR4 | NM_213647 | c.1838T>A | p.L613Q | nonsynonymous SNV | 0.02174 |
| P187 | FGFR4 | NM_213647 | c.1409G>A | p.G470E | nonsynonymous SNV | 0.08517 |
| P144 | FH | NM_000143 | c.1400A>T | p.K467M | nonsynonymous SNV | 0.38478 |
| P257 | FH | NM_000143 | c.238A>T | p.K80X | stopgain | 0.31293 |
| P257 | FH | NM_000143 | c.379-2A>T | - | - | 0.35169 |
| P226 | FIGN | NM_001321825 | c.853C>T | p.Q285X | stopgain | 0.43671 |
| P24 | FLCN | NM_144997 | c.301G>A | p.E101K | nonsynonymous SNV | 0.01931 |
| P98 | FLCN | NM_144997 | c.247G>A | p.E83K | nonsynonymous SNV | 0.01348 |
| P46 | FLT1 | NM_002019 | c.3471G>T | p.L1157F | nonsynonymous SNV | 0.08964 |
| P50 | FLT1 | - | - | - | gain | 5.25 |
| P188 | FLT3 | NM_004119 | c.1721G>T | p.S574I | nonsynonymous SNV | 0.01358 |
| P189 | FLT4 | NM_182925 | c.1228C>T | p.R410C | nonsynonymous SNV | 0.01023 |
| P85 | FLT4 | NM_182925 | c.89delC | p.P30fs | frameshift deletion | 0.01087 |
| P114 | FLT4 | NM_182925 | c.89delC | p.P30fs | frameshift deletion | 0.01145 |
| P229 | FLT4 | NM_182925 | c.89delC | p.P30fs | frameshift deletion | 0.01109 |
| P5 | FOXA1 | NM_004496 | c.77A>G | p.Y26C | nonsynonymous SNV | 0.55695 |
| P44 | FOXA1 | NM_004496 | c.1396A>G | p.R466G | nonsynonymous SNV | 0.40075 |
| P15 | FOXA1 | NM_004496 | c.1281_1284del | p.S427fs | frameshift deletion | 0.0189 |
| P66 | FOXA1 | - | - | - | gain | 7.14 |
| P174 | FOXL2 | NM_023067 | c.500T>A | p.F167Y | nonsynonymous SNV | 0.01115 |
| P152 | FOXO1 | NM_002015 | c.1105G>A | p.E369K | nonsynonymous SNV | 0.01236 |
| P6 | FOXP1 | NM_001244810 | c.1559A>G | p.H520R | nonsynonymous SNV | 0.24956 |
| P42 | FOXP1 | NM_032682 | c.1382A>G | p.N461S | nonsynonymous SNV | 0.01471 |
| P48 | FOXP1 | NM_032682 | c.877C>A | p.H293N | nonsynonymous SNV | 0.22257 |
| P33 | FRS2 | NM_001278354 | c.155G>C | p.R52P | nonsynonymous SNV | 0.27873 |
| P91 | FRS2 | NM_001278354 | c.1483A>T | p.T495S | nonsynonymous SNV | 0.05639 |
| P2 | FRS2 | NM_001278354 | c.1137dupA | p.L379fs | frameshift insertion | 0.02455 |
| P190 | FUBP1 | NM_003902 | c.917G>A | p.G306D | nonsynonymous SNV | 0.10291 |
| P93 | FYN | NM_002037 | c.721C>T | p.R241C | nonsynonymous SNV | 0.11606 |
| P147 | GABRA6 | NM_000811 | c.349delT | p.F117fs | frameshift deletion | 0.01026 |
| P166 | GATA1 | NM_002049 | c.310T>A | p.Y104N | nonsynonymous SNV | 0.04965 |
| P81 | GATA3 | NM_002051 | c.1321G>A | p.A441T | nonsynonymous SNV | 0.01753 |
| P191 | GATA3 | NM_002051 | c.1321G>A | p.A441T | nonsynonymous SNV | 0.30252 |
| P128 | GATA3 | NM_002051 | c.25C>T | p.R9C | nonsynonymous SNV | 0.2369 |
| P99 | GATA4 | NM_001308094 | c.215C>T | p.T72I | nonsynonymous SNV | 0.01202 |
| P155 | GATA6 | NM_005257 | c.523_540del | p.175_180del | nonframeshift deletion | 0.01007 |
| P183 | GEN1 | NM_182625 | c.1106A>G | p.H369R | nonsynonymous SNV | 0.0748 |
| P100 | GLI1 | NM_005269 | c.1695delC | p.F565fs | frameshift deletion | 0.01246 |
| P91 | GNA13 | NM_006572 | c.908dupT | p.V303fs | frameshift insertion | 0.18872 |
| P192 | GNAQ | NM_002072 | c.776T>C | p.I259T | nonsynonymous SNV | 0.02304 |
| P94 | GNAQ | NM_002072 | c.175A>C | p.M59L | nonsynonymous SNV | 0.02232 |
| P193 | GNAQ | NM_002072 | c.175A>C | p.M59L | nonsynonymous SNV | 0.02667 |
| P79 | GNAQ | NM_002072 | c.175A>C | p.M59L | nonsynonymous SNV | 0.02929 |
| P130 | GNAQ | NM_002072 | c.175A>C | p.M59L | nonsynonymous SNV | 0.01752 |
| P6 | GNAQ | NM_002072 | c.303C>A | p.Y101X | stopgain | 0.01693 |
| P2 | GNAQ | NM_002072 | c.303C>A | p.Y101X | stopgain | 0.0409 |
| P143 | GNAQ | NM_002072 | c.303C>A | p.Y101X | stopgain | 0.02573 |
| P197 | GNAQ | NM_002072 | c.303C>A | p.Y101X | stopgain | 0.04648 |
| P75 | GNAQ | NM_002072 | c.303C>A | p.Y101X | stopgain | 0.05492 |
| P125 | GNAQ | NM_002072 | c.303C>A | p.Y101X | stopgain | 0.02694 |
| P190 | GNAQ | NM_002072 | c.303C>A | p.Y101X | stopgain | 0.01211 |
| P255 | GNAQ | NM_002072 | c.303C>A | p.Y101X | stopgain | 0.06507 |
| P132 | GNAQ | NM_002072 | c.303C>A | p.Y101X | stopgain | 0.01507 |
| P28 | GNAQ | NM_002072 | c.303C>A | p.Y101X | stopgain | 0.01516 |
| P104 | GNAQ | NM_002072 | c.303C>A | p.Y101X | stopgain | 0.01832 |
| P258 | GNAQ | NM_002072 | c.303C>A | p.Y101X | stopgain | 0.04431 |
| P167 | GNAQ | NM_002072 | c.303C>A | p.Y101X | stopgain | 0.14815 |
| P199 | GNAQ | NM_002072 | c.303C>A | p.Y101X | stopgain | 0.01194 |
| P60 | GNAQ | NM_002072 | c.303C>A | p.Y101X | stopgain | 0.03995 |
| P236 | GNAQ | NM_002072 | c.303C>A | p.Y101X | stopgain | 0.01187 |
| P219 | GNAQ | NM_002072 | c.303C>A | p.Y101X | stopgain | 0.01569 |
| P215 | GNAQ | NM_002072 | c.303C>A | p.Y101X | stopgain | 0.02434 |
| P251 | GNAQ | NM_002072 | c.303C>A | p.Y101X | stopgain | 0.0295 |
| P248 | GNAQ | NM_002072 | c.303C>A | p.Y101X | stopgain | 0.07483 |
| P97 | GNAQ | NM_002072 | c.303C>A | p.Y101X | stopgain | 0.04013 |
| P5 | GNAQ | NM_002072 | c.303C>A | p.Y101X | stopgain | 0.01632 |
| P89 | GNAQ | NM_002072 | c.303C>A | p.Y101X | stopgain | 0.04483 |
| P79 | GNAQ | NM_002072 | c.303C>A | p.Y101X | stopgain | 0.11316 |
| P27 | GNAQ | NM_002072 | c.303C>A | p.Y101X | stopgain | 0.136 |
| P74 | GNAQ | NM_002072 | c.303C>A | p.Y101X | stopgain | 0.01145 |
| P227 | GNAQ | NM_002072 | c.303C>A | p.Y101X | stopgain | 0.01322 |
| P128 | GNAQ | NM_002072 | c.303C>A | p.Y101X | stopgain | 0.01537 |
| P149 | GNAQ | NM_002072 | c.303C>A | p.Y101X | stopgain | 0.01987 |
| P194 | GNAS | NM_016592 | c.731G>A | p.R244H | nonsynonymous SNV | 0.0191 |
| P213 | GPC3 | NM_001164617 | c.595C>T | p.R199X | stopgain | 0.27978 |
| P46 | GPS2 | NM_004489 | c.499T>C | p.S167P | nonsynonymous SNV | 0.03318 |
| P153 | GPS2 | NM_004489 | c.709C>T | p.Q237X | stopgain | 0.16935 |
| P111 | GRIN2A | NM_000833 | c.1029G>T | p.W343C | nonsynonymous SNV | 0.13248 |
| P74 | GRIN2A | NM_000833 | c.427A>C | p.T143P | nonsynonymous SNV | 0.04673 |
| P172 | GRIN2A | NM_000833 | c.4021dupA | p.S1341fs | frameshift insertion | 0.31008 |
| P144 | GRIN2A | NM_000833 | c.1008-2A>T | - | - | 0.40735 |
| P125 | GRM3 | NM_000840 | c.485G>A | p.R162Q | nonsynonymous SNV | 0.0127 |
| P174 | GRM3 | NM_000840 | c.550T>A | p.Y184N | nonsynonymous SNV | 0.01069 |
| P121 | GRM3 | NM_000840 | c.1446A>C | p.K482N | nonsynonymous SNV | 0.02871 |
| P153 | GRM8 | NM_001127323 | c.1888T>G | p.F630V | nonsynonymous SNV | 0.02826 |
| P25 | GSTT1 | - | - | - | loss | 0.8 |
| P42 | GSTT1 | - | - | - | loss | 0.82 |
| P44 | GSTT1 | - | - | - | loss | 0.8 |
| P46 | GSTT1 | - | - | - | loss | 0.43 |
| P47 | GSTT1 | - | - | - | loss | 0.67 |
| P50 | GSTT1 | - | - | - | loss | 0.56 |
| P66 | GSTT1 | - | - | - | loss | 0.001 |
| P67 | GSTT1 | - | - | - | loss | 0.003 |
| P194 | H3F3B | NM_005324 | c.365C>T | p.P122L | nonsynonymous SNV | 0.03461 |
| P93 | HIST1H1C | NM_005319 | c.512C>T | p.A171V | nonsynonymous SNV | 0.20911 |
| P89 | HIST1H1C | NM_005319 | c.566C>T | p.A189V | nonsynonymous SNV | 0.02437 |
| P195 | HIST1H3A | NM_003529 | c.52C>T | p.R18C | nonsynonymous SNV | 0.13514 |
| P195 | HIST1H3A | NM_003529 | c.51delA | p.P17fs | frameshift deletion | 0.13043 |
| P128 | HIST1H3B | NM_003537 | c.240G>C | p.K80N | nonsynonymous SNV | 0.12615 |
| P72 | HIST1H3D | NM_003530 | c.232G>C | p.D78H | nonsynonymous SNV | 0.07536 |
| P55 | HIST1H3E | NM_003532 | c.239A>C | p.K80T | nonsynonymous SNV | 0.01887 |
| P91 | HIST2H3D | NM_001123375 | c.25C>T | p.R9C | nonsynonymous SNV | 0.01168 |
| P196 | HIST3H3 | NM_003493 | c.115C>A | p.P39T | nonsynonymous SNV | 0.02114 |
| P99 | HLA-A | NM_002116 | c.913A>C | p.T305P | nonsynonymous SNV | 0.0315 |
| P185 | HLA-A | NM_002116 | c.723G>C | p.W241C | nonsynonymous SNV | 0.0991 |
| P197 | HLA-A | NM_002116 | c.620_621insCC | p.D207fs | frameshift insertion | 0.0251 |
| P192 | HLA-A | NM_002116 | c.873_874AG | - | nonframeshift substitution | 0.01546 |
| P158 | HLA-A | NM_002116 | c.873_874AG | - | nonframeshift substitution | 0.01001 |
| P14 | HLA-B | NM_005514 | c.517G>A | p.A173T | nonsynonymous SNV | 0.01025 |
| P124 | HLA-B | NM_005514 | c.1045+1G>T | - | - | 0.01051 |
| P98 | HLA-C | NM_001243042 | c.688C>A | p.L230M | nonsynonymous SNV | 0.59175 |
| P114 | HLA-C | NM_001243042 | c.506G>T | p.R169L | nonsynonymous SNV | 0.01108 |
| P168 | HNF1A | NM_000545 | c.235G>A | p.E79K | nonsynonymous SNV | 0.24897 |
| P15 | HNF1A | NM_000545 | c.502C>T | p.R168C | nonsynonymous SNV | 0.09679 |
| P78 | HRAS | NM_001130442 | c.182A>T | p.Q61L | nonsynonymous SNV | 0.02335 |
| P133 | HRAS | NM_001318054 | c.50C>T | p.A17V | nonsynonymous SNV | 0.51192 |
| P167 | HSP90AA1 | NM_001017963 | c.492G>C | p.E164D | nonsynonymous SNV | 0.14444 |
| P228 | IDH1 | NM_005896 | c.747delG | p.R249fs | frameshift deletion | 0.16252 |
| P154 | IDH2 | NM_002168 | c.514A>G | p.R172G | nonsynonymous SNV | 0.61511 |
| P45 | IFNGR1 | NM_000416 | c.1238A>T | p.D413V | nonsynonymous SNV | 0.02561 |
| P167 | IGF1R | NM_000875 | c.3362A>G | p.D1121G | nonsynonymous SNV | 0.11972 |
| P5 | IGF2R | NM_000876 | c.2449C>G | p.L817V | nonsynonymous SNV | 0.28107 |
| P259 | IGF2R | NM_000876 | c.6458delT | p.I2153fs | frameshift deletion | 0.19472 |
| P197 | IKBKE | NM_014002 | c.547G>A | p.D183N | nonsynonymous SNV | 0.47443 |
| P181 | IKBKE | NM_014002 | c.1544A>T | p.E515V | nonsynonymous SNV | 0.15 |
| P114 | IKBKE | NM_014002 | c.980A>T | p.H327L | nonsynonymous SNV | 0.11502 |
| P14 | IKZF1 | NM_001291839 | c.248A>T | p.E83V | nonsynonymous SNV | 0.07163 |
| P146 | IKZF1 | NM_001291839 | c.712G>A | p.D238N | nonsynonymous SNV | 0.01327 |
| P103 | IKZF1 | NM_001291839 | c.629A>C | p.E210A | nonsynonymous SNV | 0.16425 |
| P192 | INPP4A | NM_001134224 | c.1179-1G>C | - | - | 0.14668 |
| P39 | INPPL1 | NM_001567 | c.3460dupC | p.Q1153fs | frameshift insertion | 0.01006 |
| P83 | INPPL1 | NM_001567 | c.3460dupC | p.Q1153fs | frameshift insertion | 0.01235 |
| P38 | INPPL1 | NM_001567 | c.3460dupC | p.Q1153fs | frameshift insertion | 0.01663 |
| P182 | INPPL1 | NM_001567 | c.3460dupC | p.Q1153fs | frameshift insertion | 0.01114 |
| P28 | INPPL1 | NM_001567 | c.3460dupC | p.Q1153fs | frameshift insertion | 0.01046 |
| P107 | INPPL1 | NM_001567 | c.3460dupC | p.Q1153fs | frameshift insertion | 0.01714 |
| P33 | INPPL1 | NM_001567 | c.3460dupC | p.Q1153fs | frameshift insertion | 0.01513 |
| P61 | INPPL1 | NM_001567 | c.3460dupC | p.Q1153fs | frameshift insertion | 0.01009 |
| P9 | INPPL1 | NM_001567 | c.3460dupC | p.Q1153fs | frameshift insertion | 0.02283 |
| P187 | INPPL1 | NM_001567 | c.3460dupC | p.Q1153fs | frameshift insertion | 0.0107 |
| P260 | INPPL1 | NM_001567 | c.3460dupC | p.Q1153fs | frameshift insertion | 0.01669 |
| P261 | INPPL1 | NM_001567 | c.3460dupC | p.Q1153fs | frameshift insertion | 0.01096 |
| P129 | INPPL1 | NM_001567 | c.3460dupC | p.Q1153fs | frameshift insertion | 0.01235 |
| P228 | INPPL1 | NM_001567 | c.3460dupC | p.Q1153fs | frameshift insertion | 0.01273 |
| P35 | INPPL1 | NM_001567 | c.3460dupC | p.Q1153fs | frameshift insertion | 0.01084 |
| P223 | INPPL1 | NM_001567 | c.3460dupC | p.Q1153fs | frameshift insertion | 0.01304 |
| P218 | INPPL1 | NM_001567 | c.3460dupC | p.Q1153fs | frameshift insertion | 0.01008 |
| P46 | INPPL1 | NM_001567 | c.3460dupC | p.Q1153fs | frameshift insertion | 0.01007 |
| P45 | INPPL1 | NM_001567 | c.3460dupC | p.Q1153fs | frameshift insertion | 0.01044 |
| P262 | INPPL1 | NM_001567 | c.3460dupC | p.Q1153fs | frameshift insertion | 0.01382 |
| P147 | INPPL1 | NM_001567 | c.3460dupC | p.Q1153fs | frameshift insertion | 0.01087 |
| P171 | INPPL1 | NM_001567 | c.3460dupC | p.Q1153fs | frameshift insertion | 0.01031 |
| P61 | INSR | NM_001079817 | c.4096C>T | p.R1366W | nonsynonymous SNV | 0.04992 |
| P198 | INSR | NM_001079817 | c.3199A>G | p.K1067E | nonsynonymous SNV | 0.11679 |
| P177 | IRF4 | NM_001195286 | c.949T>C | p.W317R | nonsynonymous SNV | 0.29367 |
| P54 | IRS1 | NM_005544 | c.512A>T | p.K171M | nonsynonymous SNV | 0.25737 |
| P83 | IRS1 | NM_005544 | c.90C>A | p.F30L | nonsynonymous SNV | 0.25372 |
| P257 | IRS1 | NM_005544 | c.2447dupT | p.L816fs | frameshift insertion | 0.01308 |
| P199 | IRS2 | NM_003749 | c.1919A>T | p.H640L | nonsynonymous SNV | 0.34676 |
| P162 | IRS2 | NM_003749 | c.3382G>T | p.D1128Y | nonsynonymous SNV | 0.02101 |
| P200 | IRS2 | NM_003749 | c.1925G>T | p.S642I | nonsynonymous SNV | 0.17324 |
| P201 | JAK1 | NM_002227 | c.2489A>G | p.Y830C | nonsynonymous SNV | 0.05906 |
| P140 | JAK1 | NM_002227 | c.335A>T | p.Y112F | nonsynonymous SNV | 0.04331 |
| P149 | JAK1 | NM_002227 | c.1322A>G | p.H441R | nonsynonymous SNV | 0.01451 |
| P179 | JAK2 | NM_004972 | c.2185A>T | p.N729Y | nonsynonymous SNV | 0.40243 |
| P63 | JAK2 | - | - | - | loss | 0.4 |
| P5 | JAK2 | - | - | - | loss | 0.15 |
| P89 | JAK3 | NM_000215 | c.3199C>T | p.P1067S | nonsynonymous SNV | 0.05 |
| P89 | JAK3 | NM_000215 | c.2062A>T | p.I688F | nonsynonymous SNV | 0.03971 |
| P202 | JAK3 | NM_000215 | c.97C>T | p.R33W | nonsynonymous SNV | 0.01148 |
| P114 | JAK3 | NM_000215 | c.1842delA | p.K614fs | frameshift deletion | 0.10662 |
| P43 | JAK3 | - | - | - | gain | 4.02 |
| P203 | KAT6A | NM_006766 | c.1310A>G | p.Y437C | nonsynonymous SNV | 0.05419 |
| P204 | KAT6A | NM_006766 | c.4824G>T | p.Q1608H | nonsynonymous SNV | 0.02803 |
| P149 | KAT6A | NM_006766 | c.4645G>T | p.G1549C | nonsynonymous SNV | 0.21702 |
| P204 | KAT6A | NM_006766 | c.4826dupT | p.M1609fs | frameshift insertion | 0.02806 |
| P50 | KCNA4 | NM_002233 | c.841G>C | p.D281H | nonsynonymous SNV | 0.04811 |
| P52 | KCNA4 | NM_002233 | c.404_405insGGAGGAGGAGG | p.E135fs | frameshift insertion | 0.01896 |
| P111 | KDM5A | NM_001042603 | c.4523G>A | p.R1508Q | nonsynonymous SNV | 0.03165 |
| P205 | KDM5A | NM_001042603 | c.172C>A | p.Q58K | nonsynonymous SNV | 0.26827 |
| P134 | KDM5A | NM_001042603 | c.1099G>A | p.G367R | nonsynonymous SNV | 0.1529 |
| P167 | KDM5A | NM_001042603 | c.4491A>T | p.K1497N | nonsynonymous SNV | 0.06044 |
| P206 | KDM5A | NM_001042603 | c.3298G>A | p.E1100K | nonsynonymous SNV | 0.09866 |
| P206 | KDM5A | NM_001042603 | c.3292G>A | p.E1098K | nonsynonymous SNV | 0.05572 |
| P89 | KDM5A | NM_001042603 | c.4420C>A | p.P1474T | nonsynonymous SNV | 0.0385 |
| P189 | KDM5A | NM_001042603 | c.440G>T | p.G147V | nonsynonymous SNV | 0.07104 |
| P72 | KDM5A | NM_001042603 | c.3298G>A | p.E1100K | nonsynonymous SNV | 0.01284 |
| P72 | KDM5A | NM_001042603 | c.3292G>A | p.E1098K | nonsynonymous SNV | 0.01764 |
| P140 | KDM5C | NM_004187 | c.1798G>T | p.A600S | nonsynonymous SNV | 0.0625 |
| P207 | KDM5C | NM_004187 | c.1550A>G | p.D517G | nonsynonymous SNV | 0.35422 |
| P204 | KDM5C | NM_004187 | c.1580A>C | p.H527P | nonsynonymous SNV | 0.02643 |
| P204 | KDM5C | NM_004187 | c.71T>G | p.F24C | nonsynonymous SNV | 0.03279 |
| P177 | KDM5C | NM_004187 | c.1270G>T | p.E424X | stopgain | 0.46347 |
| P140 | KDM5C | NM_004187 | c.1799delC | p.A600fs | frameshift deletion | 0.06309 |
| P49 | KDM5C | NM_004187 | c.3805_3806del | p.P1269fs | frameshift deletion | 0.47685 |
| P32 | KDM5C | NM_004187 | c.4147C>T | p.Q1383X | stopgain | 0.32335 |
| P50 | KDM5C | NM_004187 | c.2010_2011del | p.M670fs | frameshift deletion | 0.22575 |
| P235 | KDM5C | NM_004187 | c.3187delG | p.V1063fs | frameshift deletion | 0.46457 |
| P216 | KDM5C | NM_004187 | c.33_45del | p.P11fs | frameshift deletion | 0.23743 |
| P237 | KDM5C | NM_004187 | c.2851C>T | p.R951X | stopgain | 0.1082 |
| P114 | KDM5C | NM_004187 | c.2062_2074del | p.G688fs | frameshift deletion | 0.25654 |
| P31 | KDM5C | NM_004187 | c.2822C>A | p.S941X | stopgain | 0.60876 |
| P149 | KDM5C | NM_004187 | c.3433dupT | p.S1145fs | frameshift insertion | 0.46349 |
| P77 | KDM6A | NM_021140 | c.598T>A | p.C200S | nonsynonymous SNV | 0.01578 |
| P44 | KDM6A | NM_021140 | c.3125C>A | p.S1042X | stopgain | 0.5764 |
| P229 | KDM6A | NM_021140 | c.3338dupT | p.V1113fs | frameshift insertion | 0.66667 |
| P69 | KDR | NM_002253 | c.1091G>T | p.W364L | nonsynonymous SNV | 0.01591 |
| P89 | KDR | NM_002253 | c.719A>C | p.E240A | nonsynonymous SNV | 0.01903 |
| P121 | KDR | NM_002253 | c.1409C>T | p.P470L | nonsynonymous SNV | 0.18572 |
| P39 | KDR | - | - | - | gain | 4.08 |
| P5 | KDR | - | - | - | gain | 10.526 |
| P58 | KEAP1 | NM_203500 | c.190T>A | p.F64I | nonsynonymous SNV | 0.14402 |
| P10 | KEL | NM_000420 | c.322T>A | p.C108S | nonsynonymous SNV | 0.32577 |
| P81 | KIF2B | NM_032559 | c.769C>T | p.R257C | nonsynonymous SNV | 0.01543 |
| P208 | KIT | NM_000222 | c.1588G>A | p.V530I | nonsynonymous SNV | 0.08477 |
| P68 | KIT | NM_000222 | c.2078A>T | p.K693M | nonsynonymous SNV | 0.38367 |
| P129 | KLF4 | NM_001314052 | c.1195C>A | p.P399T | nonsynonymous SNV | 0.01488 |
| P131 | KLF4 | NM_004235 | c.332delA | p.N111fs | frameshift deletion | 0.08481 |
| P209 | KLHL1 | NM_020866 | c.170T>A | p.L57H | nonsynonymous SNV | 0.01464 |
| P197 | KMT2A | NM_001197104 | c.11560G>A | p.A3854T | nonsynonymous SNV | 0.45667 |
| P61 | KMT2A | NM_001197104 | c.10351G>A | p.E3451K | nonsynonymous SNV | 0.0502 |
| P170 | KMT2A | NM_001197104 | c.5940T>A | p.H1980Q | nonsynonymous SNV | 0.12736 |
| P89 | KMT2A | NM_001197104 | c.9391G>A | p.G3131S | nonsynonymous SNV | 0.03342 |
| P39 | KMT2A | NM_001197104 | c.167delC | p.S56fs | frameshift deletion | 0.01058 |
| P38 | KMT2A | NM_001197104 | c.167delC | p.S56fs | frameshift deletion | 0.01256 |
| P107 | KMT2A | NM_001197104 | c.2312delC | p.T771fs | frameshift deletion | 0.01004 |
| P252 | KMT2A | NM_001197104 | c.167delC | p.S56fs | frameshift deletion | 0.01097 |
| P86 | KMT2B | NM_014727 | c.794G>A | p.S265N | nonsynonymous SNV | 0.15635 |
| P14 | KMT2B | NM_014727 | c.4522T>C | p.F1508L | nonsynonymous SNV | 0.08661 |
| P5 | KMT2B | NM_014727 | c.884G>A | p.R295H | nonsynonymous SNV | 0.38372 |
| P130 | KMT2B | NM_014727 | c.4223C>T | p.A1408V | nonsynonymous SNV | 0.02949 |
| P133 | KMT2B | NM_014727 | c.6779T>C | p.L2260P | nonsynonymous SNV | 0.01143 |
| P189 | KMT2B | NM_014727 | c.6080A>T | p.E2027V | nonsynonymous SNV | 0.09838 |
| P44 | KMT2B | NM_014727 | c.6692C>G | p.S2231C | nonsynonymous SNV | 0.29102 |
| P1 | KMT2B | NM_014727 | c.3297delC | p.G1099fs | frameshift deletion | 0.01147 |
| P210 | KMT2B | NM_014727 | c.515delC | p.T172fs | frameshift deletion | 0.01019 |
| P209 | KMT2B | NM_014727 | c.2318delA | p.E773fs | frameshift deletion | 0.01676 |
| P15 | KMT2B | NM_014727 | c.5649delC | p.G1883fs | frameshift deletion | 0.22782 |
| P185 | KMT2B | NM_014727 | c.5665+1G>C | - | - | 0.20617 |
| P125 | KMT2C | NM_170606 | c.13807G>A | p.E4603K | nonsynonymous SNV | 0.02 |
| P93 | KMT2C | NM_170606 | c.7171A>C | p.I2391L | nonsynonymous SNV | 0.09044 |
| P108 | KMT2C | NM_170606 | c.7058C>G | p.S2353C | nonsynonymous SNV | 0.20268 |
| P210 | KMT2C | NM_170606 | c.13750C>T | p.R4584W | nonsynonymous SNV | 0.06966 |
| P129 | KMT2C | NM_170606 | c.2189C>A | p.S730Y | nonsynonymous SNV | 0.01342 |
| P122 | KMT2C | NM_170606 | c.13476A>C | p.Q4492H | nonsynonymous SNV | 0.02185 |
| P15 | KMT2C | NM_170606 | c.4264G>A | p.G1422R | nonsynonymous SNV | 0.2422 |
| P105 | KMT2C | NM_170606 | c.244G>T | p.E82X | stopgain | 0.16515 |
| P156 | KMT2C | NM_170606 | c.9310delC | p.L3104fs | frameshift deletion | 0.05284 |
| P228 | KMT2C | NM_170606 | c.7921_7924del | p.S2641fs | frameshift deletion | 0.08319 |
| P119 | KMT2C | NM_170606 | c.1645G>T | p.E549X | stopgain | 0.15385 |
| P205 | KMT2C | NM_170606 | c.849+1G>C | - | - | 0.45202 |
| P77 | KMT2C | NM_170606 | c.3323+1G>A | - | - | 0.04 |
| P235 | KMT2C | NM_170606 | c.3323+1G>A | - | - | 0.05303 |
| P248 | KMT2C | NM_170606 | c.3323+1G>A | - | - | 0.06452 |
| P177 | KMT2D | NM_003482 | c.2372A>T | p.Q791L | nonsynonymous SNV | 0.01875 |
| P140 | KMT2D | NM_003482 | c.5551G>C | p.V1851L | nonsynonymous SNV | 0.17353 |
| P96 | KMT2D | NM_003482 | c.16442G>A | p.C5481Y | nonsynonymous SNV | 0.08818 |
| P89 | KMT2D | NM_003482 | c.6284G>A | p.R2095H | nonsynonymous SNV | 0.02788 |
| P79 | KMT2D | NM_003482 | c.6682A>G | p.T2228A | nonsynonymous SNV | 0.54056 |
| P211 | KMT2D | NM_003482 | c.12101C>G | p.S4034C | nonsynonymous SNV | 0.22927 |
| P212 | KMT2D | NM_003482 | c.6983A>G | p.K2328R | nonsynonymous SNV | 0.60923 |
| P213 | KMT2D | NM_003482 | c.16111G>A | p.E5371K | nonsynonymous SNV | 0.01826 |
| P21 | KMT2D | NM_003482 | c.6385C>G | p.P2129A | nonsynonymous SNV | 0.01139 |
| P15 | KMT2D | NM_003482 | c.12203C>T | p.S4068L | nonsynonymous SNV | 0.3112 |
| P33 | KMT2D | NM_003482 | c.12688C>T | p.Q4230X | stopgain | 0.65928 |
| P244 | KMT2D | NM_003482 | c.3622_3623del | p.I1208fs | frameshift deletion | 0.23503 |
| P78 | KMT2D | NM_003482 | c.11377C>T | p.Q3793X | stopgain | 0.01277 |
| P72 | KMT2D | NM_003482 | c.1940delC | p.P647fs | frameshift deletion | 0.01868 |
| P137 | KMT2D | NM_003482 | c.16161_16164del | p.Q5387fs | frameshift deletion | 0.77273 |
| P15 | KMT2D | NM_003482 | c.12019C>T | p.Q4007X | stopgain | 0.32831 |
| P15 | KMT2D | NM_003482 | c.6773delC | p.P2258fs | frameshift deletion | 0.41711 |
| P33 | KMT2D | NM_003482 | c.16489_16491del | p.5497_5497del | nonframeshift deletion | 0.30877 |
| P167 | KMT2D | NM_003482 | c.2250_2276del | p.750_759del | nonframeshift deletion | 0.03435 |
| P119 | KMT2D | NM_003482 | c.2250_2276del | p.750_759del | nonframeshift deletion | 0.02304 |
| P56 | KMT5A | NM_001324506 | c.525dupC | p.C175fs | frameshift insertion | 0.01164 |
| P235 | KMT5A | NM_001324506 | c.218_219del | p.L73fs | frameshift deletion | 0.04023 |
| P181 | KMT5A | NM_001324506 | c.525dupC | p.C175fs | frameshift insertion | 0.0284 |
| P236 | KMT5A | NM_001324506 | c.525dupC | p.C175fs | frameshift insertion | 0.02651 |
| P90 | KMT5A | NM_001324506 | c.525dupC | p.C175fs | frameshift insertion | 0.01142 |
| P128 | KMT5A | NM_001324506 | c.525dupC | p.C175fs | frameshift insertion | 0.0156 |
| P218 | KMT5A | NM_001324506 | c.525dupC | p.C175fs | frameshift insertion | 0.01347 |
| P148 | KMT5A | NM_001324506 | c.525dupC | p.C175fs | frameshift insertion | 0.01677 |
| P188 | KMT5A | NM_001324506 | c.525dupC | p.C175fs | frameshift insertion | 0.01466 |
| P165 | KMT5A | NM_001324506 | c.525dupC | p.C175fs | frameshift insertion | 0.01605 |
| P182 | KRAS | NM_033360 | c.35G>T | p.G12V | nonsynonymous SNV | 0.02721 |
| P214 | KRAS | NM_033360 | c.35G>A | p.G12D | nonsynonymous SNV | 0.01493 |
| P81 | LATS1 | NM_004690 | c.710C>A | p.P237Q | nonsynonymous SNV | 0.01042 |
| P79 | LATS1 | NM_004690 | c.710C>A | p.P237Q | nonsynonymous SNV | 0.24699 |
| P152 | LATS1 | NM_004690 | c.1820A>C | p.K607T | nonsynonymous SNV | 0.1862 |
| P44 | LATS1 | NM_004690 | c.2744A>T | p.N915I | nonsynonymous SNV | 0.35708 |
| P15 | LATS1 | NM_004690 | c.2124A>G | p.I708M | nonsynonymous SNV | 0.25208 |
| P161 | LATS1 | NM_004690 | c.2770C>T | p.R924X | stopgain | 0.08303 |
| P24 | LATS1 | NM_004690 | c.859C>T | p.R287X | stopgain | 0.35764 |
| P16 | LATS2 | NM_014572 | c.2459A>T | p.H820L | nonsynonymous SNV | 0.23282 |
| P79 | LATS2 | NM_014572 | c.2395A>G | p.I799V | nonsynonymous SNV | 0.22639 |
| P205 | LATS2 | NM_014572 | c.3206_3207insT | p.S1069fs | frameshift insertion | 0.29863 |
| P37 | LATS2 | NM_014572 | c.854delG | p.G285fs | frameshift deletion | 0.07224 |
| P37 | LATS2 | NM_014572 | c.696delC | p.P232fs | frameshift deletion | 0.08181 |
| P137 | LRFN5 | NM_001346173 | c.665T>C | p.L222P | nonsynonymous SNV | 0.41572 |
| P7 | LRP1B | NM_018557 | c.5671G>A | p.G1891R | nonsynonymous SNV | 0.08213 |
| P54 | LRP1B | NM_018557 | c.11947A>T | p.I3983F | nonsynonymous SNV | 0.01023 |
| P93 | LRP1B | NM_018557 | c.5837A>T | p.D1946V | nonsynonymous SNV | 0.072 |
| P117 | LRP1B | NM_018557 | c.1207A>T | p.N403Y | nonsynonymous SNV | 0.08309 |
| P99 | LRP1B | NM_018557 | c.3289C>T | p.H1097Y | nonsynonymous SNV | 0.23364 |
| P180 | LRP1B | NM_018557 | c.9191G>A | p.R3064Q | nonsynonymous SNV | 0.1633 |
| P215 | LRP1B | NM_018557 | c.1262T>C | p.V421A | nonsynonymous SNV | 0.19656 |
| P216 | LRP1B | NM_018557 | c.9838A>C | p.N3280H | nonsynonymous SNV | 0.05476 |
| P43 | LRP1B | NM_018557 | c.1385A>T | p.Y462F | nonsynonymous SNV | 0.16733 |
| P217 | LRP1B | NM_018557 | c.7127A>G | p.K2376R | nonsynonymous SNV | 0.04277 |
| P121 | LRP1B | NM_018557 | c.9902C>T | p.P3301L | nonsynonymous SNV | 0.16345 |
| P99 | LRP1B | NM_018557 | c.8792T>A | p.L2931X | stopgain | 0.01161 |
| P9 | LRP1B | - | - | - | loss | 0.84 |
| P116 | LRRC4C | NM_001258419 | c.1782C>A | p.H594Q | nonsynonymous SNV | 0.24043 |
| P213 | LRRC4C | NM_001258419 | c.988T>C | p.C330R | nonsynonymous SNV | 0.27518 |
| P124 | LRRTM1 | NM_178839 | c.1018T>A | p.L340M | nonsynonymous SNV | 0.01477 |
| P216 | LRRTM1 | NM_178839 | c.1189G>A | p.G397S | nonsynonymous SNV | 0.01322 |
| P188 | LRRTM4 | NM_001282928 | c.340G>T | p.E114X | stopgain | 0.01082 |
| P218 | LYN | NM_001111097 | c.324G>T | p.W108C | nonsynonymous SNV | 0.2093 |
| P219 | MAF | NM_005360 | c.1183C>A | p.R395S | nonsynonymous SNV | 0.16972 |
| P69 | MAGI2 | NM_001301128 | c.1721C>T | p.T574I | nonsynonymous SNV | 0.1346 |
| P116 | MAGI2 | NM_001301128 | c.3484C>A | p.L1162M | nonsynonymous SNV | 0.21081 |
| P167 | MAGI2 | NM_001301128 | c.325C>T | p.R109C | nonsynonymous SNV | 0.12796 |
| P57 | MALT1 | NM_173844 | c.98T>G | p.L33R | nonsynonymous SNV | 0.08599 |
| P96 | MALT1 | NM_173844 | c.337A>G | p.M113V | nonsynonymous SNV | 0.07846 |
| P158 | MAP2K1 | NM_002755 | c.607G>C | p.E203Q | nonsynonymous SNV | 0.2059 |
| P129 | MAP2K1 | NM_002755 | c.1062A>C | p.Q354H | nonsynonymous SNV | 0.01113 |
| P161 | MAP2K2 | NM_030662 | c.391G>A | p.V131M | nonsynonymous SNV | 0.17618 |
| P158 | MAP3K1 | NM_005921 | c.950A>G | p.Y317C | nonsynonymous SNV | 0.31199 |
| P167 | MAP3K1 | NM_005921 | c.3130C>T | p.P1044S | nonsynonymous SNV | 0.05576 |
| P188 | MAP3K1 | NM_005921 | c.1519A>T | p.S507C | nonsynonymous SNV | 0.01727 |
| P99 | MAP3K1 | NM_005921 | c.3640G>T | p.E1214X | stopgain | 0.08251 |
| P203 | MCL1 | NM_021960 | c.509A>G | p.E170G | nonsynonymous SNV | 0.21339 |
| P197 | MDC1 | NM_014641 | c.4019A>G | p.Q1340R | nonsynonymous SNV | 0.0186 |
| P126 | MDC1 | NM_014641 | c.2077G>T | p.D693Y | nonsynonymous SNV | 0.26895 |
| P129 | MDC1 | NM_014641 | c.395G>T | p.R132L | nonsynonymous SNV | 0.31835 |
| P22 | MDC1 | NM_014641 | c.4019A>G | p.Q1340R | nonsynonymous SNV | 0.0102 |
| P220 | MDC1 | NM_014641 | c.2522A>T | p.E841V | nonsynonymous SNV | 0.13861 |
| P83 | MDH2 | NM_001282403 | c.299C>T | p.A100V | nonsynonymous SNV | 0.0123 |
| P128 | MDH2 | NM_001282404 | c.667G>A | p.G223R | nonsynonymous SNV | 0.10252 |
| P162 | MDH2 | NM_001282403 | c.142C>T | p.P48S | nonsynonymous SNV | 0.13643 |
| P162 | MDM2 | NM_002392 | c.890T>C | p.F297S | nonsynonymous SNV | 0.08138 |
| P153 | MDM2 | NM_002392 | c.1153G>A | p.D385N | nonsynonymous SNV | 0.13011 |
| P111 | MED12 | NM_005120 | c.5165G>A | p.R1722Q | nonsynonymous SNV | 0.01953 |
| P81 | MED12 | NM_005120 | c.3956G>A | p.R1319H | nonsynonymous SNV | 0.04977 |
| P12 | MEN1 | NM_130804 | c.1253T>C | p.L418P | nonsynonymous SNV | 0.36989 |
| P61 | MEN1 | NM_130804 | c.527G>A | p.R176Q | nonsynonymous SNV | 0.03772 |
| P100 | MEN1 | NM_130804 | c.507_511del | p.A169fs | frameshift deletion | 0.20331 |
| P38 | MET | NM_000245 | c.1577G>T | p.C526F | nonsynonymous SNV | 0.01462 |
| P221 | MET | NM_000245 | c.3274G>A | p.V1092I | nonsynonymous SNV | 0.60591 |
| P169 | MET | NM_000245 | c.1154G>A | p.C385Y | nonsynonymous SNV | 0.01744 |
| P26 | MET | NM_000245 | c.4021T>G | p.F1341V | nonsynonymous SNV | 0.01499 |
| P27 | MET | NM_000245 | c.819delT | p.T273fs | frameshift deletion | 0.30584 |
| P175 | MET | NM_001127500 | c.2296_2303del | p.S766fs | frameshift deletion | 0.2455 |
| P57 | MET | - | - | - | gain | 14.64 |
| P2 | MGA | NM_001164273 | c.9082A>G | p.M3028V | nonsynonymous SNV | 0.46603 |
| P222 | MGA | NM_001164273 | c.8907G>A | p.M2969I | nonsynonymous SNV | 0.2 |
| P110 | MGA | NM_001164273 | c.7504C>T | p.L2502F | nonsynonymous SNV | 0.05286 |
| P151 | MGA | NM_001164273 | c.4049A>G | p.K1350R | nonsynonymous SNV | 0.10478 |
| P223 | MGA | NM_001164273 | c.5369A>G | p.Q1790R | nonsynonymous SNV | 0.07837 |
| P15 | MGA | NM_001164273 | c.5315C>T | p.P1772L | nonsynonymous SNV | 0.38798 |
| P24 | MGA | NM_001164273 | c.1378A>T | p.K460X | stopgain | 0.17271 |
| P135 | MGA | NM_001164273 | c.1870A>T | p.K624X | stopgain | 0.28245 |
| P207 | MITF | - | - | - | Fusion | 0.0782 |
| P164 | MITF | - | - | - | Fusion | 0.0548 |
| P223 | MITF | - | - | - | Fusion | 0.1601 |
| P166 | MITF | - | - | - | Fusion | 0.0551 |
| P88 | MITF | - | - | - | Fusion | 0.1104 |
| P121 | MKRN3 | NM_005664 | c.470C>T | p.A157V | nonsynonymous SNV | 0.01272 |
| P118 | MKRN3 | NM_005664 | c.1285G>T | p.E429X | stopgain | 0.09939 |
| P97 | MLH1 | NM_000249 | c.1151T>A | p.V384D | nonsynonymous SNV | 0.67777 |
| P5 | MLH1 | NM_000249 | c.1151T>A | p.V384D | nonsynonymous SNV | 0.2397 |
| P9 | MLH1 | - | - | - | loss | 0.85 |
| P263 | MRE11 | NM_001330347 | c.1976C>G | p.S659X | stopgain | 0.27778 |
| P89 | MRE11 | NM_001330347 | c.1894C>T | p.R632X | stopgain | 0.01389 |
| P63 | MRE11A | - | - | - | loss | 0.149 |
| P5 | MRE11A | - | - | - | loss | 0.028 |
| P93 | MSH2 | NM_000251 | c.23C>T | p.T8M | nonsynonymous SNV | 0.13126 |
| P3 | MSH2 | NM_000251 | c.2666C>T | p.S889F | nonsynonymous SNV | 0.04319 |
| P129 | MSH2 | NM_000251 | c.1168C>T | p.L390F | nonsynonymous SNV | 0.01047 |
| P156 | MSH2 | NM_000251 | c.2783C>A | p.S928X | stopgain | 0.01557 |
| P51 | MSH2 | - | - | - | loss | 0.9 |
| P9 | MSH2 | - | - | - | loss | 0.75 |
| P63 | MSH3 | - | - | - | loss | 0.09 |
| P170 | MSH6 | NM_001281492 | c.3098A>T | p.E1033V | nonsynonymous SNV | 0.01276 |
| P214 | MSH6 | NM_000179 | c.268T>A | p.F90I | nonsynonymous SNV | 0.18483 |
| P103 | MSH6 | NM_001281492 | c.1925G>A | p.R642Q | nonsynonymous SNV | 0.01475 |
| P113 | MSH6 | NM_001281492 | c.3479delT | p.I1160fs | frameshift deletion | 0.22863 |
| P89 | MSH6 | NM_001281492 | c.3675_3676insTTGA | p.T1225fs | frameshift insertion | 0.03369 |
| P185 | MSH6 | NM_001281492 | c.3600delA | p.L1200fs | frameshift deletion | 0.17747 |
| P72 | MSI1 | NM_002442 | c.821C>T | p.A274V | nonsynonymous SNV | 0.13555 |
| P170 | MSI2 | NM_001322250 | c.580A>T | p.M194L | nonsynonymous SNV | 0.16754 |
| P22 | MSI2 | NM_001322250 | c.518G>A | p.G173E | nonsynonymous SNV | 0.01077 |
| P219 | MST1 | NM_020998 | c.1967A>T | p.E656V | nonsynonymous SNV | 0.27852 |
| P118 | MST1 | NM_020998 | c.1421_1422insCC | p.P474fs | frameshift insertion | 0.01241 |
| P258 | MST1R | NM_002447 | c.1186C>T | p.R396X | stopgain | 0.13151 |
| P124 | MTOR | NM_004958 | c.3811G>A | p.A1271T | nonsynonymous SNV | 0.33122 |
| P62 | MTOR | NM_004958 | c.7289G>T | p.R2430M | nonsynonymous SNV | 0.03733 |
| P7 | MTOR | NM_004958 | c.6065T>C | p.L2022P | nonsynonymous SNV | 0.18274 |
| P205 | MTOR | NM_004958 | c.7207G>C | p.V2403L | nonsynonymous SNV | 0.41224 |
| P224 | MTOR | NM_004958 | c.7500T>G | p.I2500M | nonsynonymous SNV | 0.20199 |
| P225 | MTOR | NM_004958 | c.6050T>G | p.I2017S | nonsynonymous SNV | 0.26 |
| P56 | MTOR | NM_004958 | c.7217T>C | p.V2406A | nonsynonymous SNV | 0.03858 |
| P32 | MTOR | NM_004958 | c.3617A>G | p.H1206R | nonsynonymous SNV | 0.15259 |
| P174 | MTOR | NM_004958 | c.7501A>T | p.I2501F | nonsynonymous SNV | 0.06285 |
| P60 | MTOR | NM_004958 | c.7280T>G | p.L2427R | nonsynonymous SNV | 0.2866 |
| P106 | MTOR | NM_004958 | c.6981G>T | p.M2327I | nonsynonymous SNV | 0.21961 |
| P106 | MTOR | NM_004958 | c.5930C>A | p.T1977K | nonsynonymous SNV | 0.07572 |
| P197 | MUC16 | NM_024690 | c.41897G>A | p.R13966K | nonsynonymous SNV | 0.47943 |
| P226 | MUT | NM_000255 | c.998T>C | p.I333T | nonsynonymous SNV | 0.37551 |
| P35 | MYB | NM_005375 | c.371G>T | p.G124V | nonsynonymous SNV | 0.1528 |
| P128 | MYB | NM_005375 | c.1067C>G | p.S356C | nonsynonymous SNV | 0.34335 |
| P33 | MYC | - | - | - | gain | 14.88 |
| P98 | MYCL | NM_001033082 | c.19T>A | p.C7S | nonsynonymous SNV | 0.40136 |
| P154 | MYCN | NM_001293231 | c.92G>C | p.R31P | nonsynonymous SNV | 0.04038 |
| P166 | MYCN | NM_001293231 | c.92G>C | p.R31P | nonsynonymous SNV | 0.03663 |
| P205 | MYCN | NM_001293231 | c.93dupC | p.R31fs | frameshift insertion | 0.02757 |
| P264 | MYD88 | NM_001172566 | c.478T>C | p.X160R | stoploss | 0.01528 |
| P226 | MYL3 | NM_000258 | c.284T>C | p.V95A | nonsynonymous SNV | 0.43723 |
| P102 | MYOD1 | NM_002478 | c.697G>A | p.E233K | nonsynonymous SNV | 0.01415 |
| P7 | NCOA3 | NM_006534 | c.688T>A | p.S230T | nonsynonymous SNV | 0.09963 |
| P149 | NCOA3 | NM_006534 | c.2774C>T | p.S925L | nonsynonymous SNV | 0.21918 |
| P15 | NCOA3 | NM_006534 | c.261A>T | p.K87N | nonsynonymous SNV | 0.03386 |
| P3 | NCOR1 | NM_006311 | c.3338G>A | p.R1113Q | nonsynonymous SNV | 0.07944 |
| P127 | NCOR1 | NM_006311 | c.1899G>C | p.M633I | nonsynonymous SNV | 0.18078 |
| P41 | NCOR1 | NM_006311 | c.611delA | p.K204fs | frameshift deletion | 0.03061 |
| P63 | NCOR1 | - | - | - | loss | 1.472 |
| P136 | NCOR2 | NM_001206654 | c.4546G>A | p.A1516T | nonsynonymous SNV | 0.05353 |
| P27 | NCOR2 | NM_001206654 | c.3581C>T | p.P1194L | nonsynonymous SNV | 0.46231 |
| P184 | NCOR2 | NM_001206654 | c.2369C>A | p.P790H | nonsynonymous SNV | 0.20287 |
| P191 | NCOR2 | NM_001206654 | c.2871delC | p.P957fs | frameshift deletion | 0.01142 |
| P144 | NCOR2 | NM_001206654 | c.3874delC | p.H1292fs | frameshift deletion | 0.0111 |
| P97 | NEGR1 | NM_173808 | c.307delC | p.L103fs | frameshift deletion | 0.01777 |
| P111 | NF1 | NM_001042492 | c.5444A>T | p.Q1815L | nonsynonymous SNV | 0.22425 |
| P93 | NF1 | NM_001042492 | c.1259A>T | p.N420I | nonsynonymous SNV | 0.01235 |
| P56 | NF1 | NM_001042492 | c.5741C>A | p.T1914K | nonsynonymous SNV | 0.16715 |
| P88 | NF1 | NM_001042492 | c.1429T>C | p.F477L | nonsynonymous SNV | 0.20994 |
| P68 | NF1 | NM_001042492 | c.1220A>G | p.H407R | nonsynonymous SNV | 0.37153 |
| P253 | NF1 | NM_001042492 | c.2416delG | p.E806fs | frameshift deletion | 0.23586 |
| P253 | NF1 | NM_001042492 | c.4267G>T | p.E1423X | stopgain | 0.24403 |
| P227 | NF2 | NM_000268 | c.334G>A | p.E112K | nonsynonymous SNV | 0.21512 |
| P253 | NF2 | NM_000268 | c.1161delG | p.K387fs | frameshift deletion | 0.35242 |
| P14 | NF2 | NM_000268 | c.556G>T | p.E186X | stopgain | 0.09289 |
| P265 | NF2 | NM_000268 | c.958C>T | p.Q320X | stopgain | 0.02729 |
| P81 | NF2 | NM_000268 | c.773G>A | p.W258X | stopgain | 0.43902 |
| P9 | NF2 | NM_000268 | c.377delT | p.I126fs | frameshift deletion | 0.38034 |
| P27 | NF2 | NM_000268 | c.348_349insA | p.H116fs | frameshift insertion | 0.55056 |
| P227 | NF2 | NM_000268 | c.337delA | p.I113fs | frameshift deletion | 0.21128 |
| P223 | NF2 | NM_000268 | c.615_624del | p.M205fs | frameshift deletion | 0.05984 |
| P37 | NF2 | NM_000268 | c.352_354del | p.118_118del | nonframeshift deletion | 0.35583 |
| P272 | NF2 | NM_000268 | c.240+2T>C | - | - | 0.76233 |
| P48 | NFE2L2 | NM_006164 | c.239C>A | p.T80K | nonsynonymous SNV | 0.10009 |
| P168 | NKX2-1 | NM_001079668 | c.1118A>T | p.Q373L | nonsynonymous SNV | 0.02068 |
| P39 | NLRC5 | NM_001330552 | c.2769T>G | p.S923R | nonsynonymous SNV | 0.06321 |
| P168 | NLRC5 | NM_001330552 | c.157delA | p.R53fs | frameshift deletion | 0.17483 |
| P149 | NLRP3 | NM_001079821 | c.1847C>T | p.A616V | nonsynonymous SNV | 0.01581 |
| P179 | NLRP3 | NM_001079821 | c.508C>T | p.R170X | stopgain | 0.02943 |
| P50 | NLRP3 | NM_001079821 | c.1123dupG | p.S374fs | frameshift insertion | 0.09108 |
| P2 | NLRP5 | NM_153447 | c.1612G>A | p.G538R | nonsynonymous SNV | 0.48922 |
| P117 | NLRP5 | NM_153447 | c.1691T>A | p.L564Q | nonsynonymous SNV | 0.093 |
| P97 | NOTCH1 | NM_017617 | c.3973G>A | p.A1325T | nonsynonymous SNV | 0.49761 |
| P146 | NOTCH1 | NM_017617 | c.182A>G | p.N61S | nonsynonymous SNV | 0.01608 |
| P188 | NOTCH1 | NM_017617 | c.6091G>A | p.A2031T | nonsynonymous SNV | 0.15071 |
| P44 | NOTCH1 | NM_017617 | c.3224G>A | p.W1075X | stopgain | 0.01098 |
| P54 | NOTCH2 | NM_024408 | c.3820A>T | p.S1274C | nonsynonymous SNV | 0.23591 |
| P156 | NOTCH2 | NM_024408 | c.224A>G | p.N75S | nonsynonymous SNV | 0.01382 |
| P78 | NOTCH2 | NM_024408 | c.5065A>T | p.I1689F | nonsynonymous SNV | 0.02013 |
| P107 | NOTCH2 | NM_024408 | c.6622C>T | p.Q2208X | stopgain | 0.01239 |
| P208 | NOTCH2 | NM_024408 | c.4027C>T | p.Q1343X | stopgain | 0.06183 |
| P194 | NOTCH2 | NM_024408 | c.1017delT | p.I339fs | frameshift deletion | 0.30841 |
| P194 | NOTCH2 | NM_024408 | c.990_1014del | p.W330fs | frameshift deletion | 0.26424 |
| P36 | NOTCH2 | - | - | - | loss | 0.97 |
| P40 | NOTCH2 | - | - | - | loss | 0.75 |
| P56 | NOTCH2 | - | - | - | loss | 0.83 |
| P166 | NOTCH3 | NM_000435 | c.5126A>G | p.K1709R | nonsynonymous SNV | 0.05184 |
| P33 | NOTCH3 | NM_000435 | c.5066G>A | p.G1689D | nonsynonymous SNV | 0.4455 |
| P228 | NOTCH3 | NM_000435 | c.6173C>T | p.S2058L | nonsynonymous SNV | 0.19493 |
| P229 | NOTCH3 | NM_000435 | c.5243G>A | p.R1748H | nonsynonymous SNV | 0.36218 |
| P74 | NOTCH3 | NM_000435 | c.6102delC | p.P2034fs | frameshift deletion | 0.01077 |
| P230 | NOTCH4 | NM_004557 | c.3077T>C | p.L1026P | nonsynonymous SNV | 0.17101 |
| P78 | NOTCH4 | NM_004557 | c.1949T>A | p.L650H | nonsynonymous SNV | 0.02432 |
| P102 | NOTCH4 | NM_004557 | c.1019A>G | p.H340R | nonsynonymous SNV | 0.01189 |
| P144 | NOTCH4 | NM_004557 | c.4936C>T | p.R1646W | nonsynonymous SNV | 0.02842 |
| P197 | NOTCH4 | NM_004557 | c.4405C>T | p.Q1469X | stopgain | 0.47629 |
| P5 | NRAS | NM_002524 | c.181C>A | p.Q61K | nonsynonymous SNV | 0.02758 |
| P89 | NRG1 | NM_013957 | c.545G>T | p.C182F | nonsynonymous SNV | 0.08997 |
| P111 | NRG1 | NM_013957 | c.140C>A | p.S47X | stopgain | 0.01051 |
| P195 | NSD1 | NM_172349 | c.3471G>T | p.K1157N | nonsynonymous SNV | 0.18621 |
| P130 | NSD1 | NM_172349 | c.3361G>A | p.E1121K | nonsynonymous SNV | 0.0992 |
| P162 | NSD1 | NM_172349 | c.5794T>C | p.S1932P | nonsynonymous SNV | 0.21814 |
| P265 | NSD1 | NM_172349 | c.3471dupG | p.K1157fs | frameshift insertion | 0.03234 |
| P61 | NSD3 | NM_023034 | c.578G>A | p.R193K | nonsynonymous SNV | 0.01141 |
| P102 | NT5C2 | NM_001351172 | c.274G>T | p.D92Y | nonsynonymous SNV | 0.02432 |
| P231 | NTRK1 | NM_002529 | c.2075G>A | p.R692H | nonsynonymous SNV | 0.03362 |
| P194 | NTRK1 | NM_002529 | c.2368G>T | p.V790F | nonsynonymous SNV | 0.29079 |
| P26 | NTRK1 | NM_002529 | c.427C>G | p.L143V | nonsynonymous SNV | 0.01493 |
| P123 | NTRK2 | NM_006180 | c.1103G>T | p.G368V | nonsynonymous SNV | 0.03155 |
| P232 | NTRK2 | NM_006180 | c.1264G>A | p.V422I | nonsynonymous SNV | 0.01507 |
| P195 | NTRK2 | NM_006180 | c.2114T>A | p.L705Q | nonsynonymous SNV | 0.4 |
| P91 | NTRK2 | NM_006180 | c.1898T>C | p.F633S | nonsynonymous SNV | 0.21755 |
| P92 | NTRK2 | NM_006180 | c.186T>A | p.S62R | nonsynonymous SNV | 0.25745 |
| P233 | NTRK3 | NM_002530 | c.665G>C | p.R222P | nonsynonymous SNV | 0.09394 |
| P205 | NTRK3 | NM_002530 | c.1586-1G>T | - | - | 0.17337 |
| P20 | NUF2 | NM_031423 | c.37G>C | p.E13Q | nonsynonymous SNV | 0.22041 |
| P70 | NUTM1 | NM_175741 | c.2825A>C | p.K942T | nonsynonymous SNV | 0.01114 |
| P141 | NUTM1 | NM_175741 | c.3088C>G | p.L1030V | nonsynonymous SNV | 0.125 |
| P226 | OBSCN | NM_001271223 | c.1841C>G | p.A614G | nonsynonymous SNV | 0.39167 |
| P226 | OR4C3 | NM_001004702 | c.195T>G | p.C65W | nonsynonymous SNV | 0.04375 |
| P69 | PAK6 | NM_001276718 | c.1250C>T | p.S417F | nonsynonymous SNV | 0.12333 |
| P49 | PAK6 | NM_001276718 | c.191T>A | p.L64H | nonsynonymous SNV | 0.23703 |
| P127 | PAK6 | NM_001276718 | c.1336A>G | p.R446G | nonsynonymous SNV | 0.08816 |
| P70 | PALB2 | NM_024675 | c.3076C>G | p.L1026V | nonsynonymous SNV | 0.02135 |
| P145 | PALLD | NM_001166108 | c.2546G>A | p.G849E | nonsynonymous SNV | 0.0225 |
| P157 | PALLD | NM_001166108 | c.1747G>C | p.E583Q | nonsynonymous SNV | 0.01015 |
| P159 | PALLD | NM_001166108 | c.3364C>T | p.R1122X | stopgain | 0.0591 |
| P195 | PARP1 | NM_001618 | c.2558A>T | p.Q853L | nonsynonymous SNV | 0.44211 |
| P189 | PARP1 | NM_001618 | c.1012G>T | p.E338X | stopgain | 0.01923 |
| P173 | PAX5 | NM_016734 | c.187G>A | p.G63S | nonsynonymous SNV | 0.3113 |
| P234 | PBRM1 | NM_018313 | c.2715G>C | p.E905D | nonsynonymous SNV | 0.30474 |
| P235 | PBRM1 | NM_018313 | c.1949A>G | p.K650R | nonsynonymous SNV | 0.05502 |
| P236 | PBRM1 | NM_018313 | c.818C>A | p.A273E | nonsynonymous SNV | 0.26047 |
| P129 | PBRM1 | NM_018313 | c.2126T>A | p.I709N | nonsynonymous SNV | 0.56858 |
| P173 | PBRM1 | NM_018313 | c.2249T>C | p.L750P | nonsynonymous SNV | 0.38806 |
| P27 | PBRM1 | NM_018313 | c.755T>C | p.I252T | nonsynonymous SNV | 0.0288 |
| P237 | PBRM1 | NM_018313 | c.1565G>C | p.R522P | nonsynonymous SNV | 0.417 |
| P218 | PBRM1 | NM_018313 | c.1649C>T | p.P550L | nonsynonymous SNV | 0.24887 |
| P243 | PBRM1 | NM_018313 | c.1750G>T | p.E584X | stopgain | 0.18992 |
| P159 | PBRM1 | NM_018313 | c.3142delA | p.I1048fs | frameshift deletion | 0.35595 |
| P238 | PBRM1 | NM_018313 | c.1378A>T | p.K460X | stopgain | 0.08179 |
| P143 | PBRM1 | NM_018313 | c.2876_2880del | p.H959fs | frameshift deletion | 0.05664 |
| P109 | PBRM1 | NM_018313 | c.835dupA | p.I279fs | frameshift insertion | 0.15556 |
| P177 | PBRM1 | NM_018313 | c.1359_1362del | p.N453fs | frameshift deletion | 0.30438 |
| P249 | PBRM1 | NM_018313 | c.1583_1589del | p.N528fs | frameshift deletion | 0.06553 |
| P176 | PBRM1 | NM_018313 | c.835dupA | p.I279fs | frameshift insertion | 0.37673 |
| P93 | PBRM1 | NM_018313 | c.806_809del | p.V269fs | frameshift deletion | 0.05735 |
| P224 | PBRM1 | NM_018313 | c.1681delA | p.I561fs | frameshift deletion | 0.2549 |
| P105 | PBRM1 | NM_018313 | c.1308delA | p.K436fs | frameshift deletion | 0.21802 |
| P190 | PBRM1 | NM_018313 | c.2970_2971insTACACGAAAATTTCTAGAAAAA | p.E991fs | frameshift insertion | 0.20038 |
| P234 | PBRM1 | NM_018313 | c.2725A>T | p.K909X | stopgain | 0.30545 |
| P40 | PBRM1 | NM_018313 | c.1159delT | p.Y387fs | frameshift deletion | 0.3064 |
| P37 | PBRM1 | NM_018313 | c.2237_2244del | p.Y746fs | frameshift deletion | 0.325 |
| P161 | PBRM1 | NM_018313 | c.2161delA | p.I721fs | frameshift deletion | 0.37581 |
| P32 | PBRM1 | NM_018313 | c.2487delT | p.V829fs | frameshift deletion | 0.15529 |
| P266 | PBRM1 | NM_018313 | c.4457dupT | p.M1486fs | frameshift insertion | 0.10243 |
| P258 | PBRM1 | NM_018313 | c.3396delG | p.W1132fs | frameshift deletion | 0.1226 |
| P244 | PBRM1 | NM_018313 | c.3723_3724del | p.K1241fs | frameshift deletion | 0.58433 |
| P71 | PBRM1 | NM_018313 | c.1330delA | p.T444fs | frameshift deletion | 0.07033 |
| P61 | PBRM1 | NM_018313 | c.3123_3124insTGAATCACGGTATTCTGCCAAA | p.T1042_K1043delinsX | stopgain | 0.31354 |
| P88 | PBRM1 | NM_018313 | c.1658delA | p.K553fs | frameshift deletion | 0.36223 |
| P245 | PBRM1 | NM_018313 | c.548delA | p.K183fs | frameshift deletion | 0.26515 |
| P235 | PBRM1 | NM_018313 | c.1950_1953del | p.K650fs | frameshift deletion | 0.05425 |
| P260 | PBRM1 | NM_018313 | c.2260A>T | p.K754X | stopgain | 0.02924 |
| P178 | PBRM1 | NM_018313 | c.3102_3103del | p.C1034fs | frameshift deletion | 0.10265 |
| P78 | PBRM1 | NM_018313 | c.2327dupT | p.L776fs | frameshift insertion | 0.11583 |
| P191 | PBRM1 | NM_018313 | c.577_580del | p.I193fs | frameshift deletion | 0.4312 |
| P22 | PBRM1 | NM_018313 | c.844_845del | p.M282fs | frameshift deletion | 0.32258 |
| P102 | PBRM1 | NM_018313 | c.2737_2740del | p.E913fs | frameshift deletion | 0.18492 |
| P67 | PBRM1 | NM_018313 | c.4363G>T | p.G1455X | stopgain | 0.06388 |
| P138 | PBRM1 | NM_018313 | c.2488G>T | p.E830X | stopgain | 0.03957 |
| P220 | PBRM1 | NM_018313 | c.4576_4576del | p.A1526fs | frameshift deletion | 0.0579 |
| P130 | PBRM1 | NM_018313 | c.1542_1542del | p.S514fs | frameshift deletion | 0.13213 |
| P130 | PBRM1 | NM_018313 | c.974delA | p.N325fs | frameshift deletion | 0.03371 |
| P163 | PBRM1 | NM_018313 | c.1023_1042del | p.R341fs | frameshift deletion | 0.29443 |
| P247 | PBRM1 | NM_018313 | c.4232delC | p.P1411fs | frameshift deletion | 0.03366 |
| P189 | PBRM1 | NM_018313 | c.2645delA | p.N882fs | frameshift deletion | 0.10514 |
| P216 | PBRM1 | NM_018313 | c.3592dupA | p.T1198fs | frameshift insertion | 0.14891 |
| P172 | PBRM1 | NM_018313 | c.828delT | p.I276fs | frameshift deletion | 0.48023 |
| P151 | PBRM1 | NM_018313 | c.1858G>T | p.E620X | stopgain | 0.10055 |
| P175 | PBRM1 | NM_018313 | c.2469_2470del | p.D823fs | frameshift deletion | 0.30617 |
| P35 | PBRM1 | NM_018313 | c.3564dupA | p.E1189fs | frameshift insertion | 0.36239 |
| P34 | PBRM1 | NM_018313 | c.1785delG | p.R595fs | frameshift deletion | 0.24212 |
| P36 | PBRM1 | NM_018313 | c.3758C>A | p.S1253X | stopgain | 0.27609 |
| P145 | PBRM1 | NM_018313 | c.1058C>A | p.S353X | stopgain | 0.15317 |
| P145 | PBRM1 | NM_018313 | c.532delT | p.S178fs | frameshift deletion | 0.05411 |
| P194 | PBRM1 | NM_018313 | c.4055delA | p.Y1352fs | frameshift deletion | 0.45428 |
| P95 | PBRM1 | NM_018313 | c.3142delA | p.I1048fs | frameshift deletion | 0.30695 |
| P148 | PBRM1 | NM_018313 | c.3971delG | p.G1324fs | frameshift deletion | 0.52308 |
| P31 | PBRM1 | NM_018313 | c.2480delA | p.K827fs | frameshift deletion | 0.36415 |
| P144 | PBRM1 | NM_018313 | c.3961A>T | p.K1321X | stopgain | 0.66359 |
| P254 | PBRM1 | NM_018313 | c.2449_2452del | p.K817fs | frameshift deletion | 0.30049 |
| P165 | PBRM1 | NM_018313 | c.2719A>T | p.K907X | stopgain | 0.38692 |
| P155 | PBRM1 | NM_018313 | c.3919G>T | p.E1307X | stopgain | 0.32564 |
| P156 | PBRM1 | NM_018313 | c.3617-1G>A | - | - | 0.421 |
| P186 | PBRM1 | NM_018313 | c.645+1G>C | - | - | 0.17602 |
| P99 | PBRM1 | NM_018313 | c.1087+1G>A | - | - | 0.34035 |
| P185 | PBRM1 | NM_018313 | c.138+1G>C | - | - | 0.2249 |
| P168 | PBRM1 | NM_018313 | c.3312+1G>T | - | - | 0.31298 |
| P98 | PBRM1 | NM_018313 | c.2567+1G>T | - | - | 0.01382 |
| P210 | PCDH10 | NM_032961 | c.2854C>T | p.R952W | nonsynonymous SNV | 0.02174 |
| P16 | PCDH10 | NM_032961 | c.2629G>A | p.E877K | nonsynonymous SNV | 0.2748 |
| P133 | PCDH10 | NM_032961 | c.2149A>G | p.I717V | nonsynonymous SNV | 0.10425 |
| P141 | PCDH10 | NM_032961 | c.1576T>A | p.Y526N | nonsynonymous SNV | 0.17051 |
| P164 | PCDH10 | NM_032961 | c.673delC | p.P225fs | frameshift deletion | 0.01173 |
| P238 | PCDH17 | NM_001040429 | c.1375G>A | p.A459T | nonsynonymous SNV | 0.18534 |
| P93 | PCDH17 | NM_001040429 | c.1136G>C | p.R379P | nonsynonymous SNV | 0.11807 |
| P214 | PCDH17 | NM_001040429 | c.847A>T | p.S283C | nonsynonymous SNV | 0.20838 |
| P72 | PCDH17 | NM_001040429 | c.629C>G | p.T210R | nonsynonymous SNV | 0.04396 |
| P106 | PDCD1 | NM_005018 | c.691C>T | p.R231X | stopgain | 0.06552 |
| P182 | PDGFRA | NM_006206 | c.271T>C | p.S91P | nonsynonymous SNV | 0.01359 |
| P104 | PDGFRA | NM_006206 | c.1541T>A | p.L514Q | nonsynonymous SNV | 0.42197 |
| P165 | PDGFRA | NM_006206 | c.525A>T | p.R175S | nonsynonymous SNV | 0.02757 |
| P104 | PDGFRA | NM_006206 | c.1503C>A | p.C501X | stopgain | 0.42344 |
| P239 | PDGFRB | NM_002609 | c.2308A>T | p.N770Y | nonsynonymous SNV | 0.36815 |
| P34 | PDGFRB | - | - | - | gain | 4.36 |
| P36 | PDGFRB | - | - | - | gain | 4.01 |
| P49 | PDGFRB | - | - | - | gain | 4.34 |
| P120 | PDK1 | NM_002610 | c.664A>T | p.N222Y | nonsynonymous SNV | 0.21907 |
| P40 | PEG3 | NM_006210 | c.1070G>T | p.R357M | nonsynonymous SNV | 0.12394 |
| P217 | PEG3 | NM_006210 | c.4215A>C | p.E1405D | nonsynonymous SNV | 0.05461 |
| P153 | PEG3 | NM_006210 | c.2071C>T | p.R691W | nonsynonymous SNV | 0.19697 |
| P232 | PGR | NM_000926 | c.2477T>A | p.L826H | nonsynonymous SNV | 0.01499 |
| P47 | PGR | NM_000926 | c.1790G>T | p.G597V | nonsynonymous SNV | 0.02338 |
| P114 | PIK3C2G | NM_004570 | c.2099G>T | p.G700V | nonsynonymous SNV | 0.1113 |
| P120 | PIK3C3 | NM_002647 | c.1001T>C | p.L334S | nonsynonymous SNV | 0.30991 |
| P189 | PIK3C3 | NM_002647 | c.957_960del | p.F319fs | frameshift deletion | 0.07892 |
| P245 | PIK3C3 | NM_002647 | c.2188+1G>T | - | - | 0.16929 |
| P238 | PIK3CA | NM_006218 | c.3140A>G | p.H1047R | nonsynonymous SNV | 0.01633 |
| P76 | PIK3CA | NM_006218 | c.1624G>A | p.E542K | nonsynonymous SNV | 0.09475 |
| P113 | PIK3CA | NM_006218 | c.2852G>A | p.R951H | nonsynonymous SNV | 0.01353 |
| P49 | PIK3CA | NM_006218 | c.2707G>A | p.G903R | nonsynonymous SNV | 0.22985 |
| P96 | PIK3CA | NM_006218 | c.1624G>A | p.E542K | nonsynonymous SNV | 0.0102 |
| P215 | PIK3CA | NM_006218 | c.3073A>G | p.T1025A | nonsynonymous SNV | 0.0252 |
| P215 | PIK3CA | NM_006218 | c.3127A>G | p.M1043V | nonsynonymous SNV | 0.13038 |
| P78 | PIK3CA | NM_006218 | c.1624G>A | p.E542K | nonsynonymous SNV | 0.01302 |
| P146 | PIK3CA | NM_006218 | c.3104C>T | p.A1035V | nonsynonymous SNV | 0.01594 |
| P133 | PIK3CA | NM_006218 | c.1624G>C | p.E542Q | nonsynonymous SNV | 0.09274 |
| P44 | PIK3CA | NM_006218 | c.1633G>A | p.E545K | nonsynonymous SNV | 0.14113 |
| P15 | PIK3CA | NM_006218 | c.1357G>A | p.E453K | nonsynonymous SNV | 0.36188 |
| P184 | PIK3CB | NM_006219 | c.3151G>A | p.E1051K | nonsynonymous SNV | 0.1764 |
| P184 | PIK3CB | NM_006219 | c.3143C>T | p.A1048V | nonsynonymous SNV | 0.17532 |
| P140 | PIK3CD | NM_005026 | c.1387C>A | p.P463T | nonsynonymous SNV | 0.18983 |
| P98 | PIK3CG | NM_001282427 | c.1866G>T | p.L622F | nonsynonymous SNV | 0.01421 |
| P228 | PIK3R1 | NM_181523 | c.1072C>T | p.R358X | stopgain | 0.08074 |
| P175 | PIK3R1 | NM_181523 | c.1801G>T | p.E601X | stopgain | 0.31932 |
| P63 | PIK3R1 | - | - | - | loss | 0.305 |
| P111 | PIK3R2 | NM_005027 | c.1895C>T | p.A632V | nonsynonymous SNV | 0.01681 |
| P5 | PIM1 | NM_001243186 | c.137G>A | p.R46H | nonsynonymous SNV | 0.54698 |
| P142 | PIM1 | NM_002648 | c.131_134del | p.L44fs | frameshift deletion | 0.01781 |
| P142 | PIM1 | NM_002648 | c.136_143del | p.S46fs | frameshift deletion | 0.01666 |
| P222 | PKD2 | NM_000297 | c.1372C>T | p.Q458X | stopgain | 0.21886 |
| P149 | PLCG2 | NM_002661 | c.2626C>A | p.L876M | nonsynonymous SNV | 0.23252 |
| P152 | PLK2 | NM_006622 | c.1711G>A | p.V571M | nonsynonymous SNV | 0.29503 |
| P15 | PLK2 | NM_006622 | c.2032A>G | p.N678D | nonsynonymous SNV | 0.0595 |
| P161 | PLK2 | NM_006622 | c.910_911insCT | p.L304fs | frameshift insertion | 0.01656 |
| P44 | PLPPR4 | NM_001166252 | c.1048G>C | p.E350Q | nonsynonymous SNV | 0.16305 |
| P97 | PMS1 | NM_000534 | c.2612G>A | p.R871H | nonsynonymous SNV | 0.437 |
| P46 | PMS1 | NM_000534 | c.1748A>C | p.Q583P | nonsynonymous SNV | 0.10872 |
| P63 | PMS1 | - | - | - | loss | 0.455 |
| P55 | PMS2 | NM_000535 | c.2239A>G | p.R747G | nonsynonymous SNV | 0.16057 |
| P3 | PMS2 | NM_000535 | c.631C>T | p.R211X | stopgain | 0.04364 |
| P98 | POLD1 | NM_002691 | c.2000G>C | p.R667P | nonsynonymous SNV | 0.45512 |
| P133 | POLD1 | NM_002691 | c.1835T>C | p.M612T | nonsynonymous SNV | 0.12931 |
| P133 | POLD1 | NM_002691 | c.3179G>A | p.R1060H | nonsynonymous SNV | 0.51935 |
| P79 | POLE | NM_006231 | c.1841C>T | p.P614L | nonsynonymous SNV | 0.47107 |
| P200 | POLE | NM_006231 | c.1529T>G | p.F510C | nonsynonymous SNV | 0.21163 |
| P171 | POLE | NM_006231 | c.1393G>A | p.A465T | nonsynonymous SNV | 0.01706 |
| P258 | POLE | NM_006231 | c.1156G>T | p.E386X | stopgain | 0.10276 |
| P259 | POLE | NM_006231 | c.5690delA | p.K1897fs | frameshift deletion | 0.2179 |
| P188 | POM121L12 | NM_182595 | c.448C>T | p.P150S | nonsynonymous SNV | 0.03854 |
| P153 | PPARG | NM_015869 | c.298C>G | p.Q100E | nonsynonymous SNV | 0.27072 |
| P136 | PPM1D | NM_003620 | c.442G>T | p.A148S | nonsynonymous SNV | 0.05146 |
| P133 | PPM1D | NM_003620 | c.1655G>A | p.R552Q | nonsynonymous SNV | 0.50627 |
| P15 | PPM1D | NM_003620 | c.863T>G | p.F288C | nonsynonymous SNV | 0.03305 |
| P213 | PPP2R1A | NM_014225 | c.1376G>A | p.R459H | nonsynonymous SNV | 0.02016 |
| P159 | PPP6C | NM_001123355 | c.919dupT | p.S307fs | frameshift insertion | 0.36122 |
| P86 | PRDM1 | NM_001198 | c.1553C>T | p.T518M | nonsynonymous SNV | 0.15879 |
| P72 | PRDM1 | NM_001198 | c.839T>A | p.L280H | nonsynonymous SNV | 0.1705 |
| P149 | PRDM1 | NM_001198 | c.2102G>A | p.S701N | nonsynonymous SNV | 0.2241 |
| P49 | PREX2 | NM_024870 | c.3478A>T | p.S1160C | nonsynonymous SNV | 0.24471 |
| P53 | PREX2 | NM_024870 | c.4180C>T | p.R1394W | nonsynonymous SNV | 0.46154 |
| P204 | PREX2 | NM_024870 | c.476A>C | p.D159A | nonsynonymous SNV | 0.06691 |
| P103 | PREX2 | NM_024870 | c.2239C>T | p.R747W | nonsynonymous SNV | 0.20312 |
| P186 | PREX2 | NM_024870 | c.2667C>A | p.C889X | stopgain | 0.02622 |
| P128 | PRKAR1A | NM_212471 | c.589G>T | p.G197X | stopgain | 0.23358 |
| P213 | PRKCI | NM_002740 | c.1313C>T | p.A438V | nonsynonymous SNV | 0.25048 |
| P37 | PRKCI | NM_002740 | c.169_170del | p.M57fs | frameshift deletion | 0.23629 |
| P164 | PRKD1 | NM_001330069 | c.1906C>T | p.R636C | nonsynonymous SNV | 0.01474 |
| P181 | PRKDC | NM_001081640 | c.232-2A>T | - | - | 0.3173 |
| P26 | PRKN | NM_013988 | c.811C>T | p.R271C | nonsynonymous SNV | 0.0102 |
| P97 | PTCH1 | NM_000264 | c.3907C>T | p.R1303C | nonsynonymous SNV | 0.45036 |
| P10 | PTCH1 | NM_000264 | c.1124A>T | p.H375L | nonsynonymous SNV | 0.31973 |
| P85 | PTCH1 | NM_000264 | c.3078C>G | p.H1026Q | nonsynonymous SNV | 0.18623 |
| P145 | PTCH1 | NM_000264 | c.749G>A | p.G250D | nonsynonymous SNV | 0.18482 |
| P240 | PTEN | NM_000314 | c.509G>T | p.S170I | nonsynonymous SNV | 0.81113 |
| P78 | PTEN | NM_000314 | c.182A>C | p.H61P | nonsynonymous SNV | 0.04709 |
| P78 | PTEN | NM_000314 | c.389G>T | p.R130L | nonsynonymous SNV | 0.13949 |
| P67 | PTEN | NM_000314 | c.320A>T | p.D107V | nonsynonymous SNV | 0.04126 |
| P188 | PTEN | NM_000314 | c.389G>C | p.R130P | nonsynonymous SNV | 0.1013 |
| P140 | PTEN | NM_000314 | c.166delT | p.F56fs | frameshift deletion | 0.35784 |
| P140 | PTEN | NM_000314 | c.466delG | p.G156fs | frameshift deletion | 0.04463 |
| P100 | PTEN | NM_000314 | c.950_953del | p.V317fs | frameshift deletion | 0.19438 |
| P39 | PTEN | NM_000314 | c.802-1G>T | - | - | 0.07046 |
| P258 | PTEN | NM_000314 | c.634+2->A | - | - | 0.1414 |
| P106 | PTP4A1 | NM_003463 | c.242T>A | p.V81D | nonsynonymous SNV | 0.24279 |
| P241 | PTPRD | NM_130393 | c.53C>T | p.T18M | nonsynonymous SNV | 0.04742 |
| P78 | PTPRD | NM_002839 | c.2808G>T | p.Q936H | nonsynonymous SNV | 0.02174 |
| P91 | PTPRD | NM_130393 | c.1414A>G | p.T472A | nonsynonymous SNV | 0.07011 |
| P72 | PTPRD | NM_002839 | c.2302G>C | p.V768L | nonsynonymous SNV | 0.01863 |
| P153 | PTPRD | NM_002839 | c.2908C>G | p.Q970E | nonsynonymous SNV | 0.25 |
| P267 | PTPRD | NM_002839 | c.1822A>T | p.K608X | stopgain | 0.16794 |
| P36 | PTPRD | NM_130393 | c.529C>T | p.Q177X | stopgain | 0.01273 |
| P46 | PTPRD | NM_002839 | c.2564delG | p.G855fs | frameshift deletion | 0.0868 |
| P242 | PTPRS | NM_130855 | c.1000A>T | p.N334Y | nonsynonymous SNV | 0.28692 |
| P16 | PTPRS | NM_130855 | c.4462T>C | p.F1488L | nonsynonymous SNV | 0.01028 |
| P243 | PTPRT | NM_133170 | c.4094C>T | p.T1365M | nonsynonymous SNV | 0.01642 |
| P159 | PTPRT | NM_133170 | c.3110C>A | p.T1037K | nonsynonymous SNV | 0.25386 |
| P125 | PTPRT | NM_133170 | c.1222G>A | p.E408K | nonsynonymous SNV | 0.01946 |
| P117 | PTPRT | NM_133170 | c.3806A>T | p.Q1269L | nonsynonymous SNV | 0.1055 |
| P244 | PTPRT | NM_133170 | c.2039C>G | p.T680R | nonsynonymous SNV | 0.38118 |
| P245 | PTPRT | NM_133170 | c.3584A>T | p.K1195I | nonsynonymous SNV | 0.19416 |
| P72 | PTPRT | NM_133170 | c.287A>G | p.K96R | nonsynonymous SNV | 0.15043 |
| P49 | PXDNL | NM_144651 | c.2399C>T | p.T800M | nonsynonymous SNV | 0.23905 |
| P238 | RAD21 | NM_006265 | c.1321+1G>C | - | - | 0.06711 |
| P199 | RAD21 | NM_006265 | c.1322-1G>A | - | - | 0.40074 |
| P246 | RAD50 | NM_005732 | c.3709C>G | p.L1237V | nonsynonymous SNV | 0.23597 |
| P206 | RAD50 | NM_005732 | c.2157dupA | p.L719fs | frameshift insertion | 0.24231 |
| P63 | RAD50 | - | - | - | loss | 0.296 |
| P113 | RAD51 | NM_002875 | c.394G>A | p.G132R | nonsynonymous SNV | 0.02907 |
| P33 | RAF1 | - | - | - | gain | 6.14 |
| P96 | RANBP2 | NM_006267 | c.6856C>G | p.Q2286E | nonsynonymous SNV | 0.06117 |
| P124 | RARA | NM_000964 | c.800A>G | p.D267G | nonsynonymous SNV | 0.25493 |
| P93 | RARA | NM_001024809 | c.19G>T | p.V7L | nonsynonymous SNV | 0.10389 |
| P255 | RARA | NM_000964 | c.1313delC | p.A438fs | frameshift deletion | 0.01002 |
| P182 | RARA | NM_000964 | c.1313delC | p.A438fs | frameshift deletion | 0.01101 |
| P63 | RASA1 | - | - | - | loss | 0.151 |
| P5 | RASA1 | - | - | - | loss | 0.002 |
| P5 | RB1 | - | - | - | loss | 0.011 |
| P78 | RBM10 | NM_001204468 | c.647_648del | p.S216fs | frameshift deletion | 0.01271 |
| P197 | RBM10 | - | - | - | Fusion | 0.8824 |
| P204 | RECQL4 | NM_004260 | c.1250C>A | p.P417H | nonsynonymous SNV | 0.02451 |
| P60 | RECQL4 | - | - | - | loss | 0.93 |
| P155 | REG3A | NM_138937 | c.347A>T | p.N116I | nonsynonymous SNV | 0.22046 |
| P192 | REL | NM_001291746 | c.1063T>A | p.Y355N | nonsynonymous SNV | 0.13393 |
| P108 | RET | NM_020975 | c.1838C>A | p.P613H | nonsynonymous SNV | 0.21484 |
| P120 | RET | NM_020975 | c.2693A>T | p.D898V | nonsynonymous SNV | 0.32423 |
| P61 | REV3L | NM_002912 | c.3231G>C | p.R1077S | nonsynonymous SNV | 0.03078 |
| P113 | RFWD2 | NM_001286644 | c.637G>C | p.V213L | nonsynonymous SNV | 0.33245 |
| P204 | RFWD2 | NM_001001740 | c.440A>T | p.Y147F | nonsynonymous SNV | 0.04183 |
| P161 | RHBDF2 | NM_024599 | c.498_504del | p.S166fs | frameshift deletion | 0.21045 |
| P57 | RHBDF2 | NM_024599 | c.932delC | p.P311fs | frameshift deletion | 0.01092 |
| P246 | RHEB | NM_005614 | c.103T>A | p.Y35N | nonsynonymous SNV | 0.19496 |
| P15 | RHOA | NM_001313943 | c.139G>A | p.E47K | nonsynonymous SNV | 0.01807 |
| P38 | RICTOR | NM_001285440 | c.2087G>T | p.G696V | nonsynonymous SNV | 0.02329 |
| P98 | RICTOR | NM_001285440 | c.293C>A | p.S98Y | nonsynonymous SNV | 0.47602 |
| P61 | RICTOR | - | - | - | gain | 4.1 |
| P129 | RNF43 | NM_017763 | c.1325A>C | p.D442A | nonsynonymous SNV | 0.36017 |
| P200 | RNF43 | NM_017763 | c.2328G>C | p.E776D | nonsynonymous SNV | 0.19745 |
| P15 | RNF43 | NM_017763 | c.181C>A | p.L61M | nonsynonymous SNV | 0.03807 |
| P150 | ROS1 | NM_002944 | c.4681G>A | p.V1561M | nonsynonymous SNV | 0.11876 |
| P232 | ROS1 | NM_002944 | c.6302G>T | p.G2101V | nonsynonymous SNV | 0.11409 |
| P182 | ROS1 | NM_002944 | c.3207G>C | p.W1069C | nonsynonymous SNV | 0.02148 |
| P207 | ROS1 | NM_002944 | c.6376C>T | p.R2126W | nonsynonymous SNV | 0.13327 |
| P117 | ROS1 | NM_002944 | c.403T>A | p.Y135N | nonsynonymous SNV | 0.08377 |
| P78 | ROS1 | NM_002944 | c.977C>G | p.T326R | nonsynonymous SNV | 0.01466 |
| P89 | ROS1 | NM_002944 | c.1540G>T | p.G514C | nonsynonymous SNV | 0.45836 |
| P30 | ROS1 | NM_002944 | c.5743G>A | p.G1915R | nonsynonymous SNV | 0.30805 |
| P168 | ROS1 | NM_002944 | c.5952G>C | p.K1984N | nonsynonymous SNV | 0.22181 |
| P47 | ROS1 | NM_002944 | c.5236T>G | p.F1746V | nonsynonymous SNV | 0.02022 |
| P124 | ROS1 | NM_002944 | c.4525G>T | p.E1509X | stopgain | 0.30947 |
| P147 | ROS1 | NM_002944 | c.5142T>A | p.C1714X | stopgain | 0.0347 |
| P71 | RP1L1 | NM_178857 | c.3359C>T | p.T1120I | nonsynonymous SNV | 0.02435 |
| P208 | RP1L1 | NM_178857 | c.4501G>A | p.E1501K | nonsynonymous SNV | 0.06109 |
| P152 | RP1L1 | NM_178857 | c.1591G>A | p.A531T | nonsynonymous SNV | 0.01604 |
| P179 | RPL5 | NM_000969 | c.161G>C | p.R54P | nonsynonymous SNV | 0.3388 |
| P34 | RPS19 | NM_001321485 | c.172G>C | p.G58R | nonsynonymous SNV | 0.16248 |
| P36 | RPS6KB2 | NM_003952 | c.241G>A | p.V81M | nonsynonymous SNV | 0.01032 |
| P63 | RPS6KB2 | - | - | - | gain | 4.434 |
| P19 | RPTOR | NM_001163034 | c.1490A>G | p.K497R | nonsynonymous SNV | 0.04975 |
| P12 | RRAS2 | NM_001177314 | c.245G>A | p.R82H | nonsynonymous SNV | 0.15262 |
| P93 | RTEL1 | NM_016434 | c.3175G>A | p.A1059T | nonsynonymous SNV | 0.01072 |
| P90 | RTEL1 | NM_016434 | c.668T>G | p.F223C | nonsynonymous SNV | 0.0109 |
| P62 | RUNX1 | - | - | - | gain | 4.853 |
| P66 | RUNX1 | - | - | - | gain | 7.928 |
| P207 | RUNX1T1 | NM_175635 | c.1070G>A | p.R357Q | nonsynonymous SNV | 0.10156 |
| P117 | RUNX1T1 | NM_175635 | c.1300G>T | p.A434S | nonsynonymous SNV | 0.09569 |
| P34 | RUNX1T1 | NM_175635 | c.1246G>A | p.V416I | nonsynonymous SNV | 0.16076 |
| P135 | RUNX1T1 | NM_175635 | c.1441T>A | p.C481S | nonsynonymous SNV | 0.33003 |
| P9 | RUNX3 | NM_001320672 | c.1045G>A | p.G349R | nonsynonymous SNV | 0.2656 |
| P73 | RUNX3 | NM_001320672 | c.929delG | p.G310fs | frameshift deletion | 0.01078 |
| P115 | RYBP | NM_012234 | c.194C>A | p.P65H | nonsynonymous SNV | 0.1337 |
| P153 | RYBP | NM_012234 | c.34G>A | p.E12K | nonsynonymous SNV | 0.03892 |
| P247 | SDC4 | NM_002999 | c.381C>A | p.N127K | nonsynonymous SNV | 0.04744 |
| P69 | SESN2 | NM_031459 | c.1191C>G | p.I397M | nonsynonymous SNV | 0.03189 |
| P156 | SESN2 | NM_031459 | c.556G>A | p.E186K | nonsynonymous SNV | 0.28209 |
| P204 | SETBP1 | NM_015559 | c.3805G>T | p.D1269Y | nonsynonymous SNV | 0.02692 |
| P15 | SETBP1 | NM_015559 | c.2915G>A | p.R972Q | nonsynonymous SNV | 0.05599 |
| P111 | SETD2 | NM_014159 | c.5068G>A | p.G1690R | nonsynonymous SNV | 0.01085 |
| P20 | SETD2 | NM_014159 | c.6377A>T | p.E2126V | nonsynonymous SNV | 0.01124 |
| P232 | SETD2 | NM_014159 | c.4862G>T | p.G1621V | nonsynonymous SNV | 0.01907 |
| P235 | SETD2 | NM_014159 | c.4678A>G | p.K1560E | nonsynonymous SNV | 0.03894 |
| P89 | SETD2 | NM_014159 | c.6746A>G | p.H2249R | nonsynonymous SNV | 0.03051 |
| P247 | SETD2 | NM_014159 | c.4848T>G | p.D1616E | nonsynonymous SNV | 0.03754 |
| P106 | SETD2 | NM_014159 | c.4622A>G | p.N1541S | nonsynonymous SNV | 0.12065 |
| P46 | SETD2 | NM_014159 | c.715A>G | p.K239E | nonsynonymous SNV | 0.07494 |
| P115 | SETD2 | NM_014159 | c.4740T>A | p.C1580X | stopgain | 0.06949 |
| P115 | SETD2 | NM_014159 | c.545delC | p.T182fs | frameshift deletion | 0.07009 |
| P94 | SETD2 | NM_014159 | c.4962delA | p.K1654fs | frameshift deletion | 0.02639 |
| P158 | SETD2 | NM_014159 | c.608C>A | p.S203X | stopgain | 0.26611 |
| P176 | SETD2 | NM_014159 | c.5656G>T | p.E1886X | stopgain | 0.40196 |
| P39 | SETD2 | NM_014159 | c.3415A>T | p.K1139X | stopgain | 0.0481 |
| P37 | SETD2 | NM_014159 | c.2421delT | p.S807fs | frameshift deletion | 0.38235 |
| P12 | SETD2 | NM_014159 | c.4275_4276del | p.K1425fs | frameshift deletion | 0.434 |
| P16 | SETD2 | NM_014159 | c.3787delT | p.S1263fs | frameshift deletion | 0.01353 |
| P230 | SETD2 | NM_014159 | c.2706_2707del | p.E902fs | frameshift deletion | 0.2007 |
| P99 | SETD2 | NM_014159 | c.2145delG | p.M715fs | frameshift deletion | 0.09414 |
| P204 | SETD2 | NM_014159 | c.4453A>T | p.R1485X | stopgain | 0.03354 |
| P245 | SETD2 | NM_014159 | c.2288dupT | p.M763fs | frameshift insertion | 0.26269 |
| P100 | SETD2 | NM_014159 | c.4271_4272del | p.R1424fs | frameshift deletion | 0.21442 |
| P250 | SETD2 | NM_014159 | c.6771dupG | p.P2258fs | frameshift insertion | 0.04717 |
| P250 | SETD2 | NM_014159 | c.1981delG | p.D661fs | frameshift deletion | 0.18237 |
| P129 | SETD2 | NM_014159 | c.1362_1363del | p.R454fs | frameshift deletion | 0.57687 |
| P248 | SETD2 | NM_014159 | c.6325C>T | p.R2109X | stopgain | 0.04194 |
| P178 | SETD2 | NM_014159 | c.1547delG | p.R516fs | frameshift deletion | 0.0607 |
| P22 | SETD2 | NM_014159 | c.5638A>T | p.R1880X | stopgain | 0.3652 |
| P67 | SETD2 | NM_014159 | c.5963_5964del | p.S1988fs | frameshift deletion | 0.0566 |
| P189 | SETD2 | NM_014159 | c.6861_6862del | p.S2287fs | frameshift deletion | 0.01949 |
| P175 | SETD2 | NM_014159 | c.7000C>T | p.Q2334X | stopgain | 0.27813 |
| P35 | SETD2 | NM_014159 | c.4992dupT | p.D1665_Y1666delinsX | stopgain | 0.3436 |
| P120 | SETD2 | NM_014159 | c.1670delC | p.S557fs | frameshift deletion | 0.4511 |
| P227 | SETD2 | NM_014159 | c.2076delT | p.D692fs | frameshift deletion | 0.19778 |
| P70 | SETD2 | NM_014159 | c.3592C>T | p.Q1198X | stopgain | 0.30733 |
| P144 | SETD2 | NM_014159 | c.3779G>A | p.W1260X | stopgain | 0.67925 |
| P135 | SETD2 | NM_014159 | c.7159A>T | p.K2387X | stopgain | 0.38449 |
| P141 | SETD2 | NM_014159 | c.3379delC | p.Q1127fs | frameshift deletion | 0.10227 |
| P249 | SETD2 | - | - | - | Fusion | 0.0864 |
| P40 | SETD2 | - | - | - | Fusion | 0.2869 |
| P248 | SF3B1 | NM_012433 | c.1729G>A | p.V577I | nonsynonymous SNV | 0.05357 |
| P89 | SF3B1 | NM_012433 | c.2221A>C | p.K741Q | nonsynonymous SNV | 0.02484 |
| P103 | SF3B1 | NM_012433 | c.3725G>T | p.G1242V | nonsynonymous SNV | 0.02924 |
| P5 | SH2B3 | NM_005475 | c.1606G>A | p.A536T | nonsynonymous SNV | 0.63587 |
| P6 | SH2B3 | NM_005475 | c.922C>T | p.R308X | stopgain | 0.30552 |
| P172 | SHOC2 | NM_001324336 | c.1677G>T | p.Q559H | nonsynonymous SNV | 0.31346 |
| P70 | SHQ1 | NM_018130 | c.1418C>G | p.S473X | stopgain | 0.01357 |
| P226 | SLC6A19 | NM_001003841 | c.1174-2A>C | - | - | 0.4625 |
| P226 | SLC7A8 | NM_012244 | c.550A>C | p.T184P | nonsynonymous SNV | 0.06114 |
| P167 | SLC8A1 | NM_001112802 | c.1640C>T | p.T547I | nonsynonymous SNV | 0.04286 |
| P114 | SLIT2 | NM_004787 | c.1705A>T | p.K569X | stopgain | 0.125 |
| P43 | SLITRK1 | NM_001281503 | c.407A>T | p.Q136L | nonsynonymous SNV | 0.14778 |
| P69 | SLITRK2 | NM_001144005 | c.1610T>A | p.I537N | nonsynonymous SNV | 0.02208 |
| P171 | SLITRK2 | NM_001144005 | c.2263C>A | p.L755M | nonsynonymous SNV | 0.0157 |
| P213 | SLITRK2 | NM_001144005 | c.175dupC | p.Q58fs | frameshift insertion | 0.01038 |
| P134 | SLITRK3 | NM_001318811 | c.140C>A | p.P47Q | nonsynonymous SNV | 0.01013 |
| P22 | SLX4 | NM_032444 | c.3916_3917del | p.Q1306fs | frameshift deletion | 0.24768 |
| P185 | SLX4 | NM_032444 | c.5215delG | p.E1739fs | frameshift deletion | 0.21492 |
| P168 | SLX4 | NM_032444 | c.5149C>T | p.Q1717X | stopgain | 0.06593 |
| P100 | SMAD2 | NM_001003652 | c.1172C>G | p.A391G | nonsynonymous SNV | 0.10789 |
| P106 | SMAD2 | NM_001003652 | c.21C>A | p.F7L | nonsynonymous SNV | 0.18583 |
| P257 | SMAD4 | NM_005359 | c.739delG | p.G247fs | frameshift deletion | 0.02585 |
| P124 | SMARCA4 | NM_003072 | c.3482T>C | p.L1161P | nonsynonymous SNV | 0.50551 |
| P241 | SMARCA4 | NM_003072 | c.1402C>T | p.R468C | nonsynonymous SNV | 0.0823 |
| P205 | SMARCA4 | NM_003072 | c.2799C>A | p.F933L | nonsynonymous SNV | 0.30268 |
| P140 | SMARCA4 | NM_003072 | c.2641G>A | p.D881N | nonsynonymous SNV | 0.02458 |
| P210 | SMARCA4 | NM_003072 | c.2956G>A | p.A986T | nonsynonymous SNV | 0.03107 |
| P13 | SMARCA4 | NM_003072 | c.4168A>T | p.K1390X | stopgain | 0.19672 |
| P27 | SMARCA4 | NM_003072 | c.2237C>G | p.S746X | stopgain | 0.25248 |
| P44 | SMARCA4 | NM_003072 | c.2460C>A | p.Y820X | stopgain | 0.02316 |
| P208 | SMARCD1 | NM_003076 | c.508G>C | p.E170Q | nonsynonymous SNV | 0.06867 |
| P89 | SMO | NM_005631 | c.1769G>C | p.S590T | nonsynonymous SNV | 0.4484 |
| P79 | SMO | NM_005631 | c.2102T>A | p.I701N | nonsynonymous SNV | 0.01028 |
| P79 | SMO | NM_005631 | c.2114C>T | p.P705L | nonsynonymous SNV | 0.47483 |
| P98 | SMO | NM_005631 | c.608C>T | p.P203L | nonsynonymous SNV | 0.01349 |
| P209 | SMO | NM_005631 | c.607C>A | p.P203T | nonsynonymous SNV | 0.0125 |
| P106 | SMO | NM_005631 | c.83delG | p.R28fs | frameshift deletion | 0.01376 |
| P6 | SNCAIP | NM_001308109 | c.818A>T | p.E273V | nonsynonymous SNV | 0.03942 |
| P83 | SOS1 | NM_005633 | c.512T>G | p.V171G | nonsynonymous SNV | 0.01451 |
| P173 | SOS1 | NM_005633 | c.3163C>A | p.P1055T | nonsynonymous SNV | 0.31532 |
| P54 | SOS1 | NM_005633 | c.1485dupT | p.M496fs | frameshift insertion | 0.2629 |
| P44 | SOX17 | NM_022454 | c.514G>A | p.G172S | nonsynonymous SNV | 0.1531 |
| P224 | SOX9 | NM_000346 | c.724A>C | p.K242Q | nonsynonymous SNV | 0.02249 |
| P38 | SOX9 | NM_000346 | c.1249C>A | p.Q417K | nonsynonymous SNV | 0.01524 |
| P213 | SOX9 | NM_000346 | c.841G>A | p.D281N | nonsynonymous SNV | 0.06141 |
| P70 | SOX9 | NM_000346 | c.715A>C | p.T239P | nonsynonymous SNV | 0.02235 |
| P167 | SPEN | NM_015001 | c.4789G>C | p.E1597Q | nonsynonymous SNV | 0.04911 |
| P90 | SPEN | NM_015001 | c.1514G>A | p.R505H | nonsynonymous SNV | 0.01037 |
| P70 | SPEN | NM_015001 | c.5749C>T | p.R1917C | nonsynonymous SNV | 0.01013 |
| P132 | SPEN | NM_015001 | c.2348delA | p.E783fs | frameshift deletion | 0.06897 |
| P104 | SPEN | NM_015001 | c.6159delC | p.N2053fs | frameshift deletion | 0.01003 |
| P213 | SPEN | NM_015001 | c.1319_1322del | p.L440fs | frameshift deletion | 0.01651 |
| P167 | SPHKAP | NM_001142644 | c.3549G>T | p.K1183N | nonsynonymous SNV | 0.06383 |
| P39 | SPOP | NM_001007228 | c.407T>C | p.F136S | nonsynonymous SNV | 0.04363 |
| P134 | SPOP | NM_001007228 | c.610T>A | p.L204M | nonsynonymous SNV | 0.1544 |
| P138 | SPOP | NM_001007228 | c.299C>A | p.A100E | nonsynonymous SNV | 0.02311 |
| P90 | SPOP | NM_001007228 | c.1013G>A | p.W338X | stopgain | 0.26772 |
| P31 | SPRED1 | NM_152594 | c.1106T>C | p.M369T | nonsynonymous SNV | 0.11821 |
| P213 | SPRED1 | NM_152594 | c.190C>T | p.R64X | stopgain | 0.28873 |
| P42 | SPTA1 | NM_003126 | c.5410C>T | p.L1804F | nonsynonymous SNV | 0.01002 |
| P116 | SPTA1 | NM_003126 | c.5070delT | p.N1690fs | frameshift deletion | 0.30787 |
| P79 | SRC | NM_005417 | c.1363C>G | p.L455V | nonsynonymous SNV | 0.49799 |
| P38 | SRSF2 | - | - | - | gain | 4.21 |
| P55 | STAG2 | NM_001042749 | c.130A>G | p.K44E | nonsynonymous SNV | 0.01916 |
| P59 | STAG2 | NM_001042749 | c.1714C>T | p.P572S | nonsynonymous SNV | 0.12842 |
| P103 | STAG2 | NM_001042749 | c.504G>T | p.W168C | nonsynonymous SNV | 0.03053 |
| P134 | STAG2 | NM_001042749 | c.1804_1817del | p.T602fs | frameshift deletion | 0.19192 |
| P100 | STAG2 | NM_001042749 | c.2982dupT | p.P994fs | frameshift insertion | 0.14983 |
| P257 | STAG2 | NM_001042749 | c.2560_2567del | p.K854fs | frameshift deletion | 0.63068 |
| P31 | STAG2 | NM_001042749 | c.2056C>T | p.Q686X | stopgain | 0.0592 |
| P144 | STAG2 | NM_001042749 | c.2097-2A>T | - | - | 0.82888 |
| P44 | STAT3 | NM_139276 | c.250C>T | p.R84X | stopgain | 0.14444 |
| P111 | STAT3 | NM_139276 | c.2145-2A>T | - | - | 0.20017 |
| P128 | STAT4 | NM_001243835 | c.2074G>T | p.G692C | nonsynonymous SNV | 0.21495 |
| P188 | STAT4 | NM_001243835 | c.290delA | p.N97fs | frameshift deletion | 0.09234 |
| P25 | STAT5A | NM_003152 | c.1096delC | p.P366fs | frameshift deletion | 0.01047 |
| P131 | STAT5A | NM_003152 | c.1096delC | p.P366fs | frameshift deletion | 0.0121 |
| P107 | STAT5A | NM_003152 | c.1096delC | p.P366fs | frameshift deletion | 0.01072 |
| P106 | STAT5A | NM_003152 | c.1096delC | p.P366fs | frameshift deletion | 0.01114 |
| P216 | STAT5A | NM_003152 | c.1096delC | p.P366fs | frameshift deletion | 0.01076 |
| P151 | STAT5A | NM_003152 | c.1096delC | p.P366fs | frameshift deletion | 0.0107 |
| P120 | STAT5A | NM_003152 | c.1096delC | p.P366fs | frameshift deletion | 0.0107 |
| P152 | STAT5A | NM_003152 | c.1096delC | p.P366fs | frameshift deletion | 0.01194 |
| P31 | STAT5A | NM_003152 | c.1096delC | p.P366fs | frameshift deletion | 0.01813 |
| P209 | STAT5A | NM_003152 | c.1096delC | p.P366fs | frameshift deletion | 0.01155 |
| P10 | STK19 | NM_004197 | c.689C>T | p.P230L | nonsynonymous SNV | 0.34454 |
| P50 | STK40 | NM_001282546 | c.706A>C | p.K236Q | nonsynonymous SNV | 0.10723 |
| P128 | STK40 | NM_001282546 | c.350T>G | p.L117R | nonsynonymous SNV | 0.1221 |
| P226 | STMND1 | NM_001190766 | c.376A>C | p.K126Q | nonsynonymous SNV | 0.13295 |
| P7 | SUZ12 | NM_015355 | c.1183A>T | p.K395X | stopgain | 0.11823 |
| P81 | SYK | NM_003177 | c.601G>A | p.G201S | nonsynonymous SNV | 0.02594 |
| P24 | SYK | NM_003177 | c.12C>A | p.S4R | nonsynonymous SNV | 0.15515 |
| P133 | SYK | NM_003177 | c.389A>G | p.E130G | nonsynonymous SNV | 0.58305 |
| P144 | SYK | NM_003177 | c.182_183del | p.A61fs | frameshift deletion | 0.39448 |
| P166 | TAF1 | NM_001286074 | c.2159A>T | p.K720M | nonsynonymous SNV | 0.04072 |
| P229 | TAF1 | NM_001286074 | c.766C>G | p.P256A | nonsynonymous SNV | 0.01502 |
| P232 | TBL1XR1 | NM_001321193 | c.699G>T | p.W233C | nonsynonymous SNV | 0.01565 |
| P232 | TBX3 | NM_005996 | c.1463C>T | p.A488V | nonsynonymous SNV | 0.02275 |
| P9 | TBX3 | NM_005996 | c.518C>T | p.A173V | nonsynonymous SNV | 0.13077 |
| P5 | TBX3 | NM_005996 | c.1625C>T | p.A542V | nonsynonymous SNV | 0.35138 |
| P198 | TBX3 | NM_005996 | c.1170delC | p.P390fs | frameshift deletion | 0.11789 |
| P144 | TCF3 | NM_003200 | c.653-2A>T | - | - | 0.39957 |
| P142 | TEK | NM_001290077 | c.2409T>A | p.D803E | nonsynonymous SNV | 0.05833 |
| P197 | TEK | NM_001290077 | c.3139G>A | p.V1047M | nonsynonymous SNV | 0.51385 |
| P59 | TEK | NM_001290077 | c.3206C>G | p.T1069S | nonsynonymous SNV | 0.01825 |
| P24 | TEK | NM_001290077 | c.3178G>A | p.V1060M | nonsynonymous SNV | 0.17345 |
| P91 | TEK | NM_001290077 | c.633T>A | p.C211X | stopgain | 0.21615 |
| P242 | TERT | NM_198253 | c.2599G>T | p.V867L | nonsynonymous SNV | 0.01208 |
| P38 | TERT | NM_198253 | c.336delC | p.P112fs | frameshift deletion | 0.01155 |
| P137 | TERT | NM_198253 | c.799delG | p.D267fs | frameshift deletion | 0.13631 |
| P37 | TERT | - | - | - | gain | 4.47 |
| P174 | TET1 | NM_030625 | c.5182A>T | p.K1728X | stopgain | 0.01264 |
| P54 | TET2 | NM_001127208 | c.5611A>T | p.I1871F | nonsynonymous SNV | 0.01955 |
| P135 | TET2 | NM_001127208 | c.5698G>A | p.V1900I | nonsynonymous SNV | 0.16519 |
| P54 | TET2 | NM_001127208 | c.5610_5611insG | p.S1870fs | frameshift insertion | 0.01955 |
| P52 | TET2 | NM_001127208 | c.1189_1190insTCAACACCACCACCACCAT | p.T397fs | frameshift insertion | 0.08161 |
| P167 | TET2 | NM_001127208 | c.1190dupC | p.T397fs | frameshift insertion | 0.0597 |
| P135 | TET2 | NM_001127208 | c.4015delA | p.K1339fs | frameshift deletion | 0.33267 |
| P143 | TET2 | NM_001127208 | c.4044+1G>T | - | - | 0.01355 |
| P226 | TFE3 | - | - | - | Fusion | 0.7586 |
| P79 | TFE3 | - | - | - | Fusion | 0.4362 |
| P94 | TFE3 | - | - | - | Fusion | 0.4762 |
| P55 | TFE3 | - | - | - | Fusion | 0.246 |
| P3 | TFEC | - | - | - | Fusion | 0.6316 |
| P25 | TGFBR1 | NM_004612 | c.782G>A | p.G261E | nonsynonymous SNV | 0.2 |
| P61 | TGFBR1 | NM_004612 | c.876_879del | p.L292fs | frameshift deletion | 0.27326 |
| P209 | TGFBR1 | NM_004612 | c.1291delC | p.L431fs | frameshift deletion | 0.01272 |
| P40 | TGFBR2 | NM_001024847 | c.365T>C | p.L122P | nonsynonymous SNV | 0.15217 |
| P92 | TLR4 | NM_003266 | c.1096C>A | p.L366M | nonsynonymous SNV | 0.2711 |
| P189 | TMEM127 | NM_001193304 | c.511G>T | p.V171F | nonsynonymous SNV | 0.07137 |
| P208 | TMPRSS2 | NM_005656 | c.439C>T | p.R147W | nonsynonymous SNV | 0.04575 |
| P24 | TMPRSS2 | NM_001135099 | c.91G>C | p.D31H | nonsynonymous SNV | 0.11291 |
| P78 | TMPRSS2 | NM_005656 | c.133T>G | p.Y45D | nonsynonymous SNV | 0.01267 |
| P211 | TMPRSS2 | NM_005656 | c.820C>G | p.H274D | nonsynonymous SNV | 0.20646 |
| P89 | TNFAIP3 | NM_006290 | c.673G>A | p.A225T | nonsynonymous SNV | 0.01754 |
| P120 | TNFAIP3 | NM_006290 | c.449A>T | p.Q150L | nonsynonymous SNV | 0.26479 |
| P111 | TNFRSF14 | NM_003820 | c.552-2A>T | - | - | 0.21121 |
| P63 | TOP1 | - | - | - | loss | 0.491 |
| P38 | TOP2A | NM_001067 | c.2893A>T | p.T965S | nonsynonymous SNV | 0.29015 |
| P167 | TOP2A | NM_001067 | c.4391G>A | p.R1464H | nonsynonymous SNV | 0.03509 |
| P192 | TP53 | NM_000546 | c.1175C>T | p.S392L | nonsynonymous SNV | 0.0126 |
| P87 | TP53 | NM_000546 | c.715A>T | p.N239Y | nonsynonymous SNV | 0.01871 |
| P87 | TP53 | NM_000546 | c.713G>T | p.C238F | nonsynonymous SNV | 0.01856 |
| P156 | TP53 | NM_000546 | c.733G>A | p.G245S | nonsynonymous SNV | 0.01054 |
| P161 | TP53 | NM_000546 | c.843C>A | p.D281E | nonsynonymous SNV | 0.07872 |
| P215 | TP53 | NM_000546 | c.742C>T | p.R248W | nonsynonymous SNV | 0.07955 |
| P129 | TP53 | NM_000546 | c.743G>A | p.R248Q | nonsynonymous SNV | 0.55431 |
| P59 | TP53 | NM_000546 | c.817C>T | p.R273C | nonsynonymous SNV | 0.14711 |
| P5 | TP53 | NM_000546 | c.517G>A | p.V173M | nonsynonymous SNV | 0.58198 |
| P106 | TP53 | NM_000546 | c.535C>T | p.H179Y | nonsynonymous SNV | 0.01881 |
| P216 | TP53 | NM_000546 | c.818G>A | p.R273H | nonsynonymous SNV | 0.12153 |
| P120 | TP53 | NM_000546 | c.743G>A | p.R248Q | nonsynonymous SNV | 0.01748 |
| P153 | TP53 | NM_000546 | c.578A>G | p.H193R | nonsynonymous SNV | 0.41609 |
| P153 | TP53 | NM_000546 | c.524G>A | p.R175H | nonsynonymous SNV | 0.26974 |
| P229 | TP53 | NM_000546 | c.818G>T | p.R273L | nonsynonymous SNV | 0.64097 |
| P201 | TP53 | NM_000546 | c.951_954del | p.Q317fs | frameshift deletion | 0.02429 |
| P241 | TP53 | NM_000546 | c.626_627del | p.R209fs | frameshift deletion | 0.72288 |
| P33 | TP53 | NM_000546 | c.372C>A | p.C124X | stopgain | 0.9847 |
| P96 | TP53 | NM_000546 | c.579dupT | p.L194fs | frameshift insertion | 0.20033 |
| P195 | TP53 | NM_000546 | c.370dupT | p.C124fs | frameshift insertion | 0.81099 |
| P184 | TP53 | NM_000546 | c.1024C>T | p.R342X | stopgain | 0.31852 |
| P106 | TP53 | NM_000546 | c.920-1G>C | - | - | 0.05327 |
| P137 | TP53 | NM_000546 | c.375+2T>C | - | - | 0.79094 |
| P44 | TP53 | NM_000546 | c.673-2A>T | - | - | 0.41279 |
| P39 | TP53 | - | - | - | loss | 0 |
| P48 | TP53 | - | - | - | loss | 0.91 |
| P55 | TP53 | - | - | - | loss | 0.83 |
| P76 | TP53BP1 | NM_001141980 | c.4981A>T | p.S1661C | nonsynonymous SNV | 0.20203 |
| P156 | TP53BP1 | NM_001141980 | c.1931C>G | p.S644C | nonsynonymous SNV | 0.29766 |
| P233 | TP53BP1 | NM_001141980 | c.1963delA | p.I655fs | frameshift deletion | 0.05584 |
| P211 | TP53BP1 | NM_001141980 | c.1094_1100del | p.S365fs | frameshift deletion | 0.19478 |
| P86 | TP63 | NM_001329964 | c.405C>A | p.D135E | nonsynonymous SNV | 0.07923 |
| P114 | TP63 | NM_001329964 | c.782A>G | p.H261R | nonsynonymous SNV | 0.1097 |
| P15 | TP63 | NM_001329964 | c.1775G>A | p.R592Q | nonsynonymous SNV | 0.04312 |
| P105 | TRAF2 | NM_021138 | c.101G>T | p.C34F | nonsynonymous SNV | 0.15851 |
| P93 | TRIM58 | NM_015431 | c.902A>C | p.H301P | nonsynonymous SNV | 0.01618 |
| P187 | TSC1 | NM_000368 | c.587C>T | p.P196L | nonsynonymous SNV | 0.08122 |
| P92 | TSC1 | NM_000368 | c.1831G>A | p.A611T | nonsynonymous SNV | 0.24846 |
| P102 | TSC1 | NM_000368 | c.687delT | p.I229fs | frameshift deletion | 0.1735 |
| P254 | TSC1 | NM_000368 | c.2820delG | p.Q940fs | frameshift deletion | 0.23052 |
| P61 | TSC2 | NM_000548 | c.670G>A | p.A224T | nonsynonymous SNV | 0.02124 |
| P79 | TSC2 | NM_000548 | c.4715C>T | p.T1572M | nonsynonymous SNV | 0.48431 |
| P15 | TSC2 | NM_000548 | c.3212C>T | p.T1071I | nonsynonymous SNV | 0.15305 |
| P18 | TSC2 | NM_000548 | c.3367delG | p.G1123fs | frameshift deletion | 0.11438 |
| P261 | TSC2 | NM_000548 | c.4753dupA | p.L1584fs | frameshift insertion | 0.02376 |
| P268 | TSC2 | NM_000548 | c.2033dupC | p.A678fs | frameshift insertion | 0.2163 |
| P10 | TSC2 | NM_000548 | c.1288A>T | p.R430X | stopgain | 0.35052 |
| P239 | TSC2 | NM_000548 | c.3696dupT | p.S1232fs | frameshift insertion | 0.32735 |
| P242 | TSC2 | NM_000548 | c.775-2A>T | - | - | 0.29561 |
| P242 | TSC2 | NM_000548 | c.775-2A>T | - | - | 0.29561 |
| P268 | TSC2 | NM_000548 | c.975+1G>T | - | - | 0.22358 |
| P10 | TSC2 | NM_000548 | c.1947-2A>T | - | - | 0.33984 |
| P33 | TSHR | NM_000369 | c.1558T>A | p.W520R | nonsynonymous SNV | 0.04662 |
| P167 | TSHR | NM_000369 | c.1970G>A | p.S657N | nonsynonymous SNV | 0.11934 |
| P153 | TSHZ3 | NM_020856 | c.1754C>A | p.P585Q | nonsynonymous SNV | 0.03601 |
| P229 | TSHZ3 | NM_020856 | c.1634G>T | p.G545V | nonsynonymous SNV | 0.04864 |
| P161 | TYRO3 | NM_006293 | c.1382+2T>C | - | - | 0.02646 |
| P153 | U2AF1 | NM_006758 | c.101C>T | p.S34F | nonsynonymous SNV | 0.17216 |
| P191 | UPF1 | NM_001297549 | c.487G>C | p.A163P | nonsynonymous SNV | 0.27326 |
| P213 | UPF1 | NM_001297549 | c.829C>A | p.L277M | nonsynonymous SNV | 0.0122 |
| P188 | UPF1 | NM_001297549 | c.833A>G | p.E278G | nonsynonymous SNV | 0.01352 |
| P78 | VEGFA | NM_001171624 | c.245G>A | p.R82Q | nonsynonymous SNV | 0.02007 |
| P63 | VEGFA | - | - | - | gain | 4.535 |
| P66 | VEGFA | - | - | - | gain | 6.287 |
| P123 | VHL | NM_198156 | c.350T>A | p.L117Q | nonsynonymous SNV | 0.43737 |
| P73 | VHL | NM_198156 | c.233A>G | p.N78S | nonsynonymous SNV | 0.24815 |
| P150 | VHL | NM_000551 | c.349T>A | p.W117R | nonsynonymous SNV | 0.12062 |
| P87 | VHL | NM_198156 | c.334T>A | p.Y112N | nonsynonymous SNV | 0.02092 |
| P249 | VHL | NM_000551 | c.341G>T | p.G114V | nonsynonymous SNV | 0.09213 |
| P69 | VHL | NM_198156 | c.266T>G | p.L89R | nonsynonymous SNV | 0.12433 |
| P93 | VHL | NM_000551 | c.463G>C | p.V155L | nonsynonymous SNV | 0.06335 |
| P224 | VHL | NM_198156 | c.194C>T | p.S65L | nonsynonymous SNV | 0.31199 |
| P140 | VHL | NM_198156 | c.362G>A | p.C121Y | nonsynonymous SNV | 0.21053 |
| P232 | VHL | NM_198156 | c.302T>G | p.L101R | nonsynonymous SNV | 0.11704 |
| P131 | VHL | NM_198156 | c.257C>T | p.P86L | nonsynonymous SNV | 0.09016 |
| P32 | VHL | NM_198156 | c.362G>A | p.C121Y | nonsynonymous SNV | 0.17742 |
| P48 | VHL | NM_198156 | c.340G>C | p.V114L | nonsynonymous SNV | 0.30816 |
| P186 | VHL | NM_198156 | c.266T>C | p.L89P | nonsynonymous SNV | 0.20272 |
| P246 | VHL | NM_198156 | c.320G>C | p.R107P | nonsynonymous SNV | 0.20509 |
| P207 | VHL | NM_000551 | c.361G>T | p.D121Y | nonsynonymous SNV | 0.10558 |
| P169 | VHL | NM_000551 | c.351G>C | p.W117C | nonsynonymous SNV | 0.10123 |
| P81 | VHL | NM_000551 | c.349T>A | p.W117R | nonsynonymous SNV | 0.44416 |
| P117 | VHL | NM_198156 | c.266T>A | p.L89H | nonsynonymous SNV | 0.07664 |
| P84 | VHL | NM_000551 | c.343C>T | p.H115Y | nonsynonymous SNV | 0.11538 |
| P230 | VHL | NM_000551 | c.388G>T | p.V130F | nonsynonymous SNV | 0.18847 |
| P180 | VHL | NM_000551 | c.461C>T | p.P154L | nonsynonymous SNV | 0.21277 |
| P204 | VHL | NM_198156 | c.349C>G | p.L117V | nonsynonymous SNV | 0.02236 |
| P60 | VHL | NM_000551 | c.388G>C | p.V130L | nonsynonymous SNV | 0.37238 |
| P219 | VHL | NM_000551 | c.383T>A | p.L128H | nonsynonymous SNV | 0.23944 |
| P250 | VHL | NM_198156 | c.263G>T | p.W88L | nonsynonymous SNV | 0.30357 |
| P251 | VHL | NM_000551 | c.393C>G | p.N131K | nonsynonymous SNV | 0.23158 |
| P129 | VHL | NM_000551 | c.349T>A | p.W117R | nonsynonymous SNV | 0.53869 |
| P178 | VHL | NM_198156 | c.292T>A | p.Y98N | nonsynonymous SNV | 0.08475 |
| P198 | VHL | NM_000551 | c.361G>T | p.D121Y | nonsynonymous SNV | 0.12132 |
| P122 | VHL | NM_198156 | c.266T>A | p.L89H | nonsynonymous SNV | 0.07702 |
| P97 | VHL | NM_000551 | c.463G>T | p.V155L | nonsynonymous SNV | 0.35024 |
| P89 | VHL | NM_000551 | c.385C>G | p.L129V | nonsynonymous SNV | 0.02362 |
| P89 | VHL | NM_000551 | c.388G>C | p.V130L | nonsynonymous SNV | 0.07321 |
| P112 | VHL | NM_198156 | c.326T>A | p.I109N | nonsynonymous SNV | 0.16993 |
| P146 | VHL | NM_000551 | c.344A>T | p.H115L | nonsynonymous SNV | 0.40644 |
| P127 | VHL | NM_198156 | c.266T>A | p.L89H | nonsynonymous SNV | 0.22258 |
| P133 | VHL | NM_198156 | c.239G>A | p.S80N | nonsynonymous SNV | 0.10856 |
| P247 | VHL | NM_198156 | c.410T>A | p.L137Q | nonsynonymous SNV | 0.05664 |
| P189 | VHL | NM_198156 | c.350T>A | p.L117Q | nonsynonymous SNV | 0.09859 |
| P85 | VHL | NM_000551 | c.362A>G | p.D121G | nonsynonymous SNV | 0.25092 |
| P216 | VHL | NM_198156 | c.383T>C | p.L128P | nonsynonymous SNV | 0.16639 |
| P151 | VHL | NM_198156 | c.240T>A | p.S80R | nonsynonymous SNV | 0.11501 |
| P228 | VHL | NM_198156 | c.263G>T | p.W88L | nonsynonymous SNV | 0.25833 |
| P237 | VHL | NM_000551 | c.350G>T | p.W117L | nonsynonymous SNV | 0.44373 |
| P90 | VHL | NM_198156 | c.287A>C | p.Q96P | nonsynonymous SNV | 0.19492 |
| P175 | VHL | NM_198156 | c.340G>T | p.V114L | nonsynonymous SNV | 0.2597 |
| P36 | VHL | NM_000551 | c.452T>C | p.I151T | nonsynonymous SNV | 0.2524 |
| P120 | VHL | NM_198156 | c.368A>T | p.Q123L | nonsynonymous SNV | 0.48848 |
| P91 | VHL | NM_000551 | c.451A>T | p.I151F | nonsynonymous SNV | 0.25922 |
| P21 | VHL | NM_000551 | c.349T>A | p.W117R | nonsynonymous SNV | 0.38408 |
| P95 | VHL | NM_198156 | c.333C>A | p.S111R | nonsynonymous SNV | 0.26104 |
| P152 | VHL | NM_198156 | c.377G>T | p.R126L | nonsynonymous SNV | 0.22569 |
| P188 | VHL | NM_198156 | c.350T>A | p.L117Q | nonsynonymous SNV | 0.222 |
| P209 | VHL | NM_198156 | c.368A>T | p.Q123L | nonsynonymous SNV | 0.01667 |
| P252 | VHL | NM_198156 | c.350T>A | p.L117Q | nonsynonymous SNV | 0.01012 |
| P165 | VHL | NM_198156 | c.350T>C | p.L117P | nonsynonymous SNV | 0.3913 |
| P200 | VHL | NM_198156 | c.266T>A | p.L89H | nonsynonymous SNV | 0.26137 |
| P155 | VHL | NM_198156 | c.350T>C | p.L117P | nonsynonymous SNV | 0.29724 |
| P86 | VHL | NM_000551 | c.405_406insTT | p.L135fs | frameshift insertion | 0.17193 |
| P159 | VHL | NM_000551 | c.347dupT | p.L116fs | frameshift insertion | 0.33043 |
| P124 | VHL | NM_000551 | c.357_370del | p.F119fs | frameshift deletion | 0.31083 |
| P238 | VHL | NM_000551 | c.350G>A | p.W117X | stopgain | 0.20737 |
| P142 | VHL | NM_198156 | c.346delA | p.T116fs | frameshift deletion | 0.10638 |
| P115 | VHL | NM_198156 | c.425C>A | p.S142X | stopgain | 0.11481 |
| P150 | VHL | NM_000551 | c.440delT | p.I147fs | frameshift deletion | 0.14851 |
| P111 | VHL | NM_198156 | c.286C>T | p.Q96X | stopgain | 0.29707 |
| P109 | VHL | NM_198156 | c.314_332del | p.T105fs | frameshift deletion | 0.20964 |
| P177 | VHL | NM_198156 | c.367_374del | p.Q123fs | frameshift deletion | 0.31081 |
| P20 | VHL | NM_198156 | c.440delT | p.L147fs | frameshift deletion | 0.28349 |
| P39 | VHL | NM_198156 | c.189_195del | p.L63fs | frameshift deletion | 0.06146 |
| P83 | VHL | NM_198156 | c.211delC | p.P71fs | frameshift deletion | 0.31361 |
| P225 | VHL | NM_000551 | c.405_406insTT | p.L135fs | frameshift insertion | 0.27381 |
| P76 | VHL | NM_198156 | c.264G>A | p.W88X | stopgain | 0.22739 |
| P222 | VHL | NM_000551 | c.378_385del | p.D126fs | frameshift deletion | 0.28829 |
| P38 | VHL | NM_198156 | c.445dupG | p.E148fs | frameshift insertion | 0.4071 |
| P190 | VHL | NM_198156 | c.387delC | p.V129fs | frameshift deletion | 0.20506 |
| P234 | VHL | NM_198156 | c.209dupA | p.E70fs | frameshift insertion | 0.27548 |
| P37 | VHL | NM_198156 | c.294C>A | p.Y98X | stopgain | 0.37079 |
| P156 | VHL | NM_198156 | c.341_347del | p.V114fs | frameshift deletion | 0.33992 |
| P56 | VHL | NM_000551 | c.440delT | p.I147fs | frameshift deletion | 0.20542 |
| P77 | VHL | NM_000551 | c.440dupT | p.I147fs | frameshift insertion | 0.66176 |
| P49 | VHL | NM_198156 | c.423_430del | p.R141fs | frameshift deletion | 0.27258 |
| P49 | VHL | NM_198156 | c.432delC | p.Y144X | stopgain | 0.28299 |
| P182 | VHL | NM_198156 | c.185_201del | p.V62fs | frameshift deletion | 0.29844 |
| P161 | VHL | NM_198156 | c.297dupA | p.P99fs | frameshift insertion | 0.4441 |
| P132 | VHL | NM_198156 | c.327dupC | p.I109fs | frameshift insertion | 0.11523 |
| P136 | VHL | NM_198156 | c.410_423del | p.L137fs | frameshift deletion | 0.06738 |
| P50 | VHL | NM_000551 | c.400G>T | p.E134X | stopgain | 0.19251 |
| P116 | VHL | NM_000551 | c.450dupT | p.N150fs | frameshift insertion | 0.37129 |
| P134 | VHL | NM_198156 | c.479_480insA | p.L160fs | frameshift insertion | 0.35769 |
| P266 | VHL | NM_198156 | c.268dupA | p.L89fs | frameshift insertion | 0.09987 |
| P233 | VHL | NM_198156 | c.398_399del | p.N133fs | frameshift deletion | 0.32629 |
| P210 | VHL | NM_000551 | c.463_463del | p.V155fs | frameshift deletion | 0.24084 |
| P244 | VHL | NM_198156 | c.402C>G | p.Y134X | stopgain | 0.57346 |
| P259 | VHL | NM_198156 | c.332dupG | p.S111fs | frameshift insertion | 0.21192 |
| P16 | VHL | NM_000551 | c.420_421insAA | p.L140fs | frameshift insertion | 0.33047 |
| P99 | VHL | NM_198156 | c.181delC | p.P61fs | frameshift deletion | 0.33959 |
| P88 | VHL | NM_198156 | c.397delA | p.N133fs | frameshift deletion | 0.34431 |
| P199 | VHL | NM_000551 | c.347delT | p.L116fs | frameshift deletion | 0.76712 |
| P245 | VHL | NM_198156 | c.433delG | p.E145fs | frameshift deletion | 0.31156 |
| P235 | VHL | NM_198156 | c.208G>T | p.E70X | stopgain | 0.30769 |
| P260 | VHL | NM_000551 | c.450delT | p.N150fs | frameshift deletion | 0.21656 |
| P78 | VHL | NM_198156 | c.386delT | p.V129fs | frameshift deletion | 0.24361 |
| P191 | VHL | NM_198156 | c.229_250del | p.C77fs | frameshift deletion | 0.31701 |
| P185 | VHL | NM_198156 | c.394G>T | p.E132X | stopgain | 0.39766 |
| P220 | VHL | NM_000551 | c.412delC | p.P138fs | frameshift deletion | 0.17492 |
| P130 | VHL | NM_000551 | c.406dupT | p.L135fs | frameshift insertion | 0.20508 |
| P168 | VHL | NM_198156 | c.476dupG | p.R159fs | frameshift insertion | 0.29187 |
| P163 | VHL | NM_198156 | c.399delT | p.N133fs | frameshift deletion | 0.38671 |
| P106 | VHL | NM_198156 | c.169delG | p.G57fs | frameshift deletion | 0.28907 |
| P214 | VHL | NM_198156 | c.402C>G | p.Y134X | stopgain | 0.22917 |
| P172 | VHL | NM_198156 | c.283_286del | p.P95fs | frameshift deletion | 0.43233 |
| P172 | VHL | NM_198156 | c.288_289del | p.Q96fs | frameshift deletion | 0.43601 |
| P114 | VHL | NM_000551 | c.430delG | p.G144fs | frameshift deletion | 0.11776 |
| P90 | VHL | NM_198156 | c.217C>T | p.Q73X | stopgain | 0.17883 |
| P35 | VHL | NM_198156 | c.226_227insC | p.F76fs | frameshift insertion | 0.37904 |
| P35 | VHL | NM_198156 | c.230delG | p.C77fs | frameshift deletion | 0.37665 |
| P36 | VHL | NM_000551 | c.439_445del | p.I147fs | frameshift deletion | 0.24375 |
| P74 | VHL | NM_198156 | c.386delT | p.V129fs | frameshift deletion | 0.31347 |
| P213 | VHL | NM_198156 | c.463dupA | p.Q154fs | frameshift insertion | 0.52564 |
| P72 | VHL | NM_000551 | c.418_419insTCAA | p.L140fs | frameshift insertion | 0.24348 |
| P128 | VHL | NM_198156 | c.397_400del | p.N133fs | frameshift deletion | 0.37795 |
| P145 | VHL | NM_198156 | c.301_329del | p.L101fs | frameshift deletion | 0.09441 |
| P194 | VHL | NM_198156 | c.286dupC | p.P95fs | frameshift insertion | 0.47547 |
| P218 | VHL | NM_198156 | c.343_344del | p.Y115fs | frameshift deletion | 0.26087 |
| P31 | VHL | NM_000551 | c.343_344del | p.H115fs | frameshift deletion | 0.31746 |
| P162 | VHL | NM_198156 | c.176_186del | p.P59fs | frameshift deletion | 0.17325 |
| P135 | VHL | NM_000551 | c.371_380del | p.T124fs | frameshift deletion | 0.2796 |
| P269 | VHL | NM_198156 | c.308_309del | p.P103fs | frameshift deletion | 0.0507 |
| P269 | VHL | NM_198156 | c.312_313del | p.G104fs | frameshift deletion | 0.05026 |
| P183 | VHL | NM_000551 | c.440delT | p.I147fs | frameshift deletion | 0.05601 |
| P157 | VHL | NM_000551 | c.347dupT | p.L116fs | frameshift insertion | 0.47962 |
| P121 | VHL | NM_000551 | c.417delT | p.S139fs | frameshift deletion | 0.21149 |
| P26 | VHL | NM_198156 | c.165_178del | p.E55fs | frameshift deletion | 0.02586 |
| P171 | VHL | NM_000551 | c.440delT | p.I147fs | frameshift deletion | 0.37466 |
| P271 | VHL | NM_198156 | c.224_226del | p.75_76del | nonframeshift deletion | 0.14585 |
| P231 | VHL | NM_198156 | c.224_226del | p.75_76del | nonframeshift deletion | 0.20905 |
| P102 | VHL | NM_198156 | c.224_226del | p.75_76del | nonframeshift deletion | 0.17026 |
| P158 | VHL | NM_198156 | c.341-2A>G | - | - | 0.24806 |
| P187 | VHL | NM_000551 | c.341-2A>G | - | - | 0.10963 |
| P181 | VHL | NM_000551 | c.341-2A>C | - | - | 0.21739 |
| P101 | VHL | NM_198156 | c.341-1G>A | - | - | 0.22176 |
| P236 | VHL | NM_198156 | c.340+2T>A | - | - | 0.29008 |
| P148 | VHL | NM_198156 | c.341-1G>C | - | - | 0.54386 |
| P54 | VHL | - | - | - | loss | 0.965 |
| P6 | VHL | - | - | - | loss | 1.033 |
| P235 | WEE1 | NM_003390 | c.656A>T | p.K219I | nonsynonymous SNV | 0.03642 |
| P164 | WEE1 | NM_003390 | c.941T>G | p.F314C | nonsynonymous SNV | 0.21856 |
| P24 | WISP3 | NM_003880 | c.367G>A | p.E123K | nonsynonymous SNV | 0.01196 |
| P220 | WISP3 | NM_003880 | c.460C>T | p.P154S | nonsynonymous SNV | 0.05311 |
| P168 | WISP3 | NM_003880 | c.712T>A | p.C238S | nonsynonymous SNV | 0.21797 |
| P6 | WRN | NM_000553 | c.2704T>C | p.Y902H | nonsynonymous SNV | 0.58163 |
| P63 | WRN | - | - | - | loss | 0.379 |
| P5 | WRN | - | - | - | loss | 0.006 |
| P201 | WT1 | NM_000378 | c.1159A>T | p.K387X | stopgain | 0.1719 |
| P165 | XIRP2 | NM_001199144 | c.4168C>A | p.L1390I | nonsynonymous SNV | 0.02086 |
| P147 | XIRP2 | NM_001199144 | c.2780T>A | p.L927X | stopgain | 0.06545 |
| P15 | XIRP2 | NM_001199144 | c.4259_4260del | p.N1420fs | frameshift deletion | 0.07521 |
| P171 | XIRP2 | NM_001199144 | c.3643C>T | p.R1215X | stopgain | 0.27766 |
| P111 | XRCC2 | NM_005431 | c.19A>T | p.R7W | nonsynonymous SNV | 0.21695 |
| P59 | XRCC4 | NM_022550 | c.225G>T | p.L75F | nonsynonymous SNV | 0.03963 |
| P43 | YAP1 | NM_001130145 | c.880G>A | p.V294I | nonsynonymous SNV | 0.01869 |
| P232 | ZBTB16 | NM_006006 | c.1219A>T | p.S407C | nonsynonymous SNV | 0.03337 |
| P94 | ZFHX3 | NM_001164766 | c.8090A>C | p.H2697P | nonsynonymous SNV | 0.02523 |
| P2 | ZFHX3 | NM_001164766 | c.8090A>C | p.H2697P | nonsynonymous SNV | 0.01578 |
| P143 | ZFHX3 | NM_001164766 | c.8090A>C | p.H2697P | nonsynonymous SNV | 0.01527 |
| P75 | ZFHX3 | NM_001164766 | c.8090A>C | p.H2697P | nonsynonymous SNV | 0.02413 |
| P93 | ZFHX3 | NM_001164766 | c.3505G>A | p.V1169M | nonsynonymous SNV | 0.11313 |
| P42 | ZFHX3 | NM_001164766 | c.8111C>A | p.P2704Q | nonsynonymous SNV | 0.01667 |
| P179 | ZFHX3 | NM_001164766 | c.3410T>G | p.L1137R | nonsynonymous SNV | 0.0247 |
| P38 | ZFHX3 | NM_001164766 | c.5490C>G | p.N1830K | nonsynonymous SNV | 0.02079 |
| P38 | ZFHX3 | NM_001164766 | c.5485T>A | p.Y1829N | nonsynonymous SNV | 0.02119 |
| P33 | ZFHX3 | NM_001164766 | c.8090A>C | p.H2697P | nonsynonymous SNV | 0.02424 |
| P81 | ZFHX3 | NM_001164766 | c.173C>T | p.S58L | nonsynonymous SNV | 0.02284 |
| P78 | ZFHX3 | NM_001164766 | c.8089C>T | p.H2697Y | nonsynonymous SNV | 0.02019 |
| P89 | ZFHX3 | NM_001164766 | c.5078C>T | p.P1693L | nonsynonymous SNV | 0.11094 |
| P164 | ZFHX3 | NM_001164766 | c.1994A>C | p.E665A | nonsynonymous SNV | 0.0223 |
| P135 | ZFHX3 | NM_001164766 | c.8090A>C | p.H2697P | nonsynonymous SNV | 0.01802 |
| P135 | ZFHX3 | NM_001164766 | c.5474A>G | p.K1825R | nonsynonymous SNV | 0.28653 |
| P3 | ZFHX4 | NM_024721 | c.904C>G | p.R302G | nonsynonymous SNV | 0.1236 |
| P61 | ZFHX4 | NM_024721 | c.123C>G | p.D41E | nonsynonymous SNV | 0.03299 |
| P120 | ZFHX4 | NM_024721 | c.4297A>T | p.S1433C | nonsynonymous SNV | 0.29505 |
| P144 | ZFHX4 | NM_024721 | c.143A>T | p.D48V | nonsynonymous SNV | 0.39577 |
| P189 | ZFHX4 | NM_024721 | c.6472C>T | p.Q2158X | stopgain | 0.01205 |
| P270 | ZFHX4 | NM_024721 | c.9053delC | p.A3018fs | frameshift deletion | 0.30098 |
| P135 | ZIC1 | NM_003412 | c.713C>A | p.P238H | nonsynonymous SNV | 0.31802 |
| P166 | ZNF423 | NM_001271620 | c.1991T>A | p.L664Q | nonsynonymous SNV | 0.06274 |
| P116 | ZNF423 | NM_001271620 | c.3235A>T | p.M1079L | nonsynonymous SNV | 0.23625 |
| P233 | ZNF423 | NM_001271620 | c.2554C>T | p.R852W | nonsynonymous SNV | 0.0304 |
| P101 | ZNF423 | NM_001271620 | c.2863A>T | p.N955Y | nonsynonymous SNV | 0.175 |
| P222 | ZNF536 | NM_014717 | c.671T>G | p.L224R | nonsynonymous SNV | 0.02232 |
| P37 | ZNF536 | NM_014717 | c.2635G>T | p.E879X | stopgain | 0.20991 |
| P210 | ZNF703 | NM_025069 | c.76A>T | p.R26W | nonsynonymous SNV | 0.24935 |
| P5 | ZNF703 | NM_025069 | c.604T>C | p.F202L | nonsynonymous SNV | 0.03251 |
| P38 | ZNF804A | NM_194250 | c.935G>T | p.C312F | nonsynonymous SNV | 0.02434 |
| P209 | ZNF804A | NM_194250 | c.3413T>A | p.L1138H | nonsynonymous SNV | 0.01233 |
| P85 | ZNF804B | NM_181646 | c.3227C>A | p.A1076D | nonsynonymous SNV | 0.02139 |
| P76 | ZNF831 | NM_178457 | c.532G>A | p.G178S | nonsynonymous SNV | 0.07711 |
| P200 | ZNF831 | NM_178457 | c.518C>T | p.P173L | nonsynonymous SNV | 0.03337 |
| P15 | ZNF831 | NM_178457 | c.1622G>A | p.R541H | nonsynonymous SNV | 0.16739 |
| P43 | ZNF831 | NM_178457 | c.146dupC | p.A49fs | frameshift insertion | 0.018 |


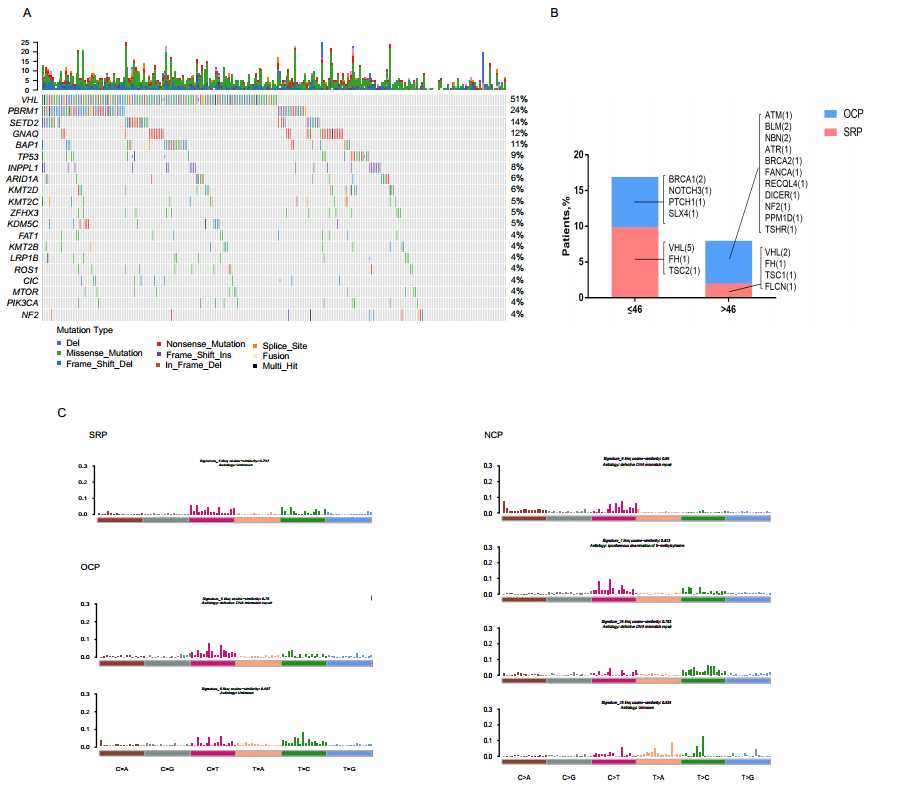


Supplementary Figure 1. Spectrum of somatic mutations of patient with RCC, percentage of mutated patients in age≤46 and >46 years and mutation signature of patient with RCC. A. Heatmap of somatic mutations in 274 patients with RCC. Top 20 genes across samples are presented with the corresponding frequencies on the right. B. Characteristics of PGVs in patients below and over 46 years old. C. Mutational signature of patients with RCC in SRP, OCP and NCP groups. SRP, Syndromic RCC-associated gene PGVs; OCP, Other Cancer-associated gene PGVs; NCP, Non-Cancer-associated gene PGVs.
